# Amino acid motifs in natural products: synthesis of *O*-acylated derivatives of (2*S*,3*S*)-3-hydroxyleucine

**DOI:** 10.3762/bjoc.10.113

**Published:** 2014-05-16

**Authors:** Oliver Ries, Martin Büschleb, Markus Granitzka, Dietmar Stalke, Christian Ducho

**Affiliations:** 1 Department of Chemistry, Institute of Organic and Biomolecular Chemistry, Georg-August-University Göttingen, Tammannstr. 2, 37077 Göttingen, Germany; 2 Department of Chemistry, Institute of Inorganic Chemistry, Georg-August-University Göttingen, Tammannstr. 4, 37077 Göttingen, Germany; 3 Department of Pharmacy, Pharmaceutical and Medicinal Chemistry, Saarland University, Campus C2 3, 66123 Saarbrücken, Germany

**Keywords:** chiral pool, cross metathesis, esterification, β-hydroxy-α-amino acids, natural products

## Abstract

(2*S*,3*S*)-3-Hydroxyleucine can be found in an increasing number of bioactive natural products. Within the context of our work regarding the total synthesis of muraymycin nucleoside antibiotics, we have developed a synthetic approach towards (2*S*,3*S*)-3-hydroxyleucine building blocks. Application of different protecting group patterns led to building blocks suitable for *C*- or *N*-terminal derivatization as well as for solid-phase peptide synthesis. With respect to according motifs occurring in natural products, we have converted these building blocks into 3-*O*-acylated structures. Utilizing an esterification and cross-metathesis protocol, (2*S*,3*S*)-3-hydroxyleucine derivatives were synthesized, thus opening up an excellent approach for the synthesis of bioactive natural products and derivatives thereof for structure activity relationship (SAR) studies.

## Introduction

**Figure 1:**
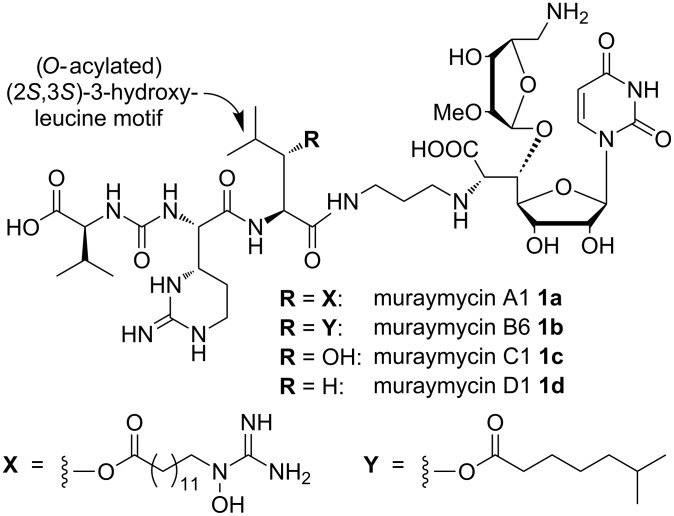
Structures of muraymycins A1, B6, C1 and D1 **1a**–**d**.

Besides the proteinogenic β-hydroxy-α-amino acids serine and threonine, (2*S*,3*S*)-3-hydroxyleucine can be found as a substructure of several bioactive natural products. This structural motif often serves as a 'three-way-junction', as for instance in azinothricin [1], citropeptin [2], kettapeptin [3], pipalamycin [4], dentigerumycin [5], as monosulfuric acid ester [6] or as potential acylation site for fatty acid side chains such as in A- and B-series muraymycin nucleoside antibiotics (Figure 1) [7–9]. In the case of these muraymycin congeners, acylation of the 3-hydroxy position with fatty acid side chains, which are ω-functionalized in the A-series, leads to a significant increase in biological activity. While non-acylated muraymycins C1 (**1c**) and D1 (**1d**) showed a minimal inhibitory concentration (MIC) value of 1 µg/mL (*E. coli*), the attached unfunctionalized acyl side chain present in muraymycin B6 (**1b**) led to an increase of potency up to MIC = 0.06 µg/mL, which was therefore nearly as active as the most active naturally occurring muraymycin A1 (**1a**, MIC = 0.03 µg/mL). For other bacteria such as *S. aureus*, the presence of the 3-*O*-acyl motif can even turn biologically inactive C- and D-series muraymycins **1c**,**d** into active derivatives **1a**,**b** of the A- and B-series, respectively (Figure 1) [7]. This significant change in potency might be owed to increased lipophilicity and thus to improved cellular uptake, as muraymycins inhibit the transmembrane protein MraY (translocase I), an enzyme involved in peptidoglycan formation with its active site located on the cytosolic side of the membrane [10–14]. For previously reported SAR studies on muraymycins and their analogues, the fatty acid side chain was either neglegted or replaced with a structurally distinct lipophilic mimic [15–17]. Mansour and co-workers found a direct relation of the lipophilicity of semi-synthetic muraymycin C1 analogues and their biological activity [18]. They thus also postulated that the lipid structure might be involved in transporting the molecule to the active site of the target enzyme. The most recent SAR investigation on synthetic muraymycin analogues conducted by Ichikawa et al. also supported the proposal of increased membrane penetration resulting from the lipophilic moiety [17]. However, they employed simple lipophilic amino acids as surrogates of the naturally occurring *O*-acylated (2*S*,3*S*)-3-hydroxyleucine motif. This led to antibacterially active compounds, but one of it showed a ca. 30-fold reduction of inhibitory potency towards the target enzyme MraY as compared to non-lipidated muraymycin D2 containing L-leucine instead of (2*S*,3*S*)-3-hydroxyleucine. This result highlighted the significance of the native linkage of the lipophilic moiety to the muraymycin scaffold.

As part of our synthetic studies on muraymycins and their analogues [19–24], including investigations on the unusual ω-functionalized fatty acid motif found in muraymycin A1 (**1a**) [25], we identified the need for a highly stereoselective synthesis of (2*S*,3*S*)-3-hydroxyleucine and its *O*-acylated derivatives. Efficient synthetic access to such compounds will enable the unprecedented preparation of lipidated muraymycins bearing the native linkage of the fatty acid moieties, thus potentially leading to novel congeners with improved biological activity. It will also be useful for the synthesis of other natural products containing this amino acid.

Several synthetic approaches towards (2*S*,3*S*)-3-hydroxyleucine have been established utilizing for instance diastereoselective aldol reactions [26–28], Sharpless epoxidation [29], asymmetric hydrogenation [30] and dynamic kinetic resolution [31]. However, in contrast to these routes, we desired to develop a synthesis of *O*-acylated (2*S*,3*S*)-3-hydroxyleucine derivatives which should be easily scalable and would enable *O*-acylation after construction of the stereocenters with only few changes in the protecting group pattern. The most promising strategy to achieve these goals appeared to be a variation of the ex-chiral pool synthesis developed by Zhu and co-workers [32]. They have utilized the stereocenter of D-serine in a diastereoselective Grignard addition to protected D-serinal as the key step of their route. In addition, we also envisaged to establish a facile strategy to introduce structural diversity in the *O*-acyl moiety, ideally by the late-stage derivatization of an according precursor.

## Results and Discussion

**Scheme 1:**
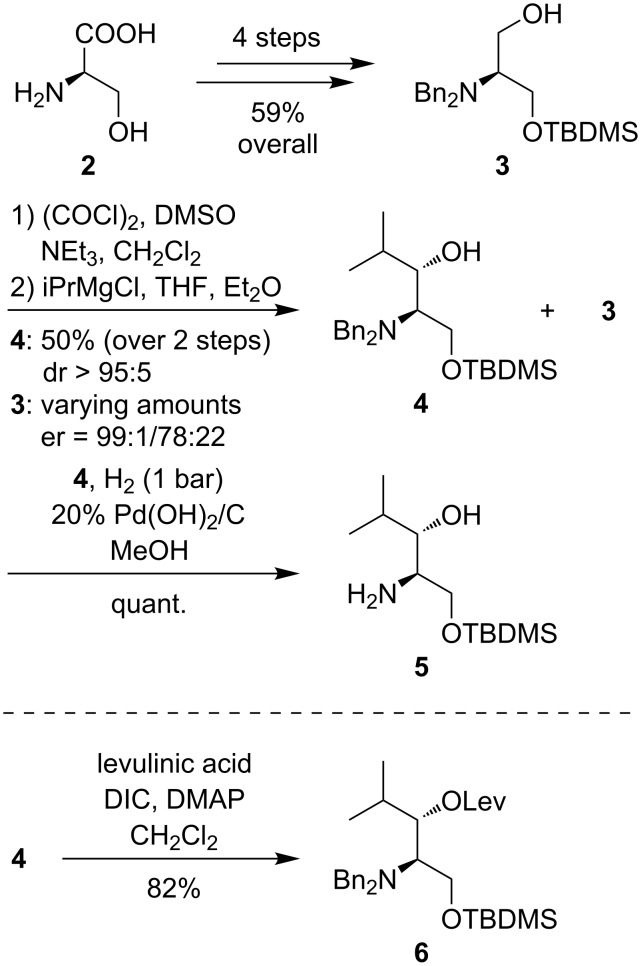
Synthesis of stereoisomerically pure amino alcohol **5** [32] and of derivative **6** suitable for X-ray crystallography.

Utilizing the aforementioned synthetic strategy developed by Zhu and co-workers [32], we have employed their 7-step synthesis of a stereoisomerically pure amino alcohol as the key intermediate. According to the previously reported protocol, D-serine (**2**) was transformed into protected derivative **3** (4 steps, 59% overall yield, Scheme 1). Alcohol **3** was then oxidized to a D-serinal derivative with limited stability, which was immediately converted into alcohol **4** by diastereoselective Felkin–Anh type Grignard addition with isopropyl magnesium chloride (50% yield over 2 steps from **3**, dr > 95:5). However, we could not confirm that the use of diethyl ether as co-solvent in the Grignard reaction would suppress the reformation of alcohol **3** as the competing Grignard reduction product, as it had been claimed by Zhu and co-workers in their initial report [32]. It was therefore decided to attempt the recycling of alcohol **3**, which was obtained in varying amounts, for another round of oxidation and Grignard addition. The enantiomeric purity of reisolated **3** was determined by HPLC analysis as its racemization can readily occur via silyl migration under the basic reaction conditions (see Supporting Information File 1 for HPLC chromatograms and for the synthesis of the racemic reference). After one oxidation–addition sequence we could isolate **3** with an enantiomeric purity of er = 99:1. Use of the obtained alcohol in a second oxidation–addition sequence followed by subsequent HPLC analysis of reduction product **3** demonstrated a decrease in enantiomeric purity to er = 78:22 though. On the basis of these results, we desisted from the use of this alcohol in a third oxidation–addition cycle. It was concluded that the recycling of the Grignard reduction product is in principle feasible, but that one should always check its enantiomeric purity prior to a repetition of the oxidation–addition sequence. Finally, the previously reported hydrogenolytic debenzylation of **4** [32] provided amino alcohol key intermediate **5** in quantitative yield for the deprotection step and in 29% overall yield over 7 steps from D-serine (**2**, Scheme 1). It should be noted that Garner's aldehyde is often used instead of acyclic D-serinal derivatives for the addition of nucleophiles to amino acid-derived aldehydes [33–34]. However, Zhu and co-workers have pointed out that Garner’s aldehyde surprisingly furnishes the *syn* diastereomer as the major product from its reaction with isopropyl magnesium chloride [32], thus discouraging its use for the preparation of *anti*-configured (2*S*,3*S*)-3-hydroxyleucine.

**Figure 2:**
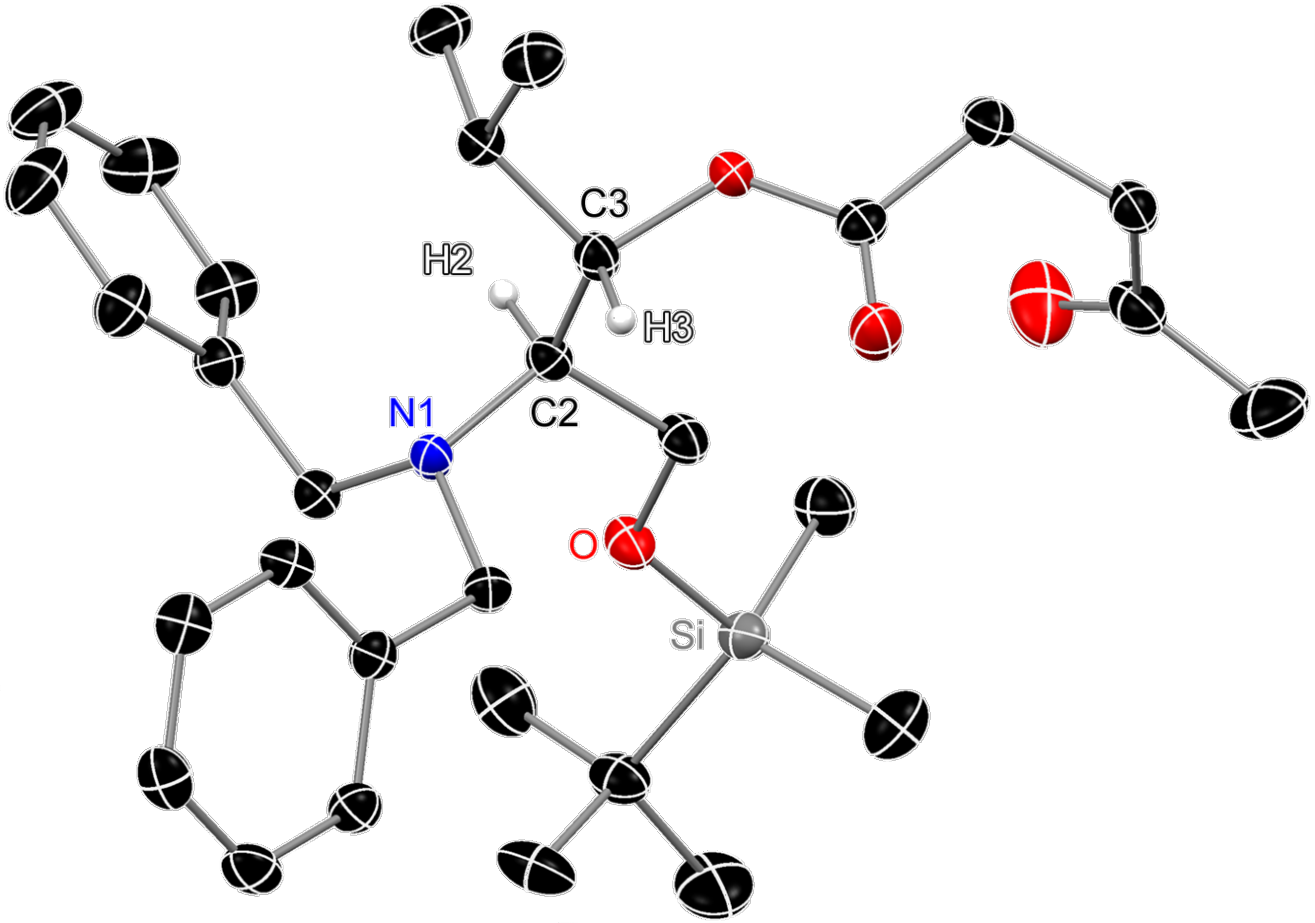
Molecular structure of levulinyl ester **6**. Anisotropic displacement parameters are depicted at the 50% probability level. Atom color code: carbon = black, oxygen = red, nitrogen = blue, silicon = grey, and hydrogen = white. The other hydrogen atoms are omitted for clarity.

Zhu and co-workers had based their stereochemical assignment of **5** on the ^1^H NMR coupling constants of a cyclic derivative of this amino alcohol. We were able to confirm the postulated stereochemical outcome of the Grignard addition by X-ray crystallography as (2*R*,3*S*) for the amino alcohol and thus, due to altered priorities, (2*S*,3*S*) for the corresponding amino acid, respectively. The absolute configuration was deduced assuming the integrity of the stereocenter at the 2-position, which had been derived from D-serine (**2**). As neither **4** nor **5** gave crystals suitable for X-ray analysis, the 3-hydroxy group of **4** was acylated with levulinic acid, leading to ester **6** in 82% yield (Scheme 1) which crystallized upon removal of residual solvent under reduced pressure. Hence, a single crystal of **6** was obtained, and X-ray crystal structure analysis unambiguously confirmed the relative configuration (Figure 2; see Supporting Information File 2 for crystallographic data). Levulinyl ester **6** crystallizes in the orthorhombic space group *P*2_1_2_1_2_1_ with one molecule in the asymmetric unit. The Flack parameter [35] refined to x = −0.05(8) and confirmes the stereocenters to have the proposed (2*R*,3*S*)-configuration.

In their synthesis of (2*S*,3*S*)-3-hydroxyleucine, Zhu and co-workers cyclized amino alcohol **5** towards an oxazolidinone, with the carbonyl group simultaneously providing *O*- and *N*-protection [32]. Removal of this protecting group required harsh reaction conditions though (conc. HCl, reflux, 40 h) and yielded the unprotected amino acid as the hydrochloric salt. However, in the case of our envisaged synthesis of 3-hydroxyleucine building blocks suitable for further derivatization as well as incorporation into natural products and their analogues, we were in need for a more versatile protecting group pattern. We therefore investigated different carbamates as protecting groups for the amino functionality, leading to the secondary alcohol functionality being selectively accessible for further conversions. We discovered that we were in need for a protecting group reducing the basic character of the amino group as an oxidation of the silyl-deprotected amino alcohol to the corresponding acid was not possible, probably due to the basicity of the amino group (reactions not displayed).

**Scheme 2:**
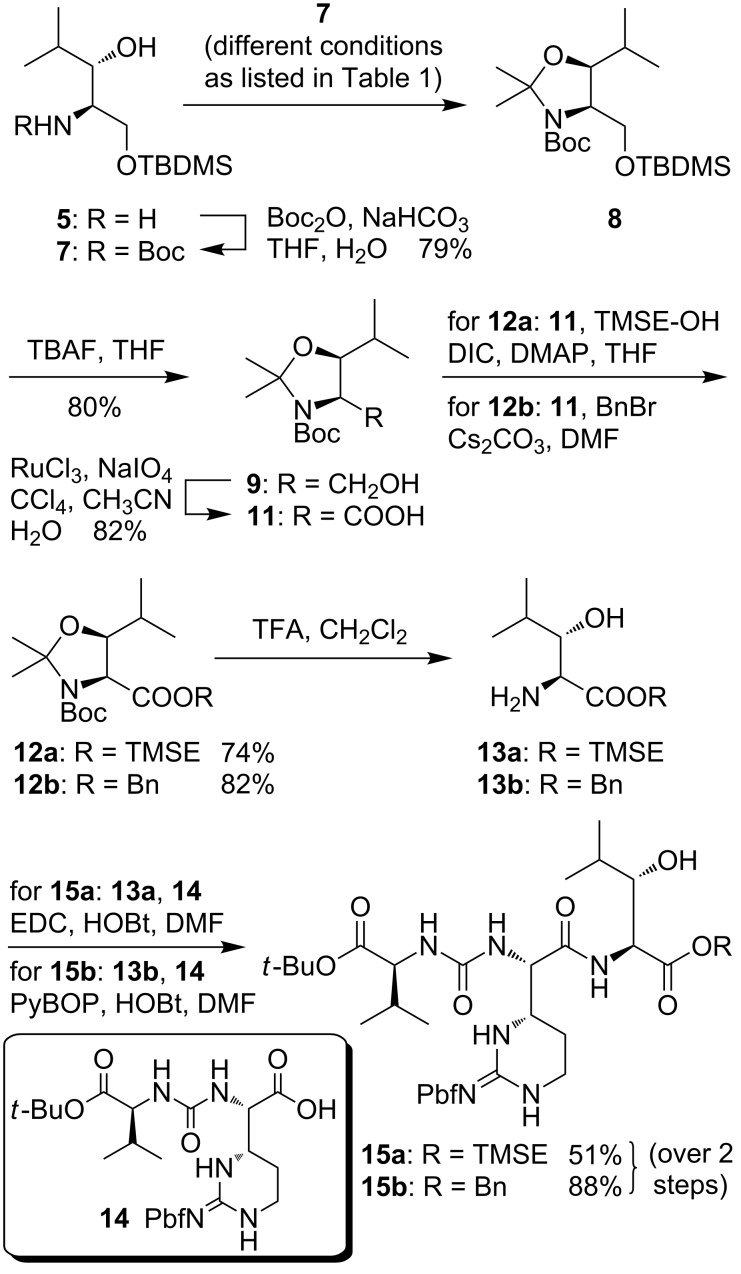
Synthesis of (2*S*,3*S*)-3-hydroxyleucine building blocks **13a**,**b** useful for *N*-derivatization and of the protected muraymycin tripeptide unit **15a**,**b**.

For the synthesis of a *C*-protected building block, **5** was converted with Boc anhydride to yield Boc-protected amino alcohol **7** in 79% yield (Scheme 2). After removal of the silyl group, a selective oxidation of the primary alcohol in presence of the unprotected 3-hydroxy group unfortunately could not be achieved (reactions not displayed). It was therefore decided to introduce an *N*,*O*-acetal moiety by transformation of **7** into the dimethyloxazolidine **8**, a reaction which required careful optimization though (Scheme 2, Table 1). Using boron trifluoride and 2,2-dimethoxypropane (2,2-DiMP), as known from the synthesis of the structurally related Garner's aldehyde [33–34], led to an unwanted cleavage of the silyl ether and furnished a mixture of the desired product **8**, the TBDMS cleavage product **9** and the corresponding *O*,*O*-acetal **10** (Table 1, entry 1). Hence, further reaction conditions using different acid catalysts and solvents were investigated. Changing the catalyst to pyridinium *p*-toluenesulfonate (PPTS) only led to small amounts of the product after prolonged reaction times and could not suppress the formation of the *O*,*O*-acetal (Table 1, entries 2 and 3). The use of racemic camphorsulfonic acid (CSA) in toluene and acetone, respectively, resulted in the formation of **8** in moderate to good yields (Table 1, entries 4–6). Finally, when 2,2-DiMP was used as solvent with catalytic amounts of CSA in the presence of magnesium sulfate, **8** could be isolated in a very good yield of 93% (Table 1, entry 7). After TBAF-mediated cleavage of the silyl ether, the resultant primary alcohol **9** (obtained in 80% yield) was oxidized to carboxylic acid **11** by ruthenium(III)-catalyzed periodate oxidation in 82% yield. Subsequent protection of the acid functionality furnished 2-(trimethylsilyl)ethyl (TMSE) and benzyl esters **12a** and **12b** (yields 72% and 84%, respectively). Finally, acidic deprotection afforded the desired building blocks **13a**,**b** suitable for *N*-derivatization, with the compounds not being fully purified. In order to demonstrate the synthetic versatility of these 3-hydroxyleucine derivatives, they were coupled with urea dipeptide **14** [36], thus providing protected derivatives **15a**,**b** of the full-length peptide unit of C-series muraymycins in yields of 51% and 88%, respectively, over 2 steps from **12a**,**b**. It is expected that **15a**,**b** can serve as useful building blocks in the total synthesis of muraymycins and their analogues.

**Table 1:** Optimization of the reaction of **7** to dimethyloxazolidine **8**.

Entry	Reaction conditions	Yield [%] (compound)

1	9 equiv 2,2-DiMP, 0.1 equiv BF_3_·Et_2_O, acetone, rt, 23 h	19 (**8**), 19 (**9**), 30 (**10**)^a^
2	9 equiv 2,2-DiMP, 0.1 equiv PPTS, acetone, rt, 4 d	76 (**7**), 21 (**8**)
3	10 equiv 2,2-DiMP, 0.3 equiv PPTS, THF, rt, 17 h	mixture (**8**)/(**9**)^b^, 9 (**10**)
4	26 equiv 2,2-DiMP^c^, 0.02 equiv CSA, MS 3 Å, toluene, 80 °C, 17 h	79 (**8**)
5	20 equiv 2,2-DiMP^d^, 0.02 equiv CSA, MS 3 Å, toluene, 80 °C, 15 h	74 (**7**), 20 (**8**)
6	30 equiv 2,2-DiMP, 0.03 equiv CSA, acetone, reflux, 16 h	60 (**8**)
7	0.15 equiv CSA, MgSO_4_, 2,2-DiMP, 50 °C, 24 h	93 (**8**)

^a^**10**:
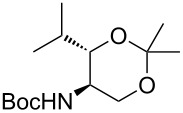
^b^not separated; ^c^2,2-dimethoxypropane (2,2-DiMP) was added in two portions; ^d^2,2-DiMP was added at once.

**Scheme 3:**
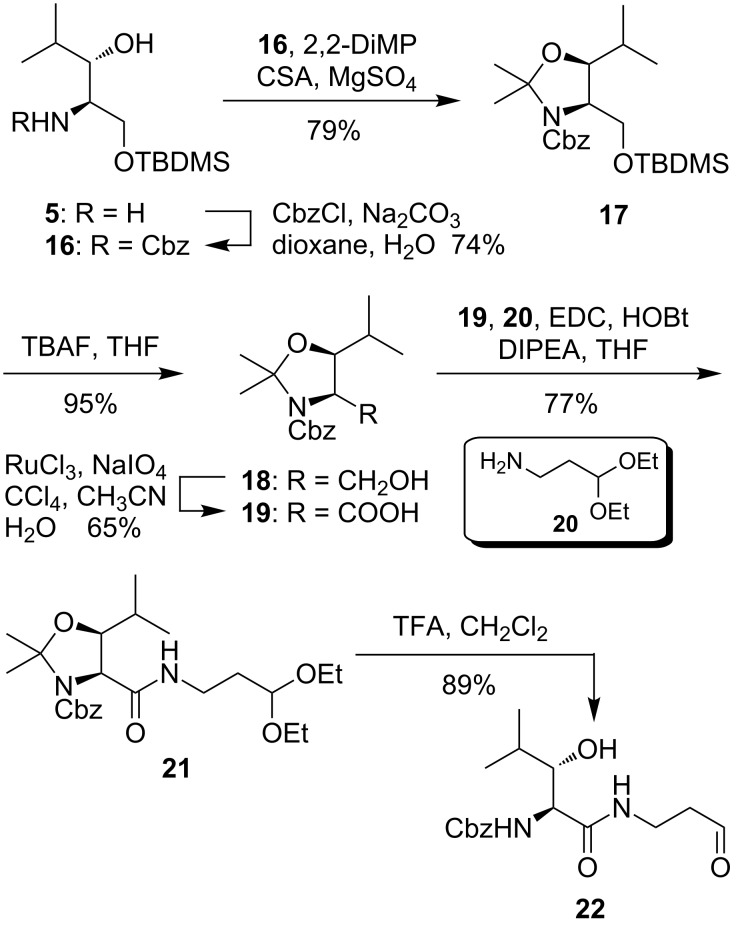
Synthesis of (2*S*,3*S*)-3-hydroxyleucine building block **19** useful for *C*-derivatization and of aldehyde **22**, a synthetic building block for C-series muraymycins.

Following the synthesis of building blocks **13a**,**b** suitable for *N*-derivatization, it was also desired to obtain (2*S*,3*S*)-3-hydroxyleucine derivatives suitable for *C*-terminal coupling reactions. We therefore chose a Cbz group as amino protecting group. Treatment of amino alcohol **5** with Cbz chloride furnished protected derivative **16** in 74% yield (Scheme 3). In analogy to the aforementioned synthetic route, **16** was then cyclized with 2,2-DiMP to the corresponding dimethyloxazolidine **17** (79% yield), followed by TBAF-mediated cleavage of the silyl ether (product **18**, 95% yield) and oxidation of the primary alcohol to carboxylic acid **19** in 65% yield (49% over 3 steps from **17**). The potential of **19** to serve as a universal building block for *C*-terminal derivatization was demonstrated by the following transformations. Acid **19** was reacted with 1,1-diethoxy-3-aminopropane (**20**) under standard EDC/HOBt coupling conditions to afford amide **21** in 77% yield, followed by concomitant acidic cleavage of the *N*,*O*- and *O*,*O*-acetal protecting groups leading to building block **22** in 89% yield (Scheme 3). Aldehyde **22** can be used for the connection of the (2*S*,3*S*)-3-hydroxyleucine motif of C-series muraymycins (such as muraymycin C1 (**1c**), Figure 1) to the nucleoside moiety by reductive amination (reactions not displayed), as demonstrated before in our syntheses of simplified muraymycin analogues [21–22].

**Scheme 4:**
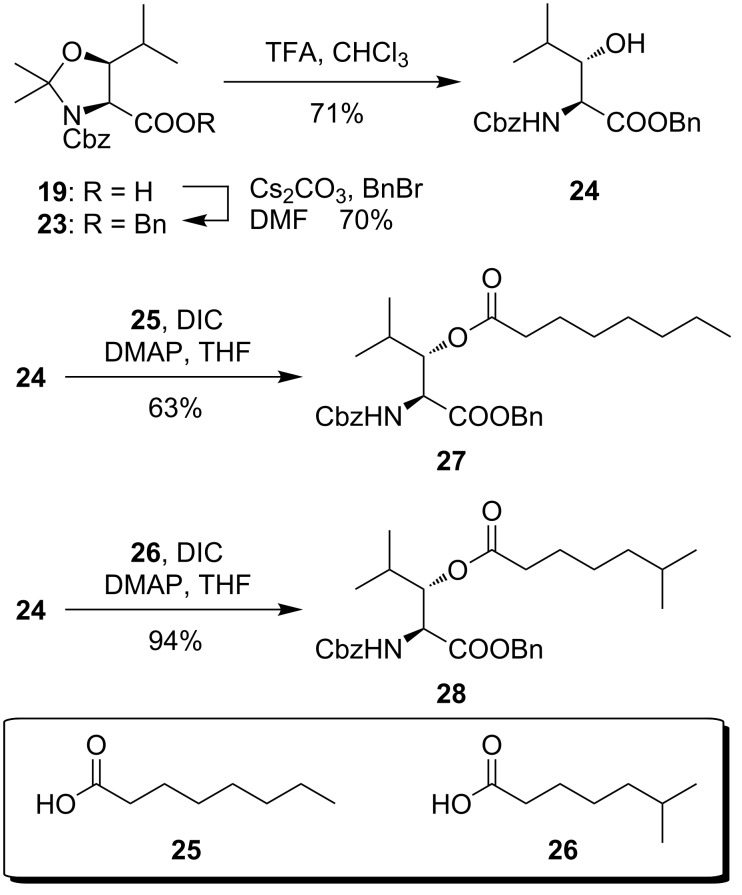
Synthesis of *O*-acylated (2*S*,3*S*)-3-hydroxyleucine derivatives **27** and **28**.

*N*-Cbz-protected acid **19** was also used for a methodical study regarding the acylation of the 3-hydroxy group. Therefore, **19** was converted into benzyl ester **23** (70% yield) followed by acidic cleavage of the acetonide to yield building block **24** in 71% yield (Scheme 4). Acylation reactions were carried out with DIC as coupling reagent and catalytic amounts of DMAP. Based on this protocol, esterification reactions with octanoic acid (**25**) and 6-methylheptanoic acid (**26**, vide infra) yielded the acylated (2*S*,3*S*)-3-hydroxyleucine derivatives **27** and **28** in 63% and 94% yield, respectively. The latter compound might serve as a building block for the synthesis of muraymycin B6 (**1b**) and also muraymycin B7 (as well as derivatives thereof) as the 6-methylheptanoyl moiety is a constituent of these natural products. It represents the first example of a synthetically obtained *O*-acylated 3-hydroxyleucine moiety of a naturally occurring muraymycin.

**Scheme 5:**
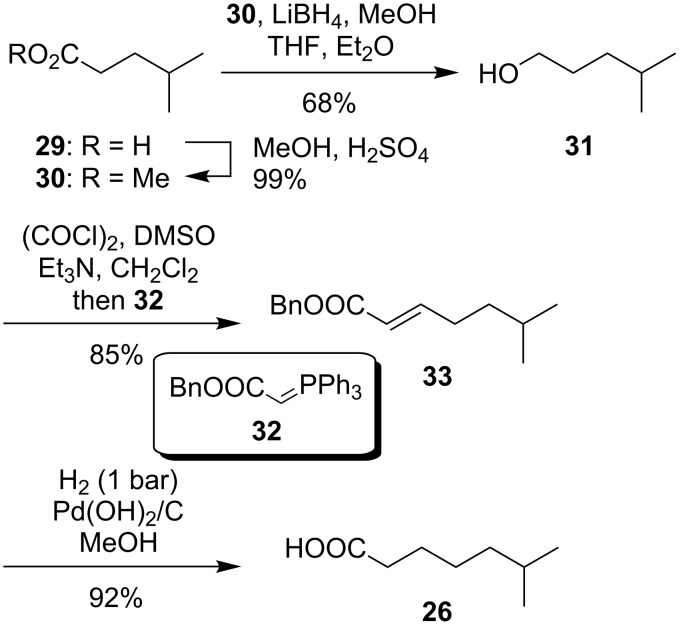
Synthesis of 6-methylheptanoic acid (**26**).

The preparation of *O*-acylated derivative **28** required the synthesis of 6-methylheptanoic acid (**26**) though. This branched carboxylic acid was obtained starting from commercially available 4-methylpentanoic acid (**29**, Scheme 5). Esterification with methanol (product **30**, 99% yield) and reduction of the ester with lithium borohydride afforded primary alcohol **31** in 68% yield. A direct reduction of acid **29** with lithium aluminum hydride was also investigated, but resulted in a moderate yield of 32% only (reaction not displayed). One-pot Swern oxidation to the corresponding aldehyde and subsequent Wittig reaction with stabilized Wittig reagent **32** led to α,β-unsaturated ester **33** in 85% yield. After simultaneous reduction of the double bond and cleavage of the benzyl ester by catalytic hydrogenation, 6-methylheptanoic acid (**26**) could be obtained in 92% yield for the final step (Scheme 5).

**Scheme 6:**
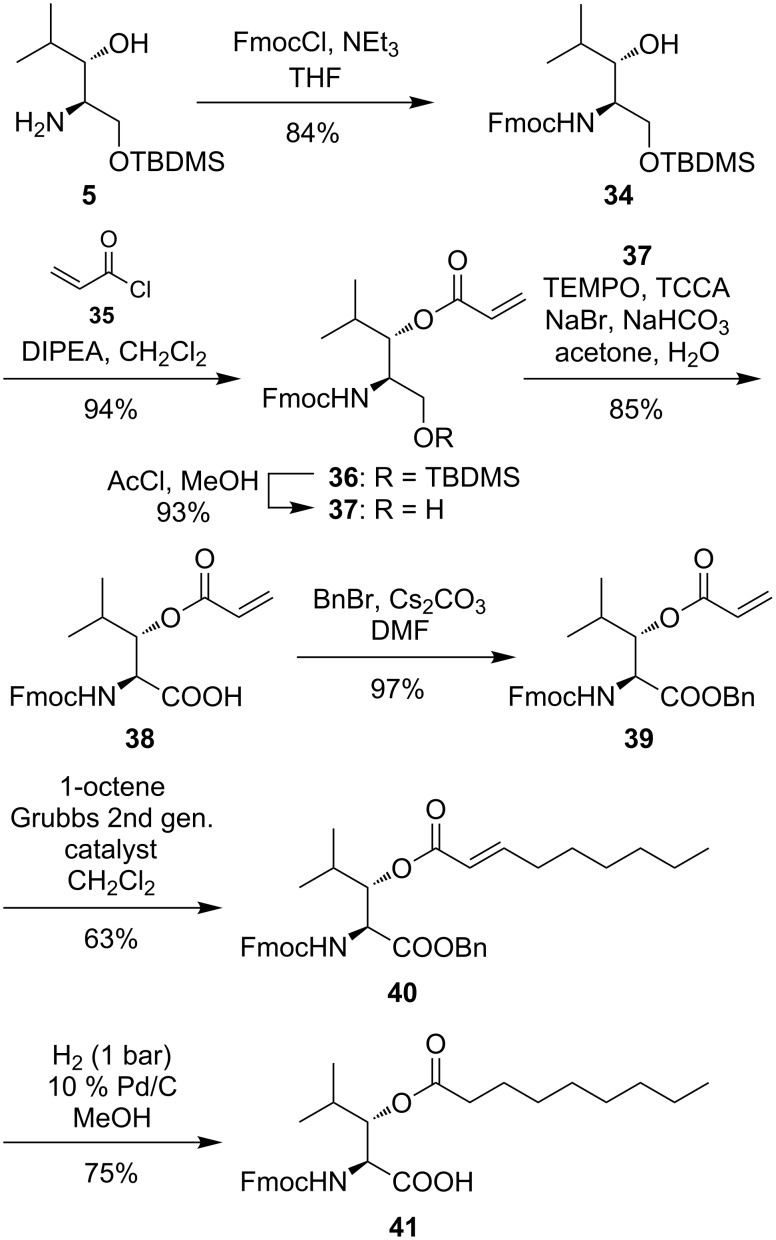
Synthesis of Fmoc-protected building blocks **38** and **41** suitable for SPPS, with late-stage side chain diversification by olefin metathesis.

With respect to the (2*S*,3*S*)-3-hydroxyleucine motif occurring in several different peptidic natural products (vide supra), we also investigated the synthesis of *N*-Fmoc-protected building blocks potentially suitable for solid-phase peptide synthesis (SPPS). It is well established that *O*-acylated β-hydroxy-α-amino acids can be used in Fmoc-strategy peptide syntheses without migration of the acyl unit [37–39]. Based on these findings, we have desired to develop a method for the modification of the acyl side chain of a previously incorporated *O*-acylated β-hydroxy-α-amino acid building block at a very late stage and under mild conditions, e.g., by olefin cross metathesis. Therefore, amino alcohol **5** was Fmoc-protected (product **34**, 84% yield), followed by esterification of the 3-hydroxy group with acryloyl chloride (**35**), thus furnishing acrylate **36** in 94% yield (Scheme 6). After acidic cleavage of the silyl ether in 93% yield [40], the resultant primary alcohol **37** was oxidized using catalytic amounts of TEMPO and trichlorocyanuric acid (TCCA) as stoichiometric oxidant to provide (2*S*,3*S*)-3-hydroxyleucine derivative **38**, a potential building block for SPPS and post-synthetic modification of the peptide, in 85% yield.

In order to demonstrate the feasibility of the olefin cross metathesis approach for the late-stage diversification of the acyl side chain, acid **38** was transformed into benzyl ester **39** in 97% yield (Scheme 6). Subsequent treatment with commercially available 1-octene and Grubbs 2^nd^ generation catalyst [41] afforded lipidated (2*S*,3*S*)-3-hydroxyleucine derivative **40** in 63% yield without any observed homo-coupling of the amino acid. Amino acid **40** was then debenzylated under standard hydrogenation conditions with concomitant reduction of the double bond, thus leading to lipidated amino acid building block **41** suitable for SPPS in 75% yield for the final step.

## Conclusion

In summary, we have developed a divergent approach for the synthesis of several (2*S*,3*S*)-3-hydroxyleucine building blocks employing stereoisomerically pure amino alcohol **5** [32]. Applying different protecting group strategies, we were able to prepare (2*S*,3*S*)-3-hydroxyleucine derivatives suitable for further modification both at the carboxy and the amino moiety, as well as for solid-phase peptide synthesis (SPPS). Furthermore, we have employed such building blocks for the synthesis of protected analogues **15a**,**b** of the tripeptide unit of naturally occurring muraymycin nucleoside antibiotics.

We have also established unprecedented protocols for early- and late-stage derivatizations of the 3-hydroxy group of the (2*S*,3*S*)-3-hydroxyleucine scaffold by esterification of the alcohol or cross metathesis of the corresponding acryloyl ester, respectively. This led to an efficient and versatile access towards acylated (2*S*,3*S*)-3-hydroxyleucine derivatives, thus enabling the preparation of according natural products and analogues thereof. As a first proof-of-principle experiment, the lipidated (2*S*,3*S*)-3-hydroxyleucine subunit of antibacterially active muraymycins B6 and B7 was synthesized in protected form. Overall, our results thus contribute to the methodology for the synthesis of unusual non-proteinogenic amino acid motifs for synthetic natural product chemistry.

## Supporting Information

The Supporting Information features the preparation, analytical data and copies of ^1^H and ^13^C NMR spectra of compounds **6**–**9**, **11**–**13**, **15**–**19**, **21**–**24**, **26**–**28**, **30**, **31**, **33**, **34**, **36**–**41** and racemic HPLC reference **S1** as well as crystallographic data for compound **6**.

File 1 Crystallographic data for compound **6**.

File 2 Experimental procedures and NMR spectra of compounds **6**–**9**, **11**–**13**, **15**–**19**, **21**–**24**, **26**–**28**, **30**, **31**, **33**, **34**, **36**–**41** and **S1**.
