# Supplementary material for: Amino acid motifs in natural products: synthesis of O-acylated derivatives of (2S,3S)-3-hydroxyleucine
Source: Beilstein J Org Chem. 2014 May 16;10:1135–42. doi: 10.3762/bjoc.10.113 (PMC4077382; doi:10.3762/bjoc.10.113)
Supplement: File 2 — Experimental procedures and NMR spectra of compounds 6–9, 11–13, 15–19, 21–24, 26–28, 30, 31, 33, 34, 36–41 and S1. [file Beilstein_J_Org_Chem-10-1135-s002.pdf]

Supporting Information

for

Amino acid motifs in natural products: synthesis of *O*-acylated derivatives of (2*S*,3*S*)-3-hydroxyleucine

Oliver Ries[1], Martin Büschleb[1], Markus Granitzka[2], Dietmar Stalke[2] and Christian Ducho*[1,3]

Address: [1]Department of Chemistry, Institute of Organic and Biomolecular Chemistry, Georg-August-University Göttingen, Tammannstr. 2, 37 077 Göttingen, Germany, [2]Department of Chemistry, Institute of Inorganic Chemistry, Georg-August-University Göttingen, Tammannstr. 4, 37 077 Göttingen, Germany and [3]Department of Pharmacy, Pharmaceutical and Medicinal Chemistry, Saarland University, Campus C2 3, 66 123 Saarbrücken, Germany

Email: Christian Ducho - christian.ducho@uni-saarland.de

*Corresponding author

Experimental procedures and NMR spectra of compounds 6–9, 11–13, 15–19, 21–24, 26–28, 30, 31, 33, 34, 36–41 and S1

Table of contents

Syntheses of novel compounds... S2

Synthesis of racemic HPLC reference **S1** and HPLC analysis of **3**..................... S29

Determination of the X-ray crystal structure of compound **6**............................ S32

^{1}H and ^{13}C NMR spectra of novel compounds.. S33

References.. S65

Syntheses of novel compounds

General methods. All chemicals were purchased from standard suppliers. Reactions involving oxygen and/or moisture sensitive reagents were carried out under an atmosphere of argon using anhydrous solvents. Anhydrous solvents were obtained in the following manner: THF was dried over sodium/benzophenone and distilled, CH_2Cl_2 was dried over P_2O_5 and distilled, MeOH was dried over activated molecular sieves (3 Å) and degassed, DMF was dried over activated molecular sieves (4 Å) and degassed. All other solvents were of technical quality and distilled prior to their use, and deionized water was used throughout. Column chromatography was carried out on silica gel 60 (0.040–0.063 mm, 230–400 mesh ASTM, VWR) under flash conditions. Size exclusion chromatography was carried out on a Japan Analytical Industry Co. LC-9101 Recycling Preparative HPLC equipped with a UV detector 310, a refraction index detector RI-7s, a Jaigel-2H and a Jaigel-2.5H column. Method: eluent: chloroform; flow: 3.5 mL/min; detector wavelength: 254 nm. TLC was performed on aluminum plates precoated with silica gel 60 F_{254} (VWR). Visualization of the spots was carried out using UV light (254 nm) and/or staining under heating (H_2SO_4 staining solution: 4 g vanillin, 25 mL conc. H_2SO_4, 80 mL AcOH and 680 mL MeOH; $KMnO_4$ staining solution: 1 g $KMnO_4$, 6 g K_2CO_3 and 1.5 mL 1.25 M NaOH solution, all dissolved in 100 mL H_2O; ninhydrin staining solution: 0.3 g ninhydrin, 3 mL AcOH and 100 mL 1-butanol). 300 MHz and 600 MHz ^1H and 75 MHz, 76 MHz, 126 MHz and 151 MHz ^{13}C NMR spectra were recorded on Varian MERCURY 300, UNITY 300, INOVA 500 and INOVA 600 spectrometers. All ^{13}C NMR spectra are H-decoupled. All spectra were recorded at room temperature except where indicated otherwise and were referenced internally to solvent reference frequencies wherever possible. Chemical shifts (δ) are quoted in ppm, and coupling constants (*J*) are reported in Hz. Assignment of signals was carried out using H,H-COSY,

HSQC and HMBC spectra obtained on the spectrometers mentioned above. Low-resolution ESI mass spectrometry was performed on a Varian MAT 311 A spectrometer operating in positive ionization mode. High-resolution (HR) ESI mass spectrometry was carried out on a Bruker microTOF spectrometer or a Bruker 7 T FTICR APEX IV spectrometer. Melting points (mp) were measured on a Büchi instrument and are not corrected. Optical rotations were recorded on a Perkin-Elmer polarimeter 241 with a Na source using a 10 cm cell (concentrations in g/100 mL). Infrared spectroscopy (IR) was performed on a Jasco FT/IR-4100 spectrometer equipped with an integrated ATR unit (GladiATR™, PIKE Technologies). Wavenumbers (ν) are quoted in cm. UV spectroscopy was carried out on a Perkin-Elmer Lambda 2 Jasco V-630 spectrometer. Wavelengths of maximum absorption (λ_{max}) are reported in nm with the corresponding logarithmic molar extinction coefficient given in parenthesis (log ε, $\varepsilon/dm^3\,mol^{-1}\,cm^{-1}$).

(2*R*,3*S*)-2-(Dibenzylamino)-1-*O*-(*tert*-butyldimethylsilyl)-3-*O*-levulinyl-4-methyl-pentane-1,3-diol (6)

A solution of levulinic acid (0.21 mL, 2.1 mmol, 2.9 equiv) and DMAP (255 mg, 2.08 mmol, 3.0 equiv) in dry CH_2Cl_2 (7.5 mL) was stirred for 10 min at 0 °C. After dropwise addition of diisopropylcarbodiimide (0.32 mL, 2.1 mmol, 3.0 equiv), stirring was continued for 5 min. A solution of amino alcohol **4** (298 mg, 0.697 mmol, 1.0 equiv) in dry CH_2Cl_2 (7.5 mL) was then added dropwise. The mixture was stirred for 14 h and was allowed to warm to room temperature within this time. Subsequent filtration through a short pad of silica (EtOAc), evaporation of the solvent under reduced pressure and column chromatography (SiO_2, petroleum ether/EtOAc 10:1) of the crude product yielded 303 mg (0.576 mmol, 82% of **6** as a pale yellow solid. TLC R_f 0.29 (petroleum ether/EtOAc 6:1); mp 67 °C; ^1H NMR (300 MHz, C_6D_6): 0.29 (s, 3H, Si(C$\underline{H}_3$)$_2$), 0.30 (s, 3H, Si(C$\underline{H}_3$)$_2$C(CH$_3$)$_3$), 0.73 (d, 3H,

J = 6.7 Hz, 5-H_3), 0.98 (d, 3H, J = 6.7 Hz, 5'-H_3), 1.15 (s, 9H, SiC(C$\underline{H}_3$)$_3$), 1.68 (s, 3H, Lev-5-H_3), 2.15 (ddd, 1H, J = 17.9, 7.0, 5.1 Hz, Lev-3-H_a), 2.24-2.56 (m, 4H, Lev-2-H_2, Lev-3-H_b, 4-H), 3.33 (ddd, 1H, J = 7.8, 7.4, 4.1 Hz, 2-H), 3.97 (d, 2H, J = 13.6 Hz, C$\underline{H}_a$H$_b$Ph), 4.06 (d, 2H, J = 13.6 Hz, CH$_a\underline{H}_b$Ph), 4.12 (dd, 1H, J = 10.9, 7.4 Hz, 1-H_a), 4.18 (dd, 1H, J = 10.9, 4.1 Hz, 1-H_b), 5.44 (dd, 1H, J = 7.8, 3.7 Hz, 3-H), 7.13-7.33 (m, 6H, Ph-H), 7.50-7.59 (m, 4H, Ph-H); ^{13}C NMR (76 MHz, C_6D_6): -5.4, -5.4, 15.8, 18.4, 20.4, 26.2, 28.1, 29.1, 29.2, 37.5, 55.5, 58.7, 61.3, 76.6, 127.2, 127.9, 128.5, 129.6, 140.7, 172.2, 204.6; IR (ATR) ν 1718, 1361, 1215, 1156, 1115, 1080, 973, 856, 840, 775, 745, 697; $[\alpha]_D^{20}$ -18.1 (c 1.5, $CHCl_3$); MS (ESI^+) *m/z* 526.3 ($M+H^+$); HRMS (ESI^+) *m/z* calcd for $C_{31}H_{48}NO_4Si$ 526.3347 ($M+H^+$), found 526.3342.

(2*R*,3*S*)-2-(*tert*-Butyloxycarbonylamino)-1-*O*-(*tert*-butyldimethylsilyl)-4-methyl-pentane-1,3-diol (7)

A solution of **5** (500 mg, 2.02 mmol, 1.0 equiv) in a mixture of THF (4 mL) and aq. sat. $NaHCO_3$ solution (1 mL) was treated with di-*tert*-butyldicarbonate (885 mg, 4.05 mmol, 2.0 equiv) at 0 °C. The reaction mixture was stirred for 19 h and was allowed to reach room temperature within this time. The reaction was quenched with water (10 mL) and the aqueous layer was extracted with ethyl acetate (3 × 10 mL). The combined organics were dried over Na_2SO_4 and the solvent was removed under reduced pressure. The resultant crude product was purified by column chromatography (petroleum ether/EtOAc 9:1) to yield 554 mg (1.59 mmol, 79%) of **7** as a pale yellow oil. TLC R_f 0.17 (petroleum ether/EtOAc 9:1); ^{1}H NMR (300 MHz, C_6D_6): -0.03 (s, 3H, Si(C$\underline{H}_3$)$_2$(C(CH$_3$)$_3$)), 0.01 (s, 3H, Si(C$\underline{H}_3$)$_2$(C(CH$_3$)$_3$)), 0.76 (d, 3H, J = 6.7 Hz, 5-H_3), 0.86 (s, 9H, Si(CH$_3$)$_2$(C(C$\underline{H}_3$)$_3$)), 1.07 (d, 3H, J = 6.6 Hz, 5-H_3), 1.47 (s, 9H, NCO$_2$C(C$\underline{H}_3$)$_3$), 1.65-1.80 (m, 1H, 4-H), 2.77 (d, 1H, J = 7.5 Hz, OH), 3.21-3.33 (m, 1H, 3-H), 3.76-3.93 (m, 3H, 1-H_2, 2-H), 5.30 (d, 1H, J = 6.9 Hz, NH); ^{13}C

NMR (126 MHz, C_6D_6): -5.7, -5.6, 18.3, 18.4, 19.4, 25.9, 28.5, 31.7, 52.5, 63.8, 78.9, 78.9, 155.6; IR (ATR) ν 3448, 2929, 1697, 1498, 1471, 1365, 1252, 1168, 1099, 1069, 1005, 835, 776; $[\alpha]_D^{20}$ -19.1 (c 1.1, MeOH); MS (ESI^+) *m/z* 348.2 ($M+H^+$), 370.2 ($M+Na^+$); HRMS (ESI^+) *m/z* calcd for $C_{17}H_{37}NNaO_4Si$ 370.2384 ($M+Na^+$), found 370.2386.

(4*R*,5*S*)-*N*-(*tert*-Butyloxycarbonyl)-4-(*tert*-butyldimethylsilyloxymethyl)-5-isopropyl-2,2-dimethyloxazolidine (8)

A suspension of **7** (50 mg, 0.14 mmol, 1.0 equiv), *rac*-camphorsulfonic acid (5 mg, 0.02 mmol, 15 mol %) and $MgSO_4$ (60 mg) in 2,2-dimethoxypropane (1 mL) was stirred for 24 h at 50 °C under argon atmosphere. The reaction was quenched with aq. sat. $NaHCO_3$ solution (3 mL) and the aqueous layer was extracted with EtOAc (3 × 3 mL). The combined organics were dried over Na_2SO_4 and the solvent was removed under reduced pressure. The resultant crude product was purified by column chromatography (SiO_2, petroleum ether/EtOAc 12:1) yielding 52 mg (0.13 mmol, 93%) of **8** as a clear oil. TLC R_f 0.52 (petroleum ether/EtOAc 9:1); ^1H NMR (300 MHz, DMSO-d_6, 100 °C): 0.07 (s, 3H, $Si(C\underline{H}_3)_2C(CH_3)_3$), 0.08 (s, 3H, $Si(C\underline{H}_3)_2C(CH_3)_3$), 0.90 (s, 9H, $Si(CH_3)_2C(C\underline{H}_3)_3$), 0.96 (d, 3H, *J* = 6.5 Hz, $CH(C\underline{H}_3)_2$), 0.98 (d, 3H, *J* = 6.5 Hz, $CH(C\underline{H}_3)_2$), 1.43-1.47 (m, 15H, $NCO_2C(CH_3)_3$, 2 × 2-CH_3), 1.99 (dqq, 1H, *J* = 9.9, 6.5, 6.5 Hz, $C\underline{H}(CH_3)_2$), 3.53 (dd, 1H, *J* = 9.9, 2.5 Hz, 4-$C\underline{H}_aH_b$), 3.62 (dd, 1H, *J* = 9.9, 4.5 Hz, 5-H), 3.75 (dd, 1H, *J* = 9.9, 7.8 Hz, 4-$CH_a\underline{H}_b$), 3.80-3.88 (m, 1H, 4-H); ^{13}C NMR (76 MHz, DMSO-d_6, 100 °C): -6.2, -6.2, 17.3, 19.0, 19.1, 25.2, 26.0, 27.6, 59.3, 60.1, 78.5, 81.3, 90.9, 150.5; IR (ATR) ν 2960, 1703, 1382, 1257, 1088, 1051, 1012, 794; $[\alpha]_D^{20}$ -9.2 (c 0.60, MeOH); MS (ESI^+) *m/z* 388.3 ($M+H^+$), 410.3 ($M+Na^+$); HRMS (ESI^+) *m/z* calcd for $C_{20}H_{41}NNaO_4Si$ 410.2697 ($M+Na^+$), found 410.2699.

(4*R*,5*S*)-*N*-(*tert*-Butyloxycarbonyl)-4-hydroxymethyl-5-isopropyl-2,2-dimethyl-oxazolidine (9)

A solution of **8** (49 mg, 0.13 mmol, 1.0 equiv) and TBAF trihydrate (60 mg, 0.19 mmol, 1.5 equiv) in THF (1 mL) was stirred at room temperature for 3 h. The solvent was removed under reduced pressure and the resultant residue was purified by column chromatography (SiO_2, petroleum ether/EtOAc 6:1) to yield 29 mg (0.11 mmol, 80%) of **9** as a white solid. TLC R_f 0.15 (petroleum ether/EtOAc 6:1); mp 65 °C; ^1H NMR (300 MHz, DMSO-d_6, 100 °C): 0.96 (d, 3H, J = 6.5 Hz, $CH(C\underline{H}_3)_2$), 0.97 (d, 3H, J = 6.5 Hz, $CH(C\underline{H}_3)_2$), 1.44 (s, 9H, $NCO_2C(CH_3)_3$), 1.46 (s, 6H, 2 × 2-CH_3), 2.01 (dqq, 1H, J = 9.6, 6.5, 6.5 Hz, $C\underline{H}(CH_3)_2$), 3.42 (dd, 1H, J = 10.8, 3.5 Hz, 4-$C\underline{H}_aH_b$), 3.60 (dd, 1H, J = 10.8, 7.8 Hz, 4-$CH_a\underline{H}_b$), 3.61 (dd, 1H, J = 9.6, 4.7 Hz, 5-H), 3.79-3.88 (m, 1H, 4-H); ^{13}C NMR (76 MHz, DMSO-d_6, 100 °C): 18.8, 19.3, 23.4, 23.5, 26.0, 27.6, 58.9, 59.5, 78.4, 81.4, 90.9, 150.9; IR (ATR) ν 3402, 2962, 1692, 1660, 1405, 1389, 1363, 1173, 1128, 1065, 1042; $[\alpha]_D^{20}$ -4.6 (c 0.52, MeCN); MS (ESI^+) *m/z* 274.2 ($M+H^+$), 296.2 ($M+Na^+$); HRMS (ESI^+) *m/z* calcd for $C_{14}H_{27}NNaO_4$ 296.1832 ($M+Na^+$), found 296.1840.

(4*S*,5*S*)-*N*-(*tert*-Butyloxycarbonyl)-5-isopropyl-2,2-dimethyloxazolidine-4-carboxylic acid (11)

Ruthenium(III) chloride (16 mg, 77 µmol, 0.1 equiv) was added to a solution of **9** (200 mg, 0.732 mmol, 1.0 equiv) and sodium periodate (545 mg, 2.55 mmol, 3.5 equiv) in a mixture of MeCN, CCl_4 and water (16 mL, 2:1:1) at 0 °C, and stirring was continued at this temperature for 4 h. The reaction mixture was acidified with hydrochloric acid (1 M, 10 mL) and the aqueous layer was extracted with EtOAc (3 × 20 mL). The combined organics were dried over Na_2SO_4 and the solvent was removed under reduced pressure. The resultant residue was

purified by column chromatography (SiO_2, petroleum ether/EtOAc 1:1) yielding 172 mg (0.599 mmol, 82%) of **11** as a clear oil. TLC R_f 0.27 (petroleum ether/EtOAc 1:1); ^1H NMR (300 MHz, DMSO-d_6, 100 °C): 0.97 (d, 3H, J = 6.6 Hz, CH(C$\underline{H}_3$)$_2$), 0.99 (d, 3H, J = 6.6 Hz, CH(C$\underline{H}_3$)$_2$), 1.41 (s, 9H, $NCO_2C(CH_3)_3$), 1.46 (s, 3H, 2-CH_3), 1.60 (s, 3H, 2-CH_3), 1.70 (dqq, 1H, J = 9.3, 6.6, 6.6 Hz, C$\underline{H}$(CH_3)$_2$), 3.82 (dd, 1H, J = 9.3, 6.0 Hz, 5-H), 4.24 (d, 1H, J = 6.0 Hz, 4-H); ^{13}C NMR (151 MHz, DMSO-d_6, 100 °C): 18.4, 18.7, 23.6, 24.7, 27.6, 27.9, 61.5, 78.7, 80.8, 92.5, 150.3, 170.6; IR (ATR) ν 3141, 2973, 1747, 1709, 1670, 1417, 1365, 1183, 1162, 1127, 1091, 884; $[\alpha]_D^{20}$ -5.3 (c 0.42, MeOH); MS (ESI$^+$) *m/z* 310.2 (M+Na$^+$); HRMS (ESI$^+$) *m/z* calcd for $C_{14}H_{25}NNaO_5$ 310.1625 (M+Na$^+$), found 310.1624.

2-(Trimethylsilyl)-ethyl (4*S*,5*S*)-*N*-(*tert*-butyloxycarbonyl)-5-isopropyl-2,2-dimethyloxazolidine-4-carboxylate (12a)

Diisopropylcarbodiimide (20 µL, 0.13 mmol, 1.8 equiv) was added to a solution of **11** (20 mg, 70 µL, 1.0 equiv), 2-(trimethylsilyl)ethanol (50 µL, 0.35 mmol, 5.0 equiv) and DMAP (13 mg, 0.11 mmol, 1.5 equiv) in dry THF at room temperature under argon atmosphere. Stirring was continued for 15 h and the reaction was quenched with hydrochloric acid (0.5 M, 5 mL). The aqueous layer was extracted with Et_2O (3 × 10 mL) and the combined organics were dried over Na_2SO_4 . The solvent was removed under reduced pressure and the resultant residue was purified by column chromatography (SiO_2, petroleum ether/EtOAc 19:1) to yield 20 mg (52 µmol, 74%) of **12a** as a clear oil. TLC R_f 0.28 (petroleum ether/EtOAc 20:1); ^1H NMR (300 MHz, DMSO-d_6, 100 °C): 0.06 (s, 9H, $Si(CH_3)_3$), 0.96 (d, 3H, J = 6.5 Hz, CH(C$\underline{H}_3$)$_2$), 0.98 (d, 3H, J = 6.6 Hz, CH(C$\underline{H}_3$)$_2$), 0.99 (t, 2H, J = 8.5 Hz, 2'-H_2), 1.40 (bs, 9H, $NCO_2C(CH_3)_3$), 1.47 (s, 3H, 2-CH_3), 1.52-1.68 (m, 1H, C$\underline{H}$(CH_3)$_2$), 1.63 (s, 3H, 2-CH_3), 3.86 (dd, 1H, J = 9.4, 5.9 Hz, 5-H), 4.18 (t, 2H, J = 8.5 Hz, 1'-H_2), 4.31 (d, 1H, J = 5.9 Hz, 4-H); ^{13}C NMR (151 MHz, DMSO-d_6, 100 °C): -2.2, 16.7, 18.3, 18.7, 23.6, 24.6,

27.5, 27.9, 61.5, 62.0, 78.9, 80.9, 92.7, 150.1, 169.3; IR (ATR) ν 1708, 1365, 1248, 1162, 1126, 1088, 1052, 1026, 884, 737; $[\alpha]_D^{20}$ -2.3 (c 0.19, $CHCl_3$); MS (ESI^+) *m/z* 388.3 ($M+H^+$), 410.3 ($M+Na^+$); HRMS (ESI^+) *m/z* calcd for $C_{19}H_{37}NNaO_5Si$ 410.2333 ($M+Na^+$), found 410.2333.

Benzyl (4*S*,5*S*)-*N*-(*tert*-butyloxycarbonyl)-5-isopropyl-2,2-dimethyloxazolidine-4-carboxylate (12b)

A solution of **11** (235 mg, 0.819 mmol, 1.0 equiv) and caesium carbonate (803 mg, 2.46 mmol, 3.0 equiv) in dry DMF (12 mL) was treated at room temperature under an atmosphere of argon with benzyl bromide (0.97 mL, 8.2 mmol, 9.9 equiv) and stirred for 20 h. The reaction mixture was diluted with water (50 mL) and the aqueous layer was extracted with EtOAc (3 × 50 mL). The combined organics were dried over $MgSO_4$ and the solvent was removed under reduced pressure. The resultant residue was purified by column chromatography (SiO_2, petroleum ether/EtOAc 19:1) and size exclusion chromatography yielding 252 mg (0.668 mmol, 82%) of **12b** as a clear oil. TLC R_f 0.34 (petroleum ether/EtOAc 19:1); ^1H NMR (300 MHz, DMSO-d_6, 120 °C): 0.93 (d, 3H, *J* = 6.6 Hz, $CH(C\underline{H}_3)_2$), 0.94 (d, 3H, *J* = 6.5 Hz, $CH(C\underline{H}_3)_2$), 1.37 (bs, 9H, $NCO_2C(CH_3)_3$), 1.48 (s, 3H, 2-CH_3), 1.51-1.66 (m, 1H, $C\underline{H}(CH_3)_2$), 1.61 (s, 3H, 2-CH_3), 3.87 (dd, 1H, *J* = 9.3, 5.9 Hz, 5-H), 4.41 (d, 1H, *J* = 5.9 Hz, 4-H), 5.11-5.22 (m, 2H, CH_2Ph), 7.30-7.42 (m, 5H, Ph-H); ^{13}C NMR (75 MHz, DMSO-d_6, 120 °C): 18.1, 18.4, 23.7, 24.7, 27.4, 27.7, 61.5, 65.6, 79.1, 80.7, 92.6, 127.2, 127.4, 127.6, 127.8, 135.1, 169.0, $N\underline{C}O_2C(CH_3)_3$ not detectable; IR (ATR) ν 1747, 1706, 1375, 1365, 1262, 1216, 1161, 1125, 1086, 1052, 1028, 882, 697; $[\alpha]_D^{20}$ -19.3 (c 1.5, $CHCl_3$); UV (MeCN) λ_{max} (log ε) 205 (4.06), 252 (2.46), 257 (2.51), 262 (2.44); MS (ESI^+) *m/z* 378.2 ($M+H^+$), 400.2 ($M+Na^+$); HRMS (ESI^+) *m/z* calcd for $C_{21}H_{31}NNaO_5$ 400.2094 ($M+Na^+$), found 400.2093.

***t*-BuO-L-valine-urea-L-epicapreomycidine(*N*⁷-Pbf)-((3*S*)-3-hydroxy-L-leucine)-OTMSE tripeptide (15a)**

A solution of **12a** (34 mg, 88 µmol) in CH_2Cl_2/TFA (9:1, 10 mL) was stirred at room temperature for 20 h. The reaction mixture was diluted with toluene (5 mL) and the solvent was removed under reduced pressure. The resultant crude hydroxyleucine TMSE ester trifluoroacetic acid salt **13a** was dried in vacuo and used in the subsequent peptide coupling step without further purification. To a solution of **14** (7.8 mg, 13 µmol, 1.0 equiv), **13a** (4.5 mg, 13 µmol, 1.0 equiv), HOBt (2.5 mg, 19 µmol, 1.5 equiv) and DIPEA (2.1 µL, 13 µmol, 1.0 equiv) in dry DMF (1.8 mL) at 0 °C under argon atmosphere, EDC hydrochloride (2.4 mg, 13 µmol, 1.0 equiv) was added. The reaction mixture was stirred at room temperature for 11 h. Silica was added and the solvent was removed under reduced pressure followed by column chromatography (SiO_2, CH_2Cl_2/MeOH 98.5:1.5) yielding 5.4 mg (6.3 µmol, 51% over 2 steps from **12a**) of **15a** as a clear oil. TLC R_f 0.32 (CH_2Cl_2/MeOH 19:1); ^1H NMR (600 MHz, $CDCl_3$, 50 °C, rotamers): 0.01 (s, 9H, $Si(CH_3)_3$), 0.83 (d, 3H, *J* = 6.9 Hz, Val-4-H_3), 0.92 (d, 3H, *J* = 6.9 Hz, Val-4-H_3), 0.94 (d, 3H, *J* = 6.6 Hz, HyLeu-5-H_3), 0.99 (d, 3H, *J* = 6.7 Hz, HyLeu-5-H_3), 0.97-1.01 (m, 2H, TMSE-CH_2Si), 1.45 (s, 9H, $C(CH_3)_3$), 1.47 (s, 6H, Pbf-2-CH_3), 1.85-1.94 (m, 3H, HyLeu-4-CH, Epicap-4-H_2), 2.06 (s, 3H, Pbf-7-CH_3), 2.11-2.19 (m, 1H, Val-3-H), 2.48 (s, 3H, Pbf-6-CH_3), 2.57 (s, 3H, Pbf-4-CH_3), 2.93 (s, 2H, Pbf-3-H_2), 3.22-3.29 (m, 1H, Epicap-5-H_a), 3.36 (d, 1H, *J* = 8.8 Hz, HyLeu-3-H), 3.40-3.47 (m, 1H, Epicap-5-H_b), 4.07-4.14 (m, 1H, Epicap-3-H), 4.19 (dd, 2H, *J* = 8.9, 8.8 Hz, TMSE-CH_2O), 4.25 (dd, 0.5H, *J* = 8.8, 4.3 Hz, Val-2-H), 4.36-4.46 (m, 1H, Epicap-2-H), 4.53 (br s, 1H, OH), 4.58 (dd, 0.5H, *J* = 7.5, 7.0 Hz, Val-2-H), 4.72 (dd, 1H, *J* = 8.2, 2.3 Hz, HyLeu-2-H), 5.61 (s, 0.5H, Val-2-NH), 5.73 (d, 0.5H, *J* = 7.3 Hz, Val-2-NH), 6.45 (s, 1H, Epicap-2-NH), 6.82 (s, 0.5H, Epicap-N^6-H), 6.97 (s, 0.5H, Epicap-N^6-H), 7.44 (s, 1H, Epicap-N^8-H), 7.51 (s, 1H, HyLeu-2-NH); ^{13}C NMR (126 MHz, $CDCl_3$,

50 °C, rotamers: -1.6, 12.4, 17.5, 17.8, 18.1, 18.8, 19.0, 19.2, 19.4, 19.5, 21.7, 28.2, 28.6, 30.8, 31.5, 38.0, 43.4, 50.8, 55.5, 55.6, 58.6, 63.5, 78.9, 81.8, 86.2, 117.4, 124.4, 132.2, 132.9, 138.4, 153.8, 157.2, 158.7, 169.4, 170.7; IR (ATR) ν 2972, 1735, 1610, 1541, 1366, 1250, 1151, 1087, 651, 561; UV (MeOH) λ_{max} (log ε) 253 (4.80), 282 (4.76); $[\alpha]_D^{22}$ +34.2 (c 0.17, $CHCl_3$); MS (ESI^+) *m/z* 875.4 ($M+Na^+$); HRMS (ESI^-) *m/z* calcd for $C_{40}H_{67}N_6O_{10}SSi$ 851.4414 ($M-H^+$), found 851.4421.

***t*-BuO-L-valine-urea-L-epicapreomycidine(*N*^{7}-Pbf)-((3*S*)-3-hydroxy-L-leucine)-OBn tripeptide (15b)**

A solution of **12b** (256 mg, 0.678 mmol) in CH_2Cl_2/TFA (9:1, 25 mL) was stirred at room temperature for 19 h. The reaction mixture was diluted with toluene (10 mL) and the solvent was removed under reduced pressure. The resultant crude hydroxyleucine TMSE ester trifluoroacetic acid salt **13b** was dried in vacuo and used in the subsequent peptide coupling step without further purification. To a solution of **14** (157 mg, 0.252 mmol, 1.0 equiv) in dry DMF (5.5 mL), HOBt (37 mg, 0.28 mmol, 1.1 equiv), PyBOP (131 mg, 0.252 mmol, 1.0 equiv) and DIPEA (94 µL, 0.55 mmol, 2.2 equiv) were added at room temperature under argon atmosphere. The solution was stirred for 10 min and subsequently treated with a solution of **13b** (84 mg, 0.25 mmol, 1.0 equiv) and DIPEA (47 µL, 0.28 mmol, 1.1 equiv) in dry DMF (2.5 mL). The reaction mixture was stirred at room temperature for 23 h. The solvent was removed under reduced pressure and the resultant residue was purified by column chromatography (SiO_2, CH_2Cl_2/MeOH gradient (2–3%)) to yield 186 mg (0.22 mmol, 88% over 2 steps from **12b**) of **15b** as a white foam with minor unseparable impurities. TLC R_f 0.40 (CH_2Cl_2/MeOH 19:1); ^1H NMR (300 MHz, CD_3OD): 0.89 (d, 3H, J = 6.9 Hz, Val-4-H_3), 0.90 (d, 3H, J = 7.0 Hz, Val-4-H_3), 0.93 (d, 3H, J = 6.9 Hz, HyLeu-5-H_3), 0.95 (d, 3H, J = 6.4 Hz, HyLeu-5-H_3), 1.42 (s, 6H, Pbf-2-CH_3), 1.45 (s, 9H, $C(CH_3)_3$), 1.72-1.90 (m, 3H,

Epicap-4-H_2, HyLeu-4-H), 2.04 (s, 3H, Pbf-7-CH_3), 2.02-2.15 (m, 1H, Val-3-H), 2.47 (s, 3H, Pbf-6-CH_3), 2.53 (s, 3H, Pbf-4-CH_3), 2.95 (s, 2H, Pbf-3-H_2), 3.07-3.17 (m, 1H, Epicap-5-H_a), 3.24-3.34 (m, 1H, Epicap-5-H_b), 3.54 (dd, 1H, *J* = 6.2, 6.2 Hz, HyLeu-3-H), 3.60 (dt, 1H, *J* = 7.4, 6.0 Hz, Epicap-3-H), 4.06 (d, 1H, *J* = 4.9 Hz, Val-2-H), 4.39 (t, 1H, *J* = 7.6 Hz, Epicap-2-H), 4.65 (d, 1H, *J* = 6.0 Hz, HyLeu-2-H), 5.12 (s, 2H, CH_2Ph), 7.25-7.36 (m, 5H, Ph-H); ^{13}C NMR (126 MHz, CD_3OD): 12.6, 18.0, 18.4, 19.7, 20.0, 22.7, 28.4, 28.7, 31.5, 32.2, 38.2, 44.0, 52.7, 56.8, 57.0, 60.1, 68.0, 77.8, 82.6, 87.5, 118.2, 125.8, 129.1, 129.3, 129.4, 133.5, 134.5, 137.0, 139.4, 154.9, 159.6, 159.8, 171.5, 171.6, 173.0; MS (ESI$^+$) *m/z* 865.5 (M+Na$^+$); HRMS (ESI$^+$) *m/z* calcd for $C_{42}H_{62}N_6NaO_{10}S$ 865.4140 (M+Na$^+$), found 865.4141.

(2*R*,3*S*)-2-(Benzyloxycarbonylamino)-1-*O*-(*tert*-butyldimethylsilyl)-4-methyl-pentane-1,3-diol (16)

Cbz chloride (1.30 mL, 9.14 mmol, 1.8 equiv) was added at 0 °C to a solution of **5** (1.27 g, 5.13 mmol, 1.0 equiv) and Na_2CO_3 (1.63 g, 15.4 mmol, 3.0 equiv) in water/1,4-dioxane (3:5, 32 mL). The reaction mixture was stirred for 16 h and was allowed to reach room temperature within this time. The reaction was quenched with water (20 mL) and the aqueous layer was extracted with EtOAc (3 × 20 mL). The combined organics were dried over Na_2SO_4 and the solvent was removed under reduced pressure. The resultant residue was purified by column chromatography (SiO_2, petroleum ether/EtOAc 4:1) yielding 1.45 g (3.80 mmol, 74%) of **16** as a clear oil. TLC R_f 0.08 (petroleum ether/EtOAc 9:1); ^{1}H NMR (300 MHz, $CDCl_3$): 0.03 (s, 3H, Si($C\underline{H}_3$)$_2$C(CH_3)$_3$), 0.04 (s, 3H, Si($C\underline{H}_3$)$_2$C(CH_3)$_3$), 0.86 (s, 9H, Si(CH_3)$_2$C($C\underline{H}_3$)$_3$), 0.90 (d, 3H, *J* = 6.8 Hz, 5-H_3), 1.03 (d, 3H, *J* = 6.6 Hz, 5-H_3), 1.66-1.82 (m, 1H, 4-H), 2.95 (d, 1H, *J* = 8.8 Hz, OH), 3.25 (ddd, 1H, *J* = 8.8, 8.8, 4.4 Hz, 3-H), 3.68-3.78 (m, 1H, 2-H), 3.84 (dd, 1H, *J* = 10.5, 2.1 Hz, 1-H_a), 3.98 (dd, 1H, *J* = 10.5, 2.3 Hz, 1-H_b), 5.09 (s, 2H, NCO_2CH_2Ph), 5.61 (d, 1H, *J* = 8.3 Hz, NH), 7.24-7.38 (m, 5H, Ph-H); ^{13}C NMR (126 MHz,

$CDCl_3$): -5.6, -5.6, 18.2, 18.9, 19.3, 25.9, 31.8, 52.0, 63.6, 66.7, 79.5, 128.0, 128.0, 128.4, 136.5, 155.8; IR (ATR) ν 3442, 2954, 1700, 1503, 1470, 1252, 1214, 1098, 1066, 1005, 834, 775, 734, 695; UV (MeCN) λ_{max} (log ε) 206 (6.96), 253 (2.19), 258 (2.28); $[\alpha]_D^{20}$ -26.9 (c 0.52, $CHCl_3$); MS (ESI^+) *m/z* 382.2 ($M+H^+$), 404.2 ($M+Na^+$); HRMS (ESI^+) *m/z* calcd for $C_{20}H_{35}NNaO_4Si$ 404.2228 ($M+Na^+$), found 404.2230.

(4*R*,5*S*)-*N*-(Benzyloxycarbonyl)-4-(*tert*-butyldimethylsilyloxymethyl)-5-isopropyl-2,2-dimethyloxazolidine (17)

A solution of **16** (292 mg, 0.765 mmol, 1.0 equiv), rac. camphorsulfonic acid (27 mg, 0.12 mmol, 15 mol %) and $MgSO_4$ (306 mg, 2.54 mmol, 3.3 equiv) in 2,2-dimethoxypropane (5 mL) was stirred for 27 h at 50 °C under an argon atmosphere. The reaction was quenched with aq. sat. $NaHCO_3$ solution (20 mL) and the aqueous layer was extracted with EtOAc (3 × 20 mL). The combined organics were dried over Na_2SO_4 and the solvent was removed under reduced pressure. The resultant residue was purified by column chromatography (SiO_2, petroleum ether/EtOAc 12:1) yielding 256 mg (0.607 mmol, 79%) of **17** as a clear oil. TLC R_f 0.45 (petroleum ether/EtOAc 12:1); ^1H NMR (300 MHz, DMSO-d_6, 120 °C): 0.01 (s, 6H, $Si(C\underline{H}_3)_2C(CH_3)_3$), 0.85 (s, 9H, $Si(CH_3)_2C(C\underline{H}_3)_3$), 0.95 (d, 3H, *J* = 6.5 Hz, $CH(C\underline{H}_3)_2$), 0.97 (d, 3H, *J* = 6.5 Hz, $CH(C\underline{H}_3)_2$), 1.46 (s, 6H, 2 × 2-CH_3), 1.99 (dqq, 1H, *J* = 10.1, 6.5, 6.5 Hz, $C\underline{H}(CH_3)_2$), 3.54 (dd, 1H, *J* = 10.3, 3.0 Hz, 4-$C\underline{H}_aH_b$), 3.65 (dd, 1H, *J* = 10.1, 4.6 Hz, 5-H), 3.74 (dd, 1H, *J* = 10.3, 7.8 Hz, 4-$CH_a\underline{H}_b$), 3.92 (ddd, 1H, *J* = 7.8, 4.6, 3.0 Hz, 4-H), 5.07 (d, 1H, *J* = 17.3 Hz, $NCO_2C\underline{H}_aH_bPh$), 5.11 (d, 1H, *J* = 17.3 Hz, $NCO_2CH_a\underline{H}_bPh$), 7.26-7.37 (m, 5H, Ph-H); ^{13}C NMR (126 MHz, DMSO-d_6, 35 °C, rotamers): -5.8, -5.7, -5.6, 17.7, 17.8, 19.5, 19.5, 19.8, 19.9, 24.3, 24.3, 25.6, 25.7, 26.5, 26.5, 27.5, 59.2, 59.7, 59.9, 60.6, 65.7, 66.2, 81.3, 81.9, 91.4, 91.6, 127.1, 127.5, 127.7, 127.9, 128.2, 128.2, 136.4, 151.0; IR (ATR) ν 2956, 1705, 1403, 1350, 1255, 1091, 1053, 834, 776, 696; UV (MeCN) λ_{max} (log ε) 253

(2.40), 257 (2.45); MS (ESI^+) *m/z* 422.3 ($M+H^+$), 444.3 ($M+Na^+$); HRMS (ESI^+) *m/z* calcd for $C_{23}H_{40}NO_4Si$ 422.2721 ($M+H^+$), found 422.2718.

(4*R*,5*S*)-*N*-(Benzyloxycarbonyl)-4-(hydroxymethyl)-5-isopropyl-2,2-dimethyloxazolidine (18)

A solution of **17** (1.19 g, 2.82 mmol, 1.0 equiv) and TBAF trihydrate (1.31 g, 4.15 mmol, 1.5 equiv) in THF (25 mL) was stirred at room temperature for 19 h. The solvent was removed under reduced pressure and the resultant residue was purified by column chromatography (SiO_2, petroleum ether/EtOAc 4:1) yielding 821 mg (2.67 mmol, 95%) of **18** as a clear oil. TLC R_f 0.14 (petroleum ether/EtOAc 4:1); ^1H NMR (300 MHz, DMSO-d_6, 100 °C): 0.96 (d, 3H, J = 6.5 Hz, CH(C$\underline{H}_3$)$_2$), 0.98 (d, 3H, J = 6.5 Hz, CH(C$\underline{H}_3$)$_2$), 1.47 (s, 3H, 2-CH_3), 1.48 (s, 3H, 2-CH_3), 2.02 (dqq, 1H, J = 10.0, 6.5, 6.5 Hz, C$\underline{H}$(CH_3)$_2$), 3.39-3.50 (m, 1H, 4-C$\underline{H}_a$$H_b$), 3.57-3.69 (m, 1H, 4-C$H_a$$\underline{H}_b$), 3.64 (dd, J = 10.0, 4.7 Hz, 5-H), 3.88-3.98 (m, 1H, 4-H), 4.25-4.37 (m, 1H, OH), 5.11 (s, 2H, CH_2Ph), 7.26-7.43 (m, 5H, Ph-H); ^{13}C NMR (76 MHz, DMSO-d_6, 100 °C): 18.8, 19.3, 26.0, 58.7, 59.5, 65.4, 81.7, 91.3, 127.0, 127.2, 127.8, 136.5, 151.4; IR (ATR) ν 3456, 2959, 1694, 1406, 1351, 1253, 1128, 1078, 1042, 766, 735, 697; UV (MeCN) λ_{max} (log ε) 252 (2.75), 257 (2.75); $[\alpha]_D^{20}$ +2.6 (c 0.45, $CHCl_3$); MS (ESI^+) *m/z* 308.2 ($M+H^+$), 330.2 ($M+Na^+$); HRMS (ESI^+) *m/z* calcd for $C_{17}H_{26}NO_4$ 308.1856 ($M+H^+$), found 308.1854.

(4*S*,5*S*)-*N*-(Benzyloxycarbonyl)-5-isopropyl-2,2-dimethyloxazolidine-4-carboxylic acid (19)

Ruthenium(III) chloride (57 mg, 0.27 mmol, 0.1 equiv) was added at 0 °C to a solution of **18** (821 mg, 2.67 mmol, 1.0 equiv) and sodium periodate (2.01 g, 9.69 mmol, 3.6 equiv) in a mixture of MeCN, CCl_4 and water (2:1:1, 100 mL) and stirred for 5 h at 0 °C. The reaction

mixture was acidified with hydrochloric acid (1 M, 50 mL) and the aqueous layer was extracted with EtOAc (3 × 100 mL). The combined organics were dried over Na_2SO_4 and the solvent was removed under reduced pressure. The resultant residue was purified by column chromatography (SiO_2, petroleum ether/EtOAc 1:2) yielding 561 mg (1.75 mmol, 65%) of **19** as a clear oil. TLC R_f 0.12 (petroleum ether/EtOAc 2:3); ^{1}H NMR (300 MHz, DMSO-d_6, 100 °C): 0.98 (d, 3H, J = 6.6 Hz, CH(C<u>H</u>$_3$)$_2$), 1.00 (d, 3H, J = 6.6 Hz, CH(C<u>H</u>$_3$)$_2$), 1.49 (s, 3H, 2-CH_3), 1.63 (s, 3H, 2-CH_3), 1.72 (dqq, 1H, J = 9.3, 6.6, 6.6 Hz, C<u>H</u>(CH_3)$_2$), 3.88 (dd, 1H, J = 9.3, 6.0 Hz, 5-H), 4.38 (d, 1H, J = 6.0 Hz, 4-H), 5.00-5.18 (m, 2H, CH_2Ph), 7.25-7.42 (Ph-H); ^{13}C NMR (126 MHz, DMSO-d_6, 50 °C, rotamers): 18.8, 18.8, 19.2, 19.3, 23.7, 24.8, 24.9, 26.0, 28.3, 28.3, 61.3, 62.0, 65.5, 66.3, 80.6, 81.2, 92.8, 93.2, 126.7, 127.3, 127.4, 127.6, 128.0, 128.1, 136.1, 136.4, 150.8, 151.6, 170.5, 170.7; IR (ATR) ν 2962, 1712, 1405, 1351, 1163, 1125, 1091, 1051, 1026, 882, 767, 737, 696; UV (MeCN) λ_{max} (log ε) 252 (2.25), 257 (2.30); $[\alpha]_D^{20}$ -12.9 (c 0.89, $CHCl_3$); MS (ESI$^-$) m/z 320.1 (M-H$^-$); HRMS (ESI$^-$) m/z calcd for $C_{17}H_{22}NO_5$ 320.1503 (M-H$^+$), found 320.1504.

(4*S*,5*S*)-*N*^{2}-(Benzyloxycarbonyl)-*N*-(3',3'-diethoxypropan-1'-yl)-5-isopropyl-2,2-dimethyl-oxazolidine-4-carboxylic acid amide (21)

A solution of **19** (280 mg, 0.871 mmol, 1.0 equiv) in dry THF (5 mL) was treated at room temperature under argon atmosphere with HOBt (131 mg, 0.970 mmol, 1.1 equiv) and EDC hydrochloride (186 mg, 0.970 mmol, 1.1 equiv) and stirred for 30 min. Subsequently, 3,3-diethoxy-1-aminopropane (**20**, 0.18 mL, 1.1 mmol, 1.3 equiv) and DIPEA (0.22 mL, 1.3 mmol, 1.5 equiv) were added and stirring was continued for 16 h. The reaction mixture was diluted with EtOAc (20 mL) and washed with water (1 × 10 mL), aq. HCl (1 M, 1 × 10 mL), aq. sat. $NaHCO_3$ solution (1 × 10 mL) and water (1 × 10 mL) and dried over Na_2SO_4. The solvent was removed under reduced pressure and the resultant residue was purified by

column chromatography (petroleum ether/EtOAc 2:1) yielding 301 mg (0.668 mmol, 77%) of **21** as a white foam. TLC R_f 0.25 (petroleum ether/EtOAc 2:1); mp 93 °C; ^{1}H NMR (300 MHz, DMSO-d_6, 100 °C): 0.93 (d, 3H, *J* = 6.6 Hz, $CH(C\underline{H}_3)_2$), 0.97 (d, 3H, *J* = 6.6 Hz, $CH(C\underline{H}_3)_2$), 1.12 (t, 3H, *J* = 7.0 Hz, $OCH_2C\underline{H}_3$), 1.13 (t, 3H, *J* = 7.0 Hz, $OCH_2C\underline{H}_3$), 1.48 (s, 3H, 2-CH_3), 1.60-1.79 (m, 3H, $C\underline{H}(CH_3)_2$, 2'-H_2), 1.67 (s, 3H, 2-CH_3), 3.00-3.25 (m, 2H, 1'-H_2), 3.39-3.63 (m, 4H, $OC\underline{H}_2CH_3$), 3.83 (dd, 1H, *J* = 9.0, 6.0 Hz, 5-H), 4.31 (d, 1H, *J* = 6.0 Hz, 4-H), 4.50 (t, 1H, *J* = 5.5 Hz, 3'-H), 5.03 (d, 1H, *J* = 12.8 Hz, $C\underline{H}_aH_bPh$), 5.11 (d, 1H, *J* = 12.8 Hz, $CH_a\underline{H}_bPh$), 7.25-7.40 (m, 5H, Ph-H), 7.41-7.49 (m, 1H, NH); ^{13}C NMR (126 MHz, DMSO-d_6, 35 °C, rotamers): 15.2, 19.4, 19.4, 23.6, 24.8, 25.0, 26.2, 27.8, 27.9, 33.2, 33.3, 34.7, 34.7, 60.6, 60.7, 60.8, 61.8, 62.6, 65.4, 66.3, 81.0, 81.5, 92.8, 93.1, 100.3, 126.7, 127.3, 127.5, 127.7, 128.0, 128.2, 136.5, 151.0, 168.1; IR (ATR) ν 3327, 2975, 1710, 1402, 1346, 1257, 1221, 1124, 1049, 883, 767, 696; UV (MeCN) λ_{max} (log ε) 252 (2.30), 258 (2.34); $[\alpha]_D^{20}$ -15.1 (c 0.32, $CHCl_3$); MS (ESI^+) *m/z* 473.3 ($M+Na^+$); HRMS (ESI^+) *m/z* calcd for $C_{24}H_{38}N_2NaO_6$ 473.2622 ($M+Na^+$), found 473.2624.

(2*S*,3*S*)-*N*^{2}-(Benzyloxycarbonyl)-*N*-(3'-oxopropyl)-3-hydroxyleucine amide (22)

A solution of **21** (33 mg, 73 µmol) in CH_2Cl_2/TFA (9:1, 5 mL) was stirred for 2.5 h at room temperature. The reaction was quenched with aq. sat. $NaHCO_3$ solution (5 mL) and the aqueous layer was extracted with CH_2Cl_2 (3 × 5 mL). The combined organics were dried over $MgSO_4$ and the solvent was removed under reduced pressure. The resultant residue was purified by column chromatography (EtOAc) yielding 22 mg (65 µmol, 89%) of **22** as a white solid. TLC R_f 0.30 (EtOAc); mp 74 °C; ^{1}H NMR (300 MHz, CD_3CN, 70 °C): 0.91 (d, 3H, *J* = 6.7 Hz, 5-H_3), 0.94 (d, 3H, *J* = 6.7 Hz, 5-H_3), 1.76 (qqd, 1H, *J* = 6.7, 6.7, 6.7 Hz, 4-H), 2.60 (td, 2H, *J* = 6.4, 1.3 Hz, 2'-H_2), 3.35-3.57 (m, 3H, 3-H, 1'-H_2), 4.16 (dd, 1H, *J* = 8.8, 6.0 Hz, 2-H), 4.99-5.18 (m, 2H, NCO_2CH_2Ph), 6.20 (d, 1H, *J* = 8.8 Hz, NH), 7.16-7.41 (m, 5H,

Ph-H); ^{13}C NMR (126 MHz, CD_3CN, 50 °C): 17.6, 19.8, 31.1, 34.2, 43.7, 57.3, 67.6, 77.7, 128.7, 128.9, 129.0, 129.4, 137.9, 157.0, 173.0, 202.1; IR (ATR) ν 3329, 2959, 1707, 1663, 1403, 1347, 1256, 1091, 1047, 1027, 882, 697; UV (MeCN) λ_{max} (log ε) 251 (2.24), 258 (2.26); $[\alpha]_D^{20}$ -31.4 (c 0.13, $CHCl_3$); MS (ESI^+) *m/z* 337.2 ($M+H^+$), 359.2 ($M+Na^+$); MS (ESI^+) *m/z* calcd for $C_{17}H_{24}N_2O_5$ 359.1577 ($M+Na^+$), found 359.1579.

Benzyl (4*S*,5*S*)-*N*-(benzyloxycarbonyl)-5-isopropyl-2,2-dimethyloxazolidine-4-carboxylate (23)

A solution of **19** (280 mg, 0.871 mmol, 1.0 equiv), benzyl bromide (1.10 mL, 9.25 mmol, 10.6 equiv) and cesium carbonate (853 mg, 2.62 mmol, 3.0 equiv) in dry DMF (10 mL) was stirred at room temperature for 18 h. The reaction was quenched with water (150 mL) and the aqueous layer was extracted with Et_2O (3 × 75 mL). The combined organics were dried over Na_2SO_4 and the solvent was removed under reduced pressure. The resultant residue was purified by column chromatography (SiO_2, petroleum ether/EtOAc 10:1) yielding 249 mg (0.605 mmol, 70%) of **23** as a clear oil. TLC R_f 0.26 (petroleum ether/EtOAc 9:1); ^1H NMR (300 MHz, DMSO-d_6, 100 °C): 0.92 (d, 3H, *J* = 6.5 Hz, CH(C<u>H</u>$_3$)$_2$), 0.93 (d, 3H, *J* = 6.5 Hz, CH(C<u>H</u>$_3$)$_2$), 1.49 (s, 3H, 2-CH_3), 1.52-1.69 (m, 1H, C<u>H</u>(CH_3)$_2$), 1.62 (s, 3H, 2-CH_3), 3.92 (dd, 1H, *J* = 9.5, 5.8 Hz, 5-H), 4.53 (d, 1H, *J* = 5.8 Hz, 4-H), 4.95-5.21 (m, 4H, CH_2Ph), 7.18-7.45 (m, 10H, Ph-H); ^{13}C NMR (126 MHz, DMSO-d_6, 50 °C, rotamers): 18.6, 18.6, 19.2, 19.2, 23.7, 24.7, 24.8, 26.0, 28.1, 28.2, 61.2, 62.0, 65.8, 66.1, 66.1, 66.5, 80.8, 81.3, 93.0, 93.4, 126.9, 127.4, 127.7, 127.7, 127.8, 127.9, 127.9, 128.0, 128.0, 128.1, 135.0, 135.2, 136.0, 136.1, 150.6, 151.7, 169.1, 169.2; IR (ATR) ν 1746, 1712, 1403, 1347, 1262, 1216, 1161, 1124, 1088, 1052, 1027, 735, 695; UV (MeCN) λ_{max} (log ε) 206 (4.24), 252 (2.66), 258 (2.72), 262 (2.67); $[\alpha]_D^{24}$ -24.1 (c 0.36, $CHCl_3$); MS (ESI^+) *m/z* 412.2 ($M+H^+$), 434.2 ($M+Na^+$); HRMS (ESI^+) *m/z* calcd for $C_{24}H_{29}NNaO_5$ 434.1938 ($M+Na^+$), found 434.1939.

(2*S*,3*S*)-*N*-(Benzyloxycarbonyl)-3-hydroxyleucine benzyl ester (24)

A solution of **23** (240 mg, 0.583 mmol) in a mixture of $CHCl_3$ and TFA (24:1, 12.5 mL) was stirred at room temperature for 24 h. The reaction was quenched with aq. sat. $NaHCO_3$ solution (50 mL) and the aqueous layer was extracted with CH_2Cl_2 (3 × 30 mL). The combined organics were dried over Na_2SO_4 and the solvent was removed under reduced pressure. The resultant residue was purified by column chromatography (SiO_2, petroleum ether/EtOAc 3:1) yielding 152 mg (0.409 mmol, 71%) of **24** as a clear oil. TLC R_f 0.14 (petroleum ether/EtOAc 4:1); ^1H NMR (600 MHz, $CDCl_3$): 0.93 (d, 3H, *J* = 6.7 Hz, 5-H_3), 0.95 (d, 3H, *J* = 6.7 Hz, 5-H_3), 1.69 (dqq, 1H, *J* = 8.0, 6.7, 6.7 Hz, 4-H), 2.54 (bs, 1H, OH), 3.47 (dd, 1H, *J* = 8.0, 3.5 Hz, 3-H), 4.56 (dd, 1H, *J* = 8.0, 3.5 Hz, 2-H), 5.09 (s, 2H, CH_2Ph), 5.16 (d, 1H, *J* = 12.3 Hz, $C\underline{H_a}H_bPh$), 5.20 (d, 1H, *J* = 12.3 Hz, $CH_a\underline{H_b}Ph$), 5.83 (d, 1H, *J* = 8.0 Hz, NH), 7.24-7.38 (m, 10H, Ph-H); ^{13}C NMR (126 MHz, $CDCl_3$): 18.6, 19.1, 31.1, 56.7, 67.2, 67.3, 78.7, 128.0, 128.1, 128.1, 128.3, 128.4, 128.5, 134.9, 136.0, 155.9, 170.6; IR (ATR) ν 3425, 1704, 1498, 1214, 1187, 1173, 1050, 1005, 735, 695; UV (MeCN) λ_{max} (log ε) 207 (4.17), 253 (2.41), 258 (2.50); $[\alpha]_D^{20}$ +3.2 (c 2.3, $CHCl_3$); MS (ESI^+) *m/z* 372.2 ($M+H^+$), 394.2 ($M+Na^+$); HRMS (ESI^+) *m/z* calcd for $C_{21}H_{26}NO_5$ 372.1805 ($M+H^+$), found 372.1800.

6-Methylheptanoic acid (26)

A solution of **33** (189 mg, 0.814 mmol, 1.0 equiv) in MeOH (10 mL) was degassed with a stream of argon and subsequently treated with Pearlman's catalyst (114 mg, 81.5 µmol, 0.1 equiv). The reaction mixture was stirred for 5 h under hydrogen atmosphere (1 bar, balloon) at room temperature and then filtered through a SPARTAN™ syringe filter. The solvent of the filtrate was removed under reduced pressure and the resultant residue was dissolved in a small amount of EtOAc and dried over Na_2SO_4. The solvent was removed

under reduced pressure yielding 107 mg (0.742 mmol, 92%) of **26** as a clear liquid which was used without further purification. ^{1}H NMR (300 MHz, $CDCl_3$): 0.85 (d, 6H, *J* = 6.6 Hz, 7-H_3, 7-H_3), 1.11-1.22 (m, 2H, 5-H_2), 1.24-1.38 (m, 2H, 4-H_2), 1.52 (qqt, 1H, *J* = 6.6, 6.6, 6.6 Hz, 6-H), 1.60 (tt, 2H, *J* = 7.5, 7.5 Hz, 3-H_2), 2.33 (t, 2H, *J* = 7.5 Hz, 2-H_2); ^{13}C NMR (75 MHz, $CDCl_3$): 22.5, 24.9, 26.8, 27.8, 34.1, 38.5, 180.4; IR (ATR) ν 2953, 1707, 1466, 1412, 1286, 1238, 1166, 1113, 935; MS (ESI^-) *m/z* 143.1 ($M-H^+$); HRMS (ESI^-) *m/z* calcd for $C_8H_{15}O_2$ 143.1078 ($M-H^+$), found 143.1076.

(2*S*,3*S*)-*N*-(Benzyloxycarbonyl)-*O*-octanoyl-3-hydroxyleucine benzyl ester (27)

DIC (10 µL, 65 µmol, 2.4 equiv) was added at room temperature to a solution of **24** (10 mg, 27 µmol, 1.0 equiv), octanoic acid (**25**, 6.5 µL, 41 µmol, 1.5 equiv) and DMAP (7.8 mg, 64 µmol, 2.4 equiv) in dry THF (1 mL) under argon-atmosphere and stirred for 20 h at this temperature. The reaction mixture was then acidified with hydrochloric acid (0.5 M, 5 mL) and the aqueous layer was extracted with CH_2Cl_2 (3 × 5 mL). The combined organics were dried over Na_2SO_4 and the solvent was evaporated under reduced pressure. The resultant residue was purified by column chromatography (SiO_2, petroleum ether/EtOAc 4:1) yielding 8.4 mg (17 µmol, 63%) of **27** as a clear oil. TLC R_f 0.42 (petroleum ether/EtOAc 4:1); ^{1}H NMR (300 MHz, $CDCl_3$): 0.86 (t, 3H, *J* = 6.9 Hz, 8'-H_3), 0.88 (d, 3H, *J* = 6.6 Hz, 5-H_3), 0.99 (d, 3H, *J* = 6.7 Hz, 5-H_3), 1.16-1.35 (m, 8H, 4'-H_2-7'-H_2), 1.44-1.59 (m, 2H, 3'-H_2), 1.98-2.19 (m, 3H, 4-H, 2'-H_2), 4.69 (dd, 1H, *J* = 8.6, 3.5 Hz, 2-H), 4.83 (dd, 1H, *J* = 8.4, 3.5 Hz, 3-H), 5.09 (s, 2H, NCO_2CH_2Ph), 5.17 (s, 2H, CO_2CH_2Ph), 5.65 (d, 1H, *J* = 8.6 Hz, NH), 7.25-7.40 (m, 10H, Ph-H); ^{13}C NMR (126 MHz, $CDCl_3$): 14.1, 18.4, 19.2, 22.6, 28.9, 29.1, 31.7, 24.8, 29.4, 34.1, 55.7, 67.1, 67.4, 79.0, 128.0, 128.1, 128.3, 128.4, 128.4, 128.5, 135.0, 136.1, 155.5, 169.1, 173.5; IR (ATR) ν 1723, 1499, 1256, 1215, 1160, 1110, 1042, 1028, 1002, 736, 696; UV (MeCN) λ_{max} (log ε) 207 (4.20), 252 (2.40), 258 (2.51), 262 (2.44); $[\alpha]_D^{20}$ -15.4

(c 0.84, $CHCl_3$); MS (ESI^+) *m/z* 520.3 ($M+Na^+$); HRMS (ESI^+) *m/z* calcd for $C_{29}H_{39}NNaO_6$ 520.2670 ($M+Na^+$), found 520.2664.

(2*S*,3*S*)-*N*-(Benzyloxycarbonyl)-*O*-(6'-methylheptanoyl)-3-hydroxyleucine benzyl ester (28)

DIC (10 µL, 65 µmol, 2.4 equiv) was added at room temperature to a solution of **24** (10 mg, 27 µmol, 1.0 equiv), 6-methylheptanoic acid (**26**, 6.0 mg, 42 µmol, 1.5 equiv) and DMAP (8.0 mg, 66 µmol, 2.4 equiv) in dry THF (1 mL) under argon atmosphere and stirred for 16 h at this temperature. The reaction mixture was then acidified with hydrochloric acid (0.5 M, 5 mL) and the aqueous layer was extracted with CH_2Cl_2 (3 × 5 mL). The combined organics were dried over Na_2SO_4 and the solvent was evaporated under reduced pressure. The residue was purified by column chromatography (SiO_2, petroleum ether/EtOAc 4:1) yielding 13 mg (25 µmol, 94%) of **28** as a clear oil. TLC R_f 0.39 (petroleum ether/EtOAc 4:1); ^1H NMR (300 MHz, $CDCl_3$): 0.84 (d, 6H, *J* = 6.6 Hz, 7'-H_3, 7'-H_3), 0.87 (d, 3H, *J* = 6.7 Hz, 5-H_3), 0.99 (d, 3H, *J* = 6.7 Hz, 5-H_3), 1.06-1.31 (m, 4H, 4'-H_2, 5'-H_2), 1.40-1.59 (m, 3H, 3'-H_2, 6'-H), 1.97-2.24 (m, 3H, 4-H, 2'-H_2), 4.69 (dd, 1H, *J* = 8.5, 3.4 Hz, 2-H), 4.82 (dd, 1H, *J* = 8.3, 3.4 Hz, 3-H), 5.09 (s, 2H, NCO_2CH_2Ph), 5.16 (s, 2H, CO_2CH_2Ph), 5.63 (d, 1H, *J* = 8.5 Hz, NH), 7.22-7.42 (m, 10H, Ph-H); ^{13}C NMR (126 MHz, $CDCl_3$): 18.5, 19.3, 22.6, 22.6, 25.1, 27.0, 27.9, 29.4, 34.2, 38.6, 55.7, 67.1, 67.4, 79.0, 128.0, 128.1, 128.3, 128.4, 128.4, 128.5, 135.0, 136.1, 155.5, 169.1, 173.5; IR (ATR) ν 2954, 1724, 1499, 1215, 1163, 1002, 735, 695; UV (MeCN) λ_{max} (log ε) 206 (4.20), 258 (2.91); $[\alpha]_D^{20}$ -12.2 (c 12.4, MeOH); MS (ESI^+) *m/z* 520.3 ($M+Na^+$); HRMS (ESI^+) *m/z* calcd for $C_{29}H_{39}NNaO_6$ 520.2670 ($M+Na^+$), found 520.2663.

Methyl 4-methylvalerate (30)

Conc. sulfuric acid (4.2 mL, 79 mmol, 9.9 equiv) was added to a solution of 4-methylvaleric acid (1.00 mL, 7.95 mmol, 1.0 equiv) in MeOH (60 mL) at 0 °C. The cooling bath was removed and stirring was continued under reflux for 3 h. The reaction mixture was then diluted with aq. sat. $NaHCO_3$ solution (50 mL) and the aqueous layer was extracted with CH_2Cl_2 (3 × 50 mL). The combined organics were washed with water (1 × 50 mL) and dried over Na_2SO_4. The solvent was removed under reduced pressure yielding 1.02 g (7.84 mmol, 99%) of **30** as a clear liquid that was used without further purification. ^{1}H NMR (300 MHz, $CDCl_3$): 0.89 (d, 6H, J = 6.1 Hz, 5-H_3, 5-H_3), 1.46-1.65 (m, 3H, 3-H_2, 4-H), 2.31 (t, 2H, J = 7.6 Hz, 2-H_2), 3.66 (s, 3H, OCH_3); ^{13}C NMR (76 MHz, $CDCl_3$): 22.2, 27.7, 32.2, 33.8, 51.5, 174.5; IR (ATR) ν 2955, 1739, 1436, 1258, 1169, 1107; MS (ESI$^+$) *m/z* 153.1 (M+Na$^+$); HRMS (ESI$^+$) *m/z* calcd for $C_7H_{14}NaO_2$ 153.0886 (M+Na$^+$), found 153.0887.

4-Methylpentan-1-ol (31)

Lithium borohydride (4 M in THF, 35 mL, 0.14 mol, 4.0 equiv) was added at 0 °C to a solution of **30** (4.55 g, 35.0 mmol, 1.0 equiv) in dry Et_2O (200 mL) under argon atmosphere. Subsequently, dry MeOH (5.7 mL, 0.14 mol, 4.0 equiv) was added dropwise and the reaction mixture was stirred under reflux for 3 h. The reaction was carefully quenched with aq. sat. NH_4Cl solution (100 mL) at room temperature and the aqueous layer was extracted with Et_2O (3 × 100 mL). The combined organics were dried over Na_2SO_4 and the solvent was removed under reduced pressure. The resultant crude product was purified by distillation under reduced pressure yielding 2.42 g (23.6 mmol, 68%) of **31** as a clear liquid. Bp 45 °C (11 mbar); ^{1}H NMR (300 MHz, $CDCl_3$): 0.89 (d, 6H, J = 6.7 Hz, 5-H_3, 5-H_3), 1.17-1.28 (m, 2H, 3-H_2), 1.40 (bs, 1H, OH), 1.48-1.63 (m, 3H, 2-H_2, 4-H), 3.62 (t, 2H, J = 6.7 Hz, 1-H_2); ^{13}C NMR

(75 MHz, $CDCl_3$): 22.5, 27.8, 30.6, 34.9, 63.3; IR (ATR) ν 3336, 2953, 1468, 1385, 1366, 1055, 1020; MS (EI) *m/z* (%) 84 (11) $[M-H_2O]^+$, 69 (100) $[C_5H_{10}]^+$, 56 (96) $[C_4H_8]^+$.

Benzyl 6-methylhept-2-enoate (33)

Dry DMSO (0.28 mL, 3.9 mmol, 4.1 equiv) was added at −78 °C to a solution of oxalyl chloride (0.17 mL, 2.0 mmol, 2.1 equiv) in dry CH_2Cl_2 (10 mL) under argon atmosphere, and the mixture was stirred for 10 min. Subsequently, **31** (0.12 mL, 0.96 mmol, 1.0 equiv) was added and stirring was continued for 45 min at this temperature. The reaction mixture was treated with dry NEt_3 (1.10 mL, 7.89 mmol, 8.2 equiv) and the cooling bath was removed. At room temperature, benzyl 2-(triphenylphosphoranyliden)acetate **32** (522 mg, 1.27 mmol, 1.3 equiv) was added and stirring was continued at room temperature for 16 h. The reaction mixture was diluted with water (10 mL) and the aqueous layer was extracted with CH_2Cl_2 (3 × 10 mL). The combined organics were dried over $MgSO_4$ and the solvent was removed under reduced pressure. The resultant residue was purified by column chromatography (SiO_2, petroleum ether/EtOAc 19:1) yielding 189 mg (0.814 mmol, 85%) of **33** as a clear oil. TLC R_f 0.19 (petroleum ether/EtOAc 19:1); ^1H NMR (300 MHz, $CDCl_3$): 0.87 (d, 6H, *J* = 6.6 Hz, 7-H_3, 7-H_3), 1.20-1.38 (m, 2H, 5-H_2), 1.46-1.72 (m, 1H, 6-H), 2.13-2.25 (m, 2H, 4-H_2), 5.16 (s, 2H, CH_2Ph), 5.85 (dt, 1H, *J* = 15.6, 1.6 Hz, 2-H), 7.01 (dt, 1H, *J* = 15.6, 6.9 Hz, 3-H), 7.26-7.43 (m, 5H, Ph-H); ^{13}C NMR (76 MHz, $CDCl_3$): 22.3, 27.5, 30.1, 37.0, 66.0, 120.7, 128.1, 128.2, 128.5, 136.2, 150.3, 166.5; IR (ATR) ν 1717, 1226, 1162, 1128, 1025, 984, 736, 695; UV (MeCN) λ_{max} (log ε) 207 (4.29), 273 (3.51); MS (ESI^+) *m/z* 233.2 ($M+H^+$), 255.2 ($M+Na^+$); HRMS (ESI^+) *m/z* calcd for $C_{15}H_{21}O_2$ 233.1536 ($M+H^+$), found 233.1537.

(2*R*,3*S*)-1-*O*-(*tert*-Butyldimethylsilyl)-2-(fluorenylmethyloxycarbonylamino)-4-methyl-pentane-1,3-diol (34)

Fmoc chloride (4.71 g, 18.2 mmol, 3.0 equiv) was added at 0 °C to a solution of **5** (1.50 g, 6.06 mmol, 1.0 equiv) and NEt_3 (4.23 mL, 30.3 mmol, 5.0 equiv) in dry THF (70 mL) under argon atmosphere and the reaction mixture was stirred for 5 h at this temperature. The reaction mixture was then diluted with water (70 mL) and the aqueous layer was extracted with EtOAc (3 × 70 mL). The combined organics were dried over Na_2SO_4 and the solvent was removed under reduced pressure. The resultant residue was purified by column chromatography (SiO_2, petroleum ether/EtOAc 9:1 → 6:1) yielding 2.39 g (5.08 mmol, 84%) of **34** as a pale yellow oil. TLC R_f 0.33 (petroleum ether/EtOAc 4:1); ^{1}H NMR (300 MHz, C_6D_6): -0.04 (s, 3H, Si(C<u>H</u>$_3$)$_2$C(CH$_3$)$_3$), -0.06 (s, 3H, Si(C<u>H</u>$_3$)$_2$C(CH$_3$)$_3$), 0.76 (d, 3H, *J* = 6.7 Hz, 5-H$_3$), 0.86 (s, 9H, Si(CH$_3$)$_2$C(C<u>H</u>$_3$)$_3$), 1.04 (d, 3H, *J* = 6.7 Hz, 5-H$_3$), 1.65 (qqd, 1H, *J* = 6.7, 6.7, 6.5 Hz, 4-H), 2.63 (d, 1H, *J* = 7.4 Hz, OH), 3.21 (ddd, 1H, *J* = 7.4, 6.5, 6.5 Hz, 3-H), 3.76 (t, 2H, *J* = 2.7 Hz, 1-H$_2$), 3.69-3.90 (m, 1H, 2-H), 4.07 (t, 1H, *J* = 6.5 Hz, NCO$_2$CH$_2$C<u>H</u>Aryl), 4.38-4.53 (m, 2H, NCO$_2$C<u>H</u>$_2$CHAryl), 5.25 (d, 1H, *J* = 8.4 Hz, NH), 7.11-7.26 (m, 4H, Aryl-H), 7.39-7.51 (m, 2H, Aryl-H), 7.53-7.60 (m, 2H, Aryl-H); ^{13}C NMR (76 MHz, C_6D_6): -5.7, -5.6, 18.1, 18.2, 19.4, 25.9, 31.5, 47.8, 53.0, 63.7, 66.6, 78.5, 120.2, 125.4, 127.3, 127.3, 141.8, 144.5, 144.5, 156.0; IR (ATR) ν 2953, 1705, 1251, 1069, 835, 777, 757, 767; UV (MeCN) λ_{max} (log ε) 206 (4.69), 265 (4.30), 289 (3.70), 300 (3.76); $[\alpha]_D^{25}$ -19.2 (c 0.63, $CHCl_3$); MS (ESI$^+$) *m/z* 492.3 (M+Na$^+$); HRMS (ESI$^+$) *m/z* calcd for $C_{27}H_{39}NNaO_4Si$ 492.2541 (M+Na$^+$), found 492.2546.

(2*R*,3*S*)-3-*O*-Acryloyl-1-*O*-(*tert*-butyldimethylsilyl)-2-(fluorenylmethyloxycarbonyl-amino)-4-methyl-pentane-1,3-diol (36)

Acryloyl chloride (**35**, 0.42 mL, 5.2 mmol, 4.0 equiv) was added dropwise at 0 °C to a solution of **34** (600 mg, 1.28 mmol, 1.0 equiv) and DIPEA (1.80 mL, 10.6 mmol, 8.3 equiv) in dry CH_2Cl_2 (30 mL) under argon atmosphere and the reaction mixture was stirred for 4 h at this temperature. The reaction was quenched with aq. sat. $NaHCO_3$ solution (30 mL) and the aqueous layer was extracted with CH_2Cl_2 (3 × 30 mL). The combined organics were dried over Na_2SO_4 and the solvent was removed under reduced pressure. The resultant residue was purified by column chromatography (SiO_2, petroleum ether/EtOAc 6:1) yielding 629 mg (1.20 mmol, 94%) of **36** as a clear oil. TLC R_f 0.47 (petroleum ether/EtOAc 4:1); ^{1}H NMR (300 MHz, C_6D_6): -0.01 (s, 3H, Si(C<u>H</u>$_3$)$_2$C(CH$_3$)$_3$), 0.02 (s, 3H, Si(C<u>H</u>$_3$)$_2$C(CH$_3$)$_3$), 0.90 (d, 3H, *J* = 7.0 Hz, 5-H$_3$), 0.94 (s, 9H, Si(CH$_3$)$_2$C(C<u>H</u>$_3$)$_3$), 1.03 (d, 3H, *J* = 6.7 Hz, 5-H$_3$), 1.83-2.01 (m, 1H, 4-H), 3.47 (dd, 1H, *J* = 10.3, 3.5 Hz, 1-H$_a$), 3.53 (dd, 1H, *J* = 10.3, 3.5 Hz, 1-H$_b$), 4.05 (t, 1H, *J* = 6.5 Hz, NCO$_2$CH$_2$C<u>H</u>Aryl), 4.09-4.22 (m, 1H, 2-H), 4.44 (d, 2H, *J* = 6.5 Hz, NCO$_2$C<u>H</u>$_2$CHAryl), 4.88 (d, 1H, *J* = 8.8 Hz, NH), 5.13 (dd, 1H, *J* = 8.8, 3.5 Hz, 3-H), 5.26 (dd, 1H, *J* = 10.4, 1.5 Hz, 3'-H$_a$), 5.92 (dd, 1H, *J* = 17.3, 10.4 Hz, 2'-H), 6.25 (dd, 1H, *J* = 17.3, 1.5 Hz, 3'-H$_b$), 7.13-7.28 (m, 4H, Aryl-H), 7.40-7.52 (m, 2H, Aryl-H), 7.54-7.61 (m, 2H, Aryl-H); ^{13}C NMR (126 MHz, C_6D_6): -5.4, -5.2, 16.5, 18.6, 20.2, 26.2, 29.5, 47.9, 53.2, 62.5, 66.7, 76.0, 120.2, 120.2, 125.3, 125.4, 127.2, 127.3, 127.9, 128.1, 128.6, 130.5, 141.8, 144.5, 156.0, 165.2; IR (ATR) ν 1722, 1506, 1404, 1253, 1185, 1044, 982, 835, 776, 758, 737; UV (MeCN) λ_{max} (log ε) 205 (4.73), 265 (4.27), 289 (3.68), 300 (3.74); $[\alpha]_D^{20}$ -20.4 (c 1.4, $CHCl_3$); MS (ESI$^+$) *m/z* 546.3 (M+Na$^+$); HRMS (ESI$^+$) *m/z* calcd for $C_{30}H_{41}NNaO_5Si$ 546.2646 (M+Na$^+$), found 546.2643.

(2*R*,3*S*)-3-*O*-Acryloyl-2-(fluorenylmethyloxycarbonylamino)-4-methyl-pentane-1,3-diol (37)

A solution of **36** (125 mg, 0.239 mmol, 1.0 equiv) in dry MeOH (1.5 mL) under argon atmosphere was treated at 0 °C with acetyl chloride (4.3 µL, 61 µmol, 0.3 equiv) and stirring was continued for 3 h at this temperature. The reaction was quenched with aq. sat. $NaHCO_3$ solution (5 mL) and the aqueous layer was extracted with EtOAc (3 × 5 mL). The combined organics were dried over Na_2SO_4 and the solvent was removed under reduced pressure. The resultant residue was purified by column chromatography (SiO_2, petroleum ether/EtOAc 2:1) yielding 91 mg (0.22 mmol, 93%) of **37** as a white foam. TLC R_f 0.42 (petroleum ether/EtOAc 1:1); mp 48 °C; ^1H NMR (300 MHz, $CDCl_3$): 0.92 (d, 3H, *J* = 6.8 Hz, 5-H_3), 1.01 (d, 3H, *J* = 6.8 Hz, 5-H_3), 1.97 (qqd, 1H, *J* = 6.8, 6.8, 3.1 Hz, 4-H), 2.78 (bs, 1H, OH), 3.44-3.67 (m, 2H, 1-H_2), 3.89 (ddd, 1H, *J* = 9.5, 9.3, 2.2 Hz, 2-H), 4.20 (dd, 1H, *J* = 7.1, 6.7 Hz, $NCO_2CH_2C\underline{H}Aryl$), 4.38 (dd, 1H, *J* = 10.6, 6.7 Hz, $NCO_2C\underline{H}_aH_bCHAryl$), 4.46 (dd, 1H, *J* = 10.6, 7.1 Hz, $NCO_2CH_a\underline{H}_bCHAryl$), 4.79 (dd, 1H, *J* = 9.3, 3.0 Hz, 3-H), 5.42 (d, 1H, *J* = 9.5 Hz, NH), 5.92 (dd, 1H, *J* = 10.4, 1.0 Hz, 3'-H_a), 6.15 (dd, 1H, *J* = 17.3, 10.4 Hz, 2'-H), 6.47 (dd, 1H, *J* = 17.3, 1.0 Hz, 3'-H_b), 7.25-7.33 (m, 2H, Aryl-H), 7.35-7.43 (m, 2H, Aryl-H), 7.53-7.62 (m, 2H, Aryl-H), 7.70-7.79 (m, 2H, Aryl-H); ^{13}C NMR (126 MHz, $CDCl_3$): 15.8, 20.0, 28.3, 47.3, 52.4, 61.5, 66.7, 76.7, 119.9, 120.0, 125.0, 125.0, 127.0, 127.6, 127.7, 132.4, 141.3, 143.7, 143.8, 156.1, 167.4; IR (ATR) ν 3326, 2963, 1697, 1530, 1449, 1404, 1266, 1188, 1044, 980, 758, 738; UV (MeCN) λ_{max} (log ε) 205 (4.72), 265 (4.27), 289 (3.68), 300 (3.75); $[\alpha]_D^{20}$ -34.5 (c 0.63, $CHCl_3$); MS (ESI^+) *m/z* 432.2 ($M+Na^+$); HRMS (ESI^+) *m/z* calcd for $C_{24}H_{27}NNaO_5$ 432.1781 ($M+Na^+$), found 432.1790.

(2*S*,3*S*)-*O*-Acryloyl-*N*-(fluorenylmethyloxycarbonyl)-3-hydroxyleucine (38)

A solution of **37** (50 mg, 0.12 mmol, 1.0 equiv) in a mixture of acetone (1.5 mL) and aq. sat. $NaHCO_3$ solution (0.4 mL) was treated at 0 °C with sodium bromide (1.3 mg, 13 µmol, 0.1 equiv) and TEMPO (0.4 mg, 3 µmol, 0.02 equiv). Subsequently, trichlorocyanuric acid (57 mg, 0.25 mmol, 2.0 equiv) was added in portions over 13 min and stirring was continued for 4 h at 0 °C. The reaction mixture was neutralized with aq. sat. NH_4Cl solution (2 mL) and concentrated under reduced pressure. The resultant residue was dissolved in hydrochloric acid (0.5 M, 5 mL) and extracted with EtOAc (3 × 5 mL). The combined organics were dried over Na_2SO_4 and the solvent was removed under reduced pressure. The resultant residue was purified by column chromatography (SiO_2, CH_2Cl_2/MeOH 19:1 → 9:1) yielding 43 mg (0.10 mmol, 85%) of **38** as a white solid. TLC R_f 0.11 (CH_2Cl_2-MeOH 9:1); mp 89 °C; ^1H NMR (600 MHz, DMSO-d_6, 35 °C): 0.83 (d, 3H, J = 6.8 Hz, 5-H_3), 0.93 (d, 3H, J = 6.8 Hz, 5-H_3), 2.19 (qqd, 1H, J = 6.8, 6.8, 6.8 Hz, 4-H), 4.13-4.25 (m, 3H, 2-H, $NCO_2CH_2C\underline{H}$Aryl, $NCO_2C\underline{H}_aH_b$CHAryl), 4.27-4.35 (m, 1H, $NCO_2CH_a\underline{H}_b$CHAryl), 5.02-5.11 (m, 1H, 3-H), 5.88 (dd, 1H, J = 10.4, 1.2 Hz, 3'-H_a), 6.10 (dd, 1H, J = 17.3, 10.4 Hz, 2'-H), 6.29 (dd, 1H, J = 17.3, 1.2 Hz, 3'-H_b), 7.09 (bs, 1H, NH), 7.28-7.35 (m, 2H, Aryl-H), 7.37-7.44 (m, 2H, Aryl-H), 7.60-7.72 (m, 2H, Aryl-H), 7.85-7.91 (m, 2H, Aryl-H); ^{13}C NMR (126 MHz, DMSO-d_6, 35 °C): 17.8, 19.4, 28.3, 46.6, 55.8, 65.7, 77.9, 119.9, 119.9, 125.0, 125.1, 126.9, 126.9, 127.4, 128.5, 130.8, 140.5, 143.6, 143.6, 155.3, 164.9, 170.8; IR (ATR) ν 1701, 1589, 1405, 1263, 1191, 1049, 981, 759, 738, 539; UV (MeCN) λ_{max} (log ε) 205 (4.47), 266 (4.04), 300 (3.50); $[\alpha]_D^{20}$ -15.7 (c 0.86, $CHCl_3$); MS (ESI^-) *m/z* 422.2 ($M-H^+$); HRMS (ESI^-) *m/z* calcd for $C_{24}H_{24}NO_6$ 422.1609 ($M-H^+$), found 422.1609.

(2*S*,3*S*)-*O*-Acryloyl-*N*-(fluorenylmethyloxycarbonyl)-3-hydroxyleucine benzyl ester (39)

A solution of **38** (45 mg, 0.11 mmol, 1.0 equiv) in dry DMF (1 mL) under argon atmosphrere was treated at room temperature with cesium carbonate (103 mg, 0.316 mmol, 1.0 equiv) and benzyl bromide (0.13 mL, 1.1 mmol, 10.3 equiv) and stirring was continued for 17 h. The reaction mixture was then diluted with water (20 mL) and the aqueous layer was extracted with EtOAc (3 × 10 mL). The combined organics were dried over Na_2SO_4 and the solvent was removed under reduced pressure. The resultant residue was purified by column chromatography (SiO_2, petroleum ether/EtOAc 6:1) yielding 53 mg (0.10 mmol, 97%) of **39** as a clear oil. TLC R_f 0.34 (petroleum ether/EtOAc 4:1); ^{1}H NMR (300 MHz, $CDCl_3$): 0.91 (d, 3H, *J* = 6.7 Hz, 5-H_3), 1.03 (d, 3H, *J* = 6.7 Hz, 5-H_3), 2.10 (qqd, 1H, *J* = 8.1, 6.7, 6.7 Hz, 4-H), 4.21 (t, 1H, *J* = 7.1 Hz, $NCO_2CH_2C\underline{H}Aryl$), 4.32-4.38 (m, 2H, $NCO_2C\underline{H_2}CHAryl$), 4.75 (dd, 1H, *J* = 8.6, 3.7 Hz, 2-H), 4.96 (dd, 1H, *J* = 8.1, 3.7 Hz, 3-H), 5.18 (s, 2H, CH_2Ph), 5.71 (d, 1H, *J* = 8.6 Hz, NH), 5.80 (dd, 1H, *J* = 10.4, 1.5 Hz, 3'-H_a), 5.99 (dd, 1H, *J* = 17.2, 10.4 Hz, 2'-H), 6.32 (dd, 1H, *J* = 17.2, 1.5 Hz, 3'-H_b), 7.24-7.43 (m, 9H, Aryl-H), 7.53-7.60 (m, 2H, Aryl-H), 7.72-7.78 (m, 2H, Aryl-H); ^{13}C NMR (126 MHz, $CDCl_3$): 18.3, 19.2, 29.6, 47.1, 55.6, 67.3, 67.5, 79.4, 119.9, 119.9, 119.9, 125.1, 125.1, 127.0, 127.0, 127.6, 128.4, 128.5, 131.6, 134.9, 140.2, 141.2, 143.6, 143.7, 155.5, 165.7, 169.1; IR (ATR) ν 1720, 1508, 1449, 1404, 1257, 1182, 1045, 983, 757, 739, 697; UV (MeCN) λ_{max} (log ε) 205 (4.75), 266 (4.25), 299 (3.67); $[\alpha]_D^{20}$ -19.6 (c 1.5, $CHCl_3$); MS (ESI^+) *m/z* 514.4 ($M+H^+$), 536.4 ($M+Na^+$); HRMS (ESI^+) *m/z* calcd for $C_{31}H_{31}NNaO_6$ 536.2044 ($M+Na^+$), found 536.2044.

(2*S*,3*S*)-*O*-((*E*)-Non-2-enoyl)-*N*-(fluorenylmethyloxycarbonyl)-3-hydroxyleucine benzyl ester (40)

A solution of **39** (10 mg, 19 µmol, 1.0 equiv), 1-octene (7.0 µL, 45 µmol, 2.3 equiv) and Grubbs 2nd generation catalyst (1.7 mg, 2.0 µmol, 0.1 equiv) in degassed CH_2Cl_2 (2 mL)

under argon atmosphere was stirred under reflux for 4 h. The solvent was removed under reduced pressure and the resultant residue was purified by column chromatography (SiO_2, petroleum ether/EtOAc 9:1) yielding 7.3 mg (12 µmol, 63%) of **40** as a clear oil. TLC R_f 0.26 (petroleum ether/EtOAc 4:1); ^{1}H NMR (300 MHz, $CDCl_3$): 0.88 (t, 3H, *J* = 7.1 Hz, 9'-H_3), 0.91 (d, 3H, *J* = 6.8 Hz, 5-H_3), 1.04 (d, 3H, *J* = 6.8 Hz, 5-H_3), 1.19-1.35 (m, 6H, 6'-H_2-8'-H_2), 1.36-1.49 (m, 2H, 5'-H_2), 2.03-2.20 (m, 1H, 4-H), 2.15 (dt, 2H, *J* = 7.1, 6.9 Hz, 4'-H_2), 4.21 (t, 1H, *J* = 7.4 Hz, $NCO_2CH_2C\underline{H}Aryl$), 4.27-4.43 (m, 2H, $NCO_2C\underline{H}_2CHAryl$), 4.74 (dd, 1H, *J* = 8.7, 3.6 Hz, 2-H), 4.92 (dd, 1H, *J* = 8.1, 3.6 Hz, 3-H), 5.18 (s, 2H, CH_2Ph), 5.69 (d, 1H, *J* = 15.6 Hz, 2'-H), 5.77 (d, 1H, *J* = 8.7 Hz, NH), 6.92 (dt, 1H, *J* = 15.6, 6.9 Hz, 3'-H), 7.22-7.44 (m, 9H, Aryl-H), 7.51-7.61 (m, 2H, Aryl-H), 7.71-7.79 (m, 2H, Aryl-H); ^{13}C NMR (126 MHz, $CDCl_3$): 14.0, 18.3, 19.2, 22.5, 28.9, 31.6, 27.8, 29.6, 32.3, 47.1, 55.7, 67.3, 67.4, 79.0, 119.9, 119.9, 120.2, 125.2, 125.2, 127.0, 127.1, 127.7, 127.7, 128.4, 128.4, 128.5, 135.1, 140.2, 141.3, 143.8, 143.8, 151.1, 155.6, 166.4, 169.4; IR (ATR) ν 1718, 1500, 1449, 1254, 1220, 1190, 1163, 1011, 757, 738, 696; UV (MeCN) λ_{max} (log ε) 207 (4.79), 266 (4.24), 299 (3.68); $[\alpha]_D^{20}$ -17.9 (c 0.71, $CHCl_3$); MS (ESI^+) *m/z* 598.1 ($M+H^+$), 620.1 ($M+Na^+$); HRMS (ESI^+) *m/z* calcd for $C_{37}H_{43}NNaO_6$ 620.2983 ($M+Na^+$), found 620.2984.

(2*S*,3*S*)-*O*-(Nonanoyl)-*N*-(fluorenylmethyloxycarbonyl)-3-hydroxyleucine (41)

To a solution of **40** (7.0 mg, 12 µmol, 1.0 equiv) in MeOH (1 mL), Pd/C (10%, 1.3 mg, 1.2 µmol, 0.1 equiv) was added at room temperature under argon atmosphere. The argon atmosphere was replaced by a hydrogen atmosphere, and the reaction mixture was stirred for 16 h under hydrogen atmosphere (1 bar, balloon). The reaction mixture was then filtered through a SPARTAN™ syringe filter. The solvent of the filtrate was removed under reduced pressure yielding 4.5 mg (8.8 µmol, 75%) of **41** as a white solid. ^{1}H NMR (600 MHz, $CDCl_3$): 0.83 (t, 3H, *J* = 7.0 Hz, 9'-H_3), 0.92 (d, 3H, *J* = 6.4 Hz, 5-H_3), 0.98 (d, 3H, *J* =

5.9 Hz, 5-H_3), 1.14-1.32 (m, 10H, 4'-H_2-8'-H_2), 1.51-1.63 (m, 2H, 3'-H_2), 2.05-2.15 (m, 1H, 4-H), 2.21-2.33 (m, 2H, 2'-H_2), 4.16-4.25 (m, 2H, $NCO_2C\underline{H}_aH_bCHAryl$, $NCO_2CH_2C\underline{H}Aryl$), 4.32-4.38 (m, 1H, $NCO_2CH_a\underline{H}_bCHAryl$), 4.52-4.59 (m, 1H, 2-H), 4.89-4.96 (m, 1H, 3-H), 5.99 (d, 1H, *J* = 6.7 Hz, NH), 7.25-7.32 (m, 2H, Aryl-H), 7.33-7.38 (m, 2H, Aryl-H), 7.52-7.59 (m, 2H, Aryl-H), 7.69-7.76 (m, 2H, Aryl-H); ^{13}C NMR (126 MHz, $CDCl_3$): 14.1, 18.2, 19.3, 22.6, 29.1, 29.2, 29.2, 31.8, 24.9, 28.9, 34.4, 47.1, 56.0, 67.2, 78.9, 119.9, 125.2, 126.9, 126.9, 127.0, 127.6, 141.2, 143.8, 143.9, 156.1, 173.7, 174.4; MS (ESI^-) *m/z* 508.3 ($M-H^+$), ($M+Na^+$); HRMS (ESI^-) *m/z* calcd for $C_{30}H_{38}NO_6$ 508.2705 ($M-H^+$), found 508.2702.

Synthesis of racemic HPLC reference S1 and HPLC analysis of 3

Synthesis of S1

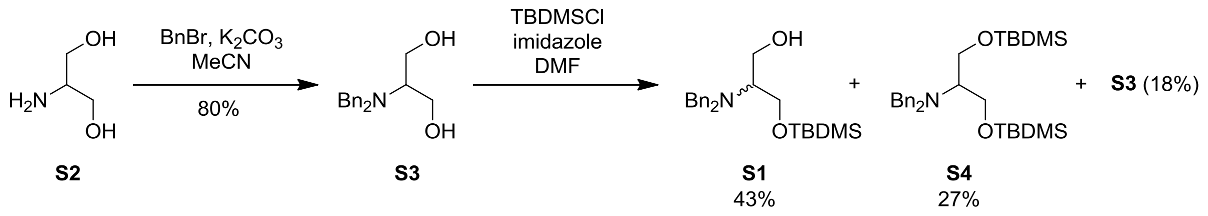

2-(*N*,*N*-Dibenzylamino)-1,3-propanediol (S3)

To a solution of 2-amino-1,3-propanediol (**S2**, 299 mg, 3.28 mmol, 1.0 equiv) in MeCN (10 mL), K_2CO_3 (2.27 g, 16.4 mmol, 5.0 equiv) and benzyl bromide (0.98 mL, 1.4 g, 8.3 mmol, 2.5 equiv) were added at room temperature. The reaction mixture was stirred at this temperature for 18 h and then diluted with water (10 mL). The aqueous layer was extracted with EtOAc (3 × 10 mL) and the combined organics were dried over Na_2SO_4. The solvent was removed under reduced pressure and the resultant residue was purified by column chromatography (SiO_2, petroleum ether/EtOAc 1:1) to yield 716 mg (2.64 mmol, 80%) of **S3** as a white solid. TLC R_f 0.20 (petroleum ether/EtOAc 1:1); mp 113 °C; ^{1}H NMR (300 MHz, C_6D_6): 2.30 (bs, 2H, OH), 2.93 (tt, 1H, J = 7.5, 5.9 Hz, 2-H), 3.37 (dd, 2H, J = 10.8, 5.9 Hz, 1-H_a, 3-H_a), 3.48 (dd, 2H, J = 10.8, 7.5 Hz, 1-H_b, 3-H_b), 3.53-3.66 (m, 4H, CH_2Ph), 7.01-7.32 (m, 10H, Ph-H); ^{13}C NMR (76 MHz, C_6D_6): 54.4, 60.2, 60.3, 127.4, 128.7, 129.2, 140.1; IR (ATR) ν 3245, 1493, 1451, 1049, 1025, 999, 741, 730, 695; UV (MeCN) λ_{max} (log ε) 206 (4.28), 259 (2.77); MS (ESI$^+$) *m/z* 272.2 (M+H$^+$), 294.2 (M+Na$^+$); HRMS (ESI$^+$) *m/z* calcd for $C_{17}H_{21}NNaO_2$ 294.1465 (M+Na$^+$), found 294.1466.

***rac*-1-*O*-(*tert*-Butyldimethylsilyl)-2-(dibenzylamino)-1,3-propanediol (S1)**

A solution of **S3** (600 mg, 2.21 mmol, 1.0 equiv), imidazole (168 mg, 2.47 mmol, 1.1 equiv) and *tert*-butyldimethylsilyl chloride (368 mg, 2.44 mmol, 1.1 equiv) in dry DMF (3 mL) under an argon atmosphere was stirred at room temperature for 24 h. The reaction mixture was treated with water (5 mL) and the aqueous layer was extracted with EtOAc (3 × 5 mL). The combined organics were dried over Na_2SO_4 and the solvent was removed under reduced pressure. The resultant crude product was purified by column chromatography (SiO_2, petroleum ether/EtOAc 10:1 → 1:1) to yield mono-silylated **S1** (361 mg, 0.936 mmol, 43%) as a clear oil, di-silylated **S4** (300 mg, 0.600 mmol, 27%) as a clear oil and reisolated starting material **S3** (105 mg, 0.387 mmol, 18%). **S1**: TLC R_f 0.16 (petroleum ether/EtOAc 10:1); ^{1}H NMR (300 MHz, C_6D_6): -0.01 (s, 3H, $Si(C\underline{H}_3)_2(C(CH_3)_3)$), 0.00 (s, 3H, $Si(C\underline{H}_3)_2(C(CH_3)_3)$), 0.93 (s, 9H, $Si(CH_3)_2(C(C\underline{H}_3)_3)$), 2.56 (bs, 1H, OH), 3.04 (dddd, 1H, J = 8.1, 6.1, 6.1, 6.1 Hz, 2-H), 3.47-3.64 (m, 4H, 1-H_2, 3-H_2), 3.59 (d, 2H, J = 13.5 Hz, $C\underline{H}_aH_bPh$), 3.81 (d, 2H, J = 13.5 Hz, $CH_a\underline{H}_bPh$), 7.03-7.11 (m, 2H, Ph-H), 7.12-7.21 (m, 4H, Ph-H), 7.28-7.35 (m, 4H, Ph-H); ^{13}C NMR (75 MHz, C_6D_6): -5.5, -5.5, 18.3, 26.0, 54.6, 59.9, 60.3, 61.5, 127.3, 128.7, 129.2, 140.3; IR (ATR) ν 1252, 1090, 1070, 1026, 834, 775, 744, 726, 696; UV (MeCN) λ_{max} (log ε) 205 (4.29); MS (ESI$^+$): *m/z* 386.3 (M+H$^+$); HRMS (ESI$^+$) *m/z* calcd for $C_{23}H_{36}NO_2Si$ 386.2510 (M+H$^+$), found 386.2507.

HPLC analysis of 3

For the determination of enantiomeric purity, HPLC analysis of isolated Grignard reduction product **3** was performed under the following conditions: Chiralpak™ IB column, *n*-hexane-*i*-PrOH 98:2, flow 0.8 mL/min. The racemic reference S1 (vide supra) was also injected to prove successful separation of the enantiomers under these conditions (Figure S1).

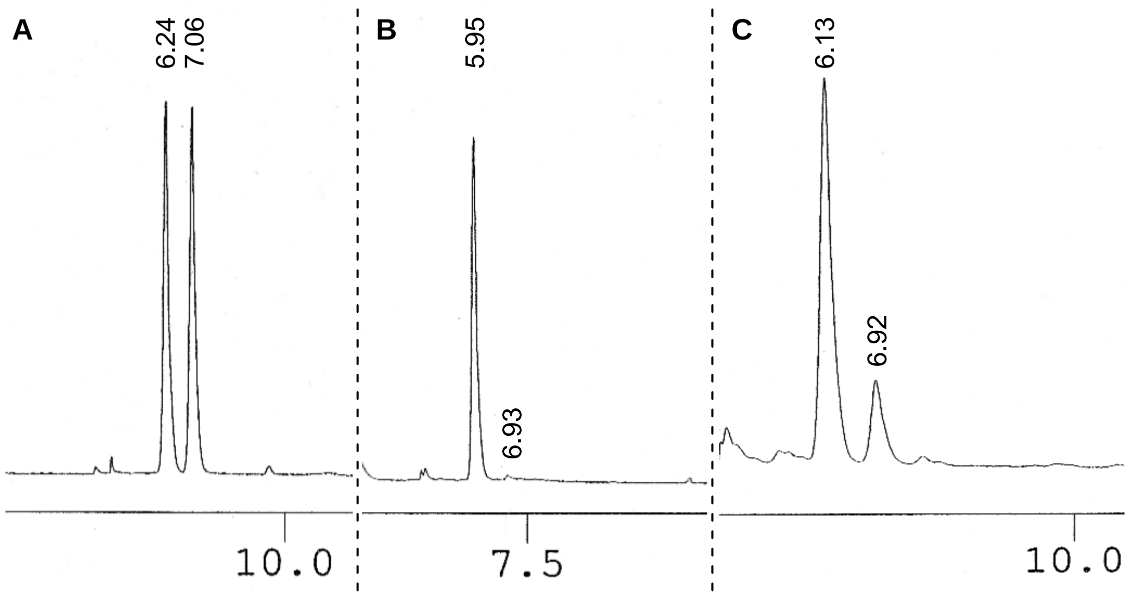

Figure S1: HPLC chromatograms; (A) racemic reference **S1**; (B) reisolated alcohol **3** after first oxidation-addition cycle (er = 99:1); (C) reisolated alcohol **3** after second oxidation-addition cycle (er = 78:22); numbers: retention times in minutes.

Determination of the X-ray crystal structure of compound 6

Single crystals were selected and covered with perfluorated polyether oil on a microscope slide, which was cooled with a nitrogen gas flow using the XTEMP2 to avoid melting of the crystals [1-3]. An appropriate crystal was selected using a polarized microscope, mounted on the tip of a MITEGEN™ MicroMount, fixed to a goniometer head and shock-cooled by the crystal cooling device. The data for **6** were collected from a shock-cooled crystal at 100(2) K on a BRUKER TXS-Mo rotating anode (used Mo-*K*α radiation, λ = 71.073 pm) with mirror optics and APEX II detector with a D8 goniometer. The data of **6** were integrated with SAINT[4] and an empirical absorption correction (SADABS) [5] was applied. The structure was solved by direct methods (SHELXS-97) and refined by full-matrix least-squares methods against *F*2 (SHELXL-97) [6-8]. All non-hydrogen atoms were refined with anisotropic displacement parameters. The hydrogen atoms were refined isotropically on calculated positions using a riding model with their U_{iso} values constrained to equal to 1.5 times the U_{eq} of their pivot atoms for terminal sp^3 carbon atoms and 1.2 times for all other carbon atoms.

^{1}H and ^{13}C NMR spectra of novel compounds

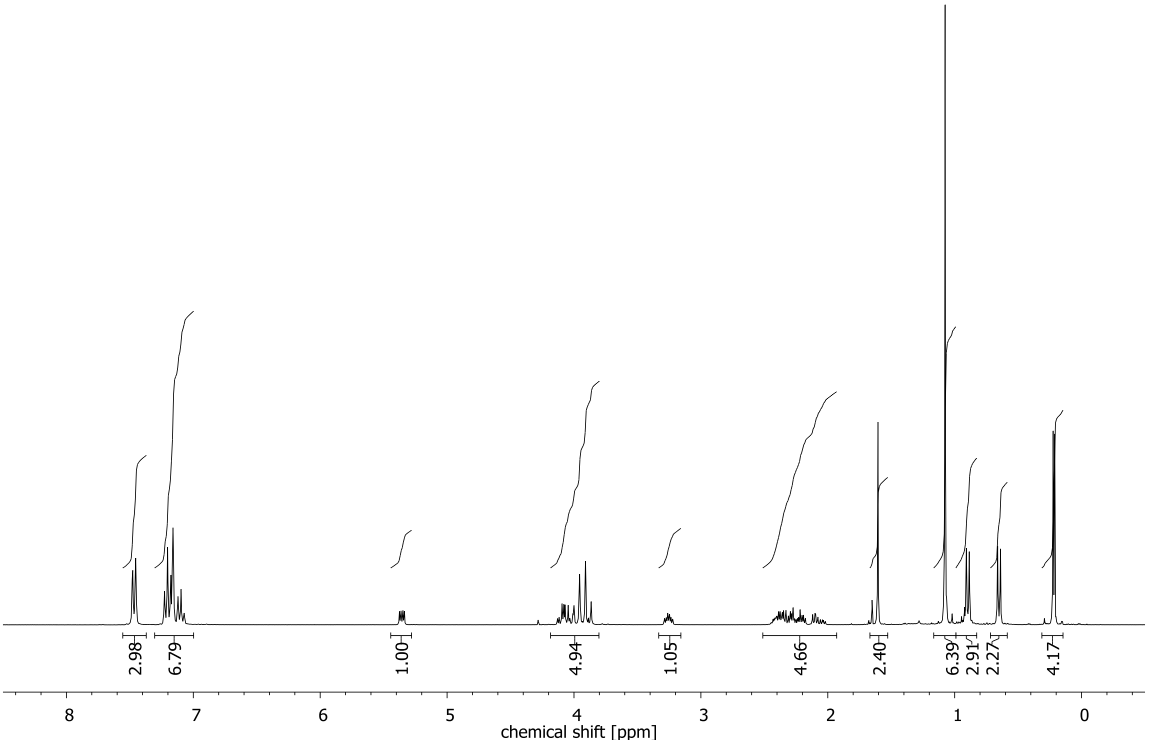

^{1}H NMR spectrum of **6** (300 MHz, C_6D_6)

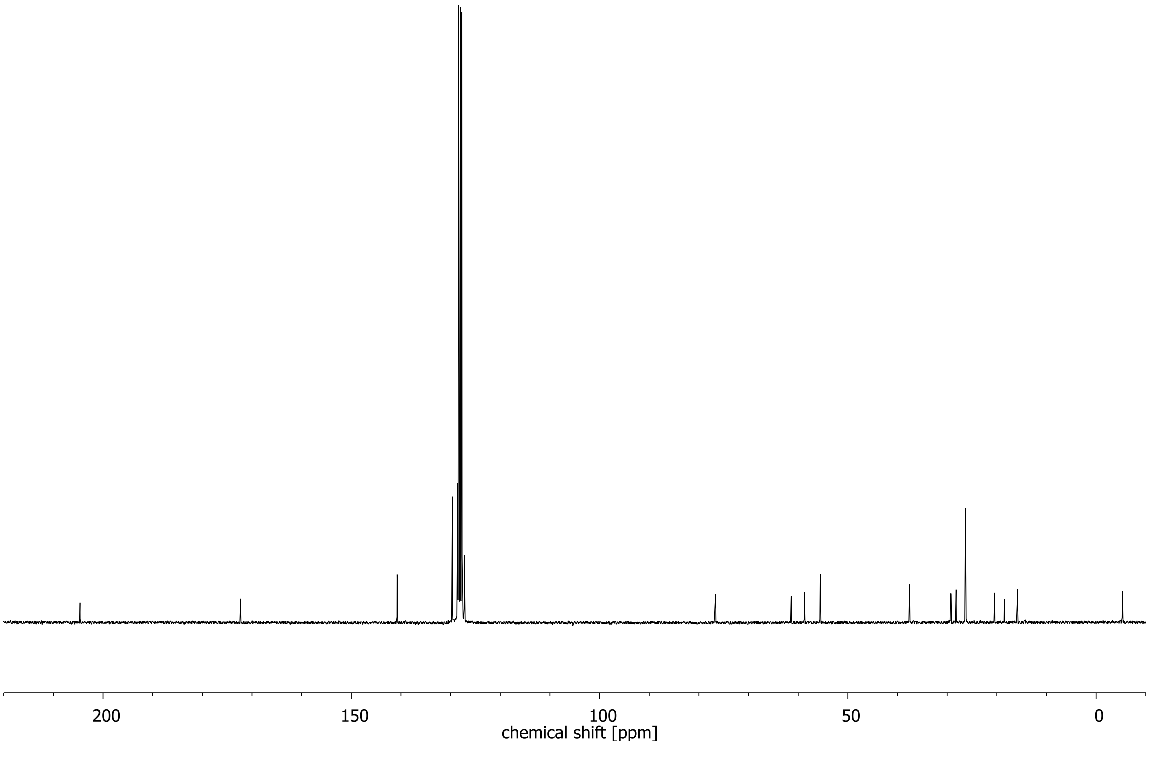

^{13}C NMR spectrum of **6** (76 MHz, C_6D_6)

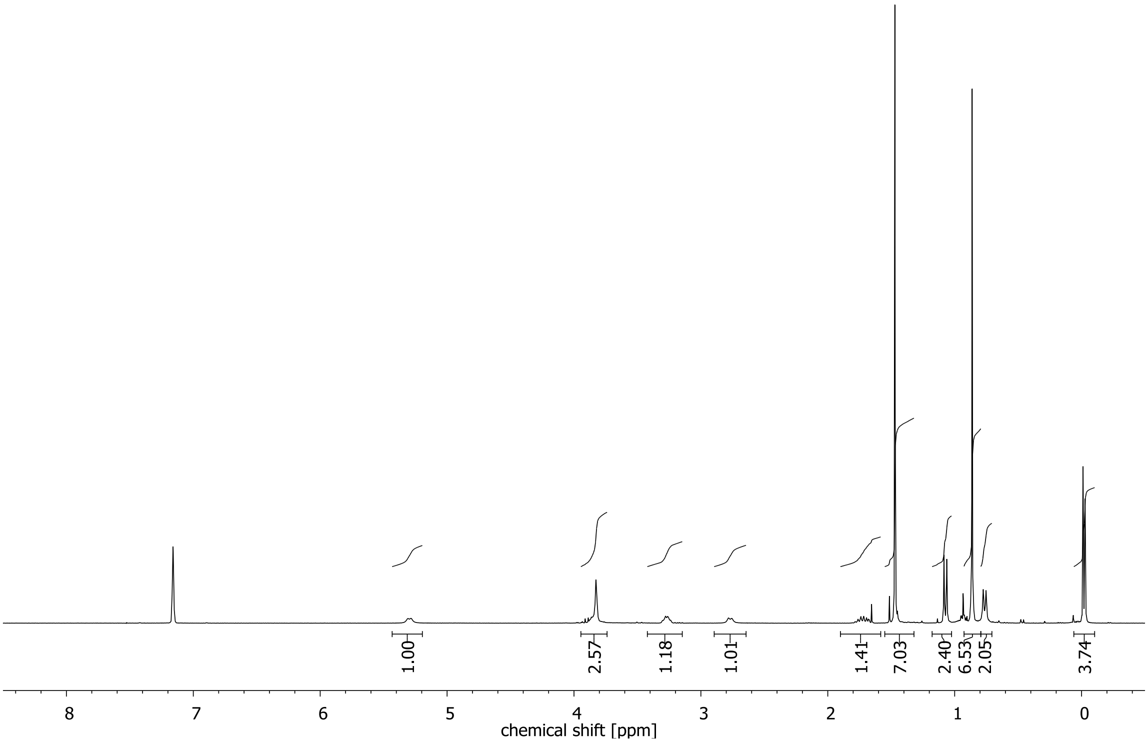

^{1}H NMR spectrum of **7** (300 MHz, C_6D_6)

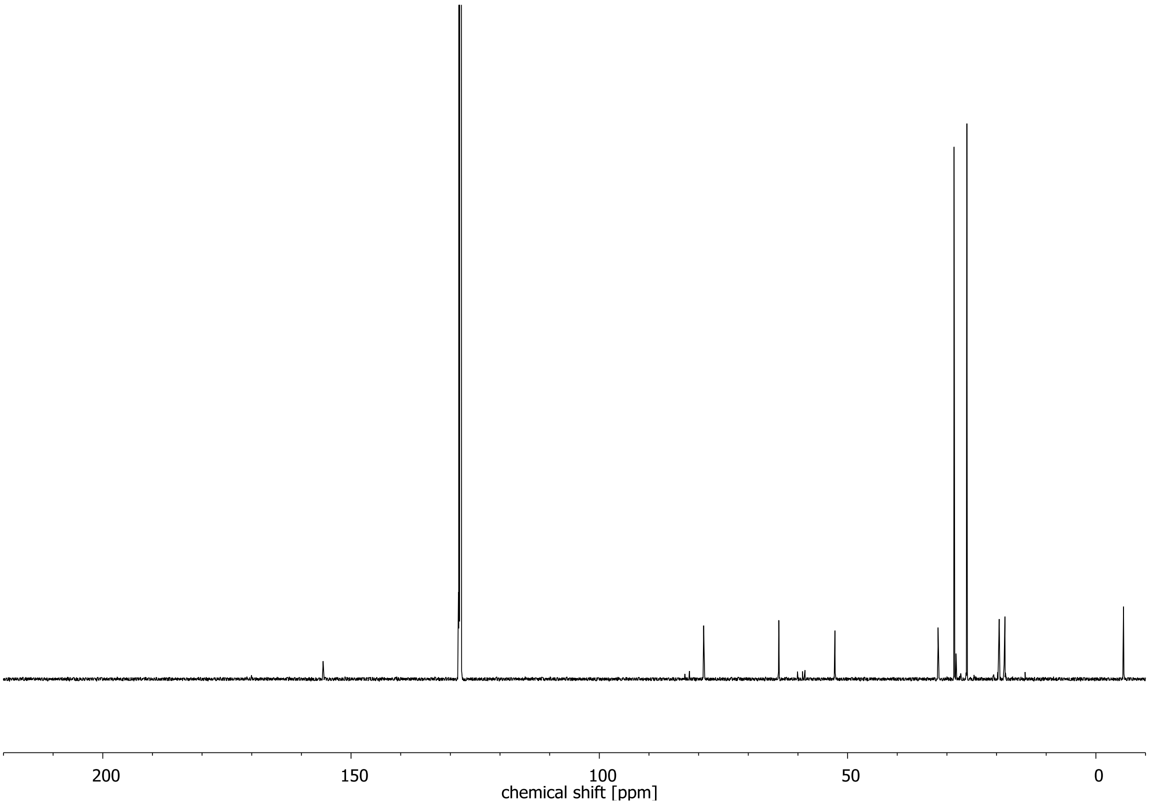

^{13}C NMR spectrum of **7** (126 MHz, C_6D_6)

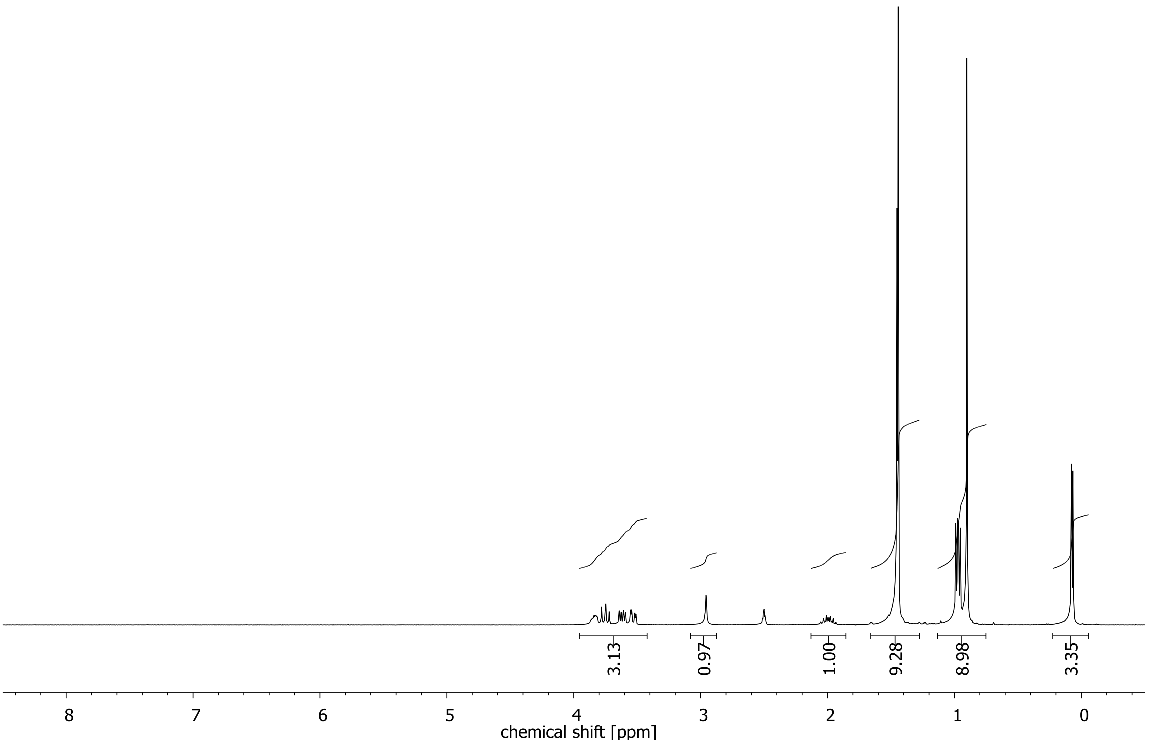

^{1}H NMR spectrum of **8** (300 MHz, DMSO-d_6)

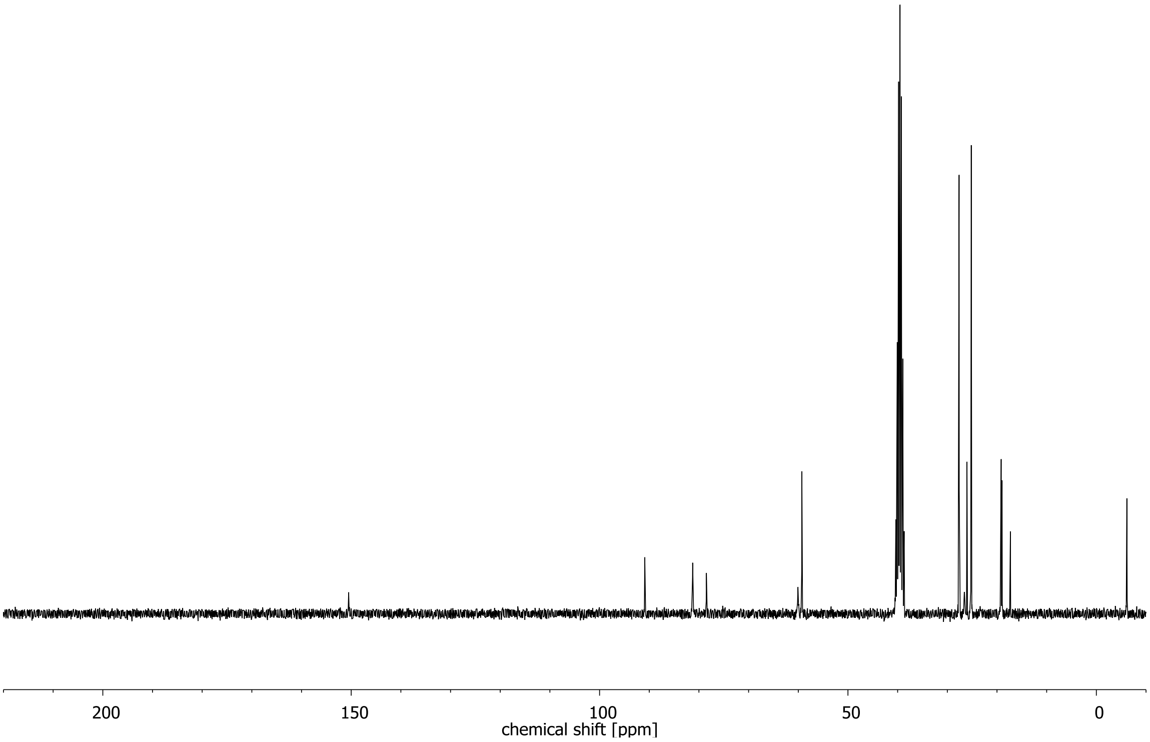

^{13}C NMR spectrum of **8** (76 MHz, DMSO-d_6)

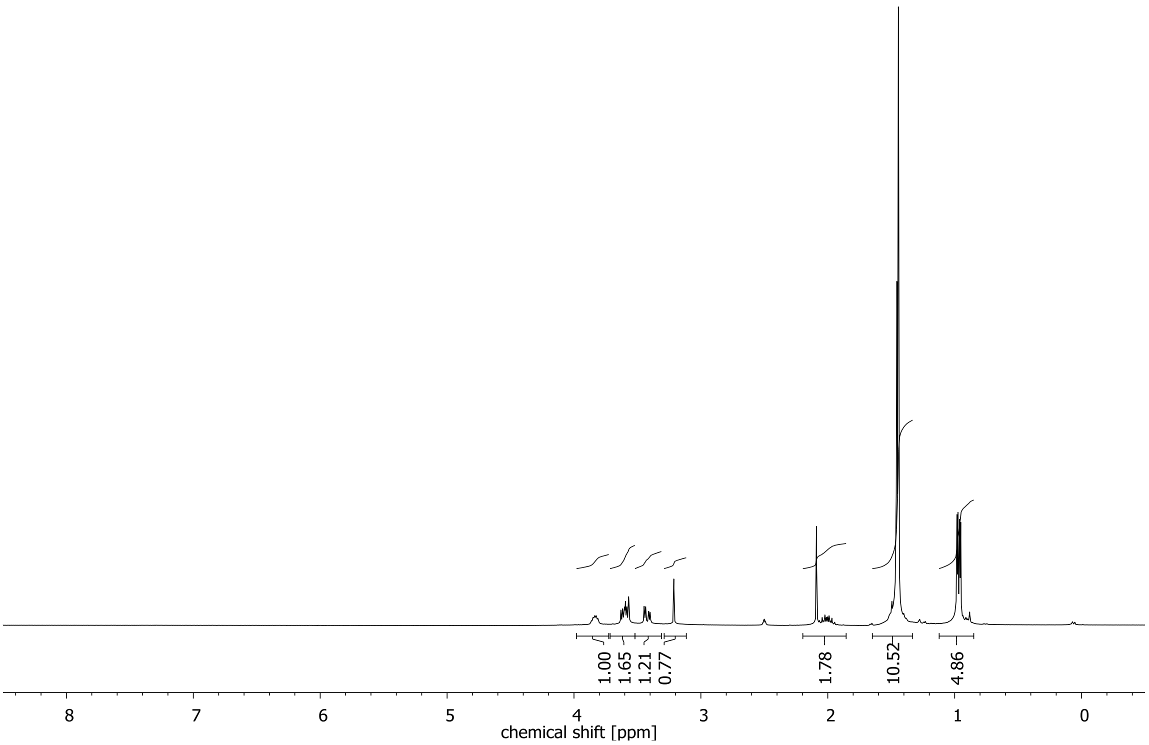

^{1}H NMR spectrum of **9** (300 MHz, DMSO-d_6)

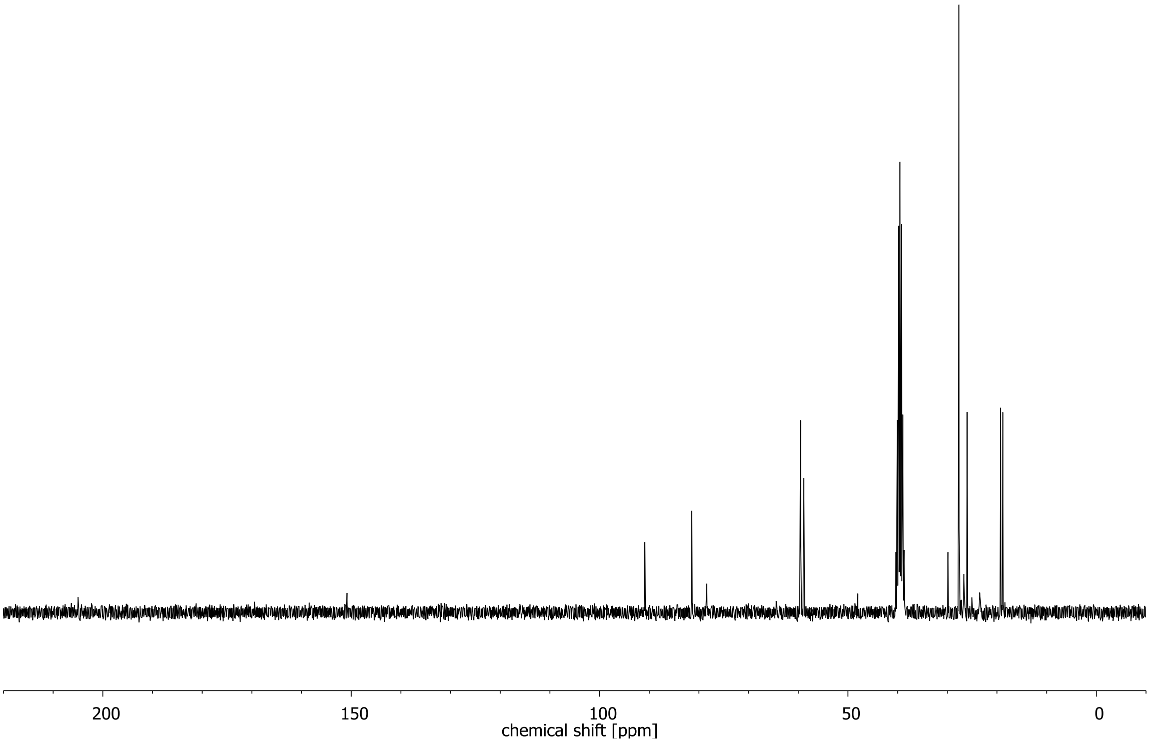

^{13}C NMR spectrum of **9** (76 MHz, DMSO-d_6)

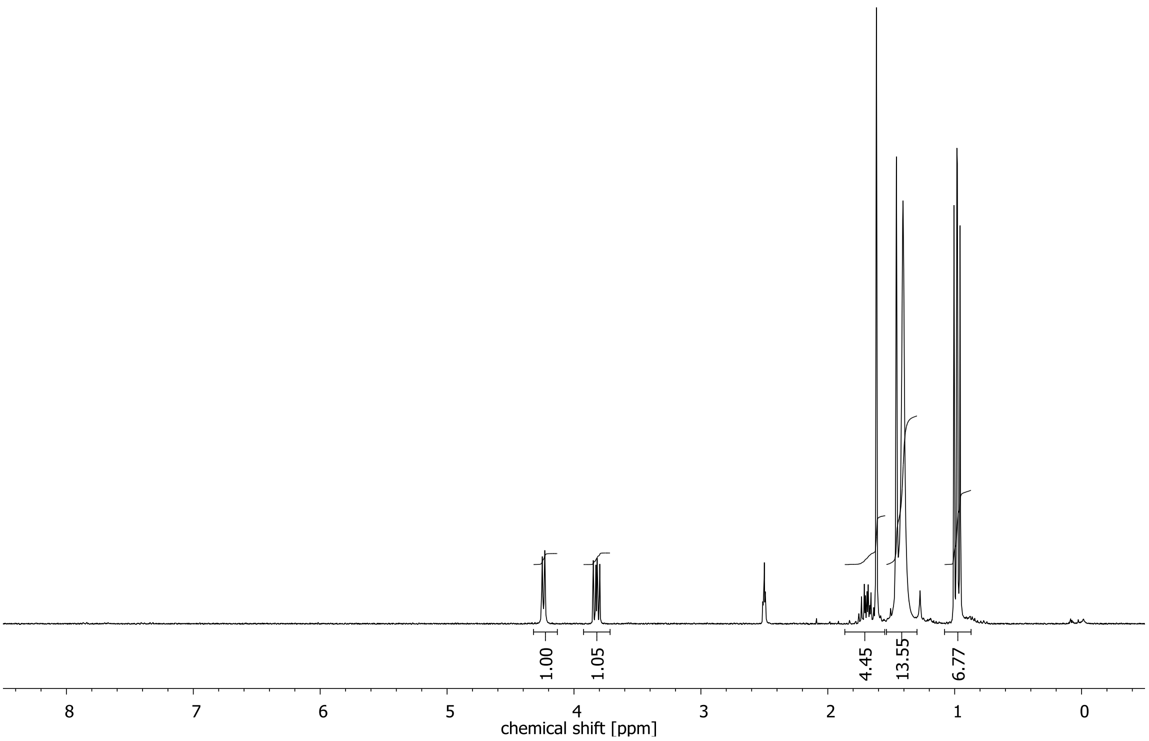

^{1}H NMR spectrum of **11** (300 MHz, DMSO-d_6)

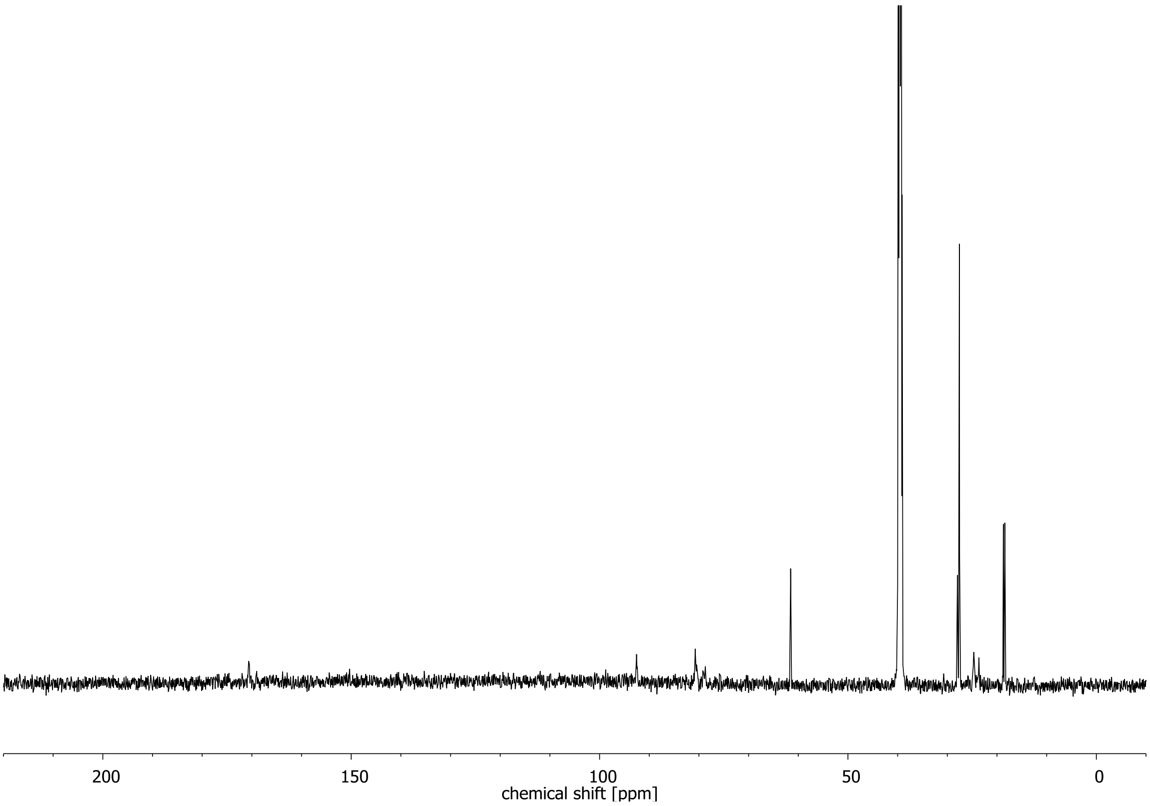

^{13}C NMR spectrum of **11** (151 MHz, DMSO-d_6)

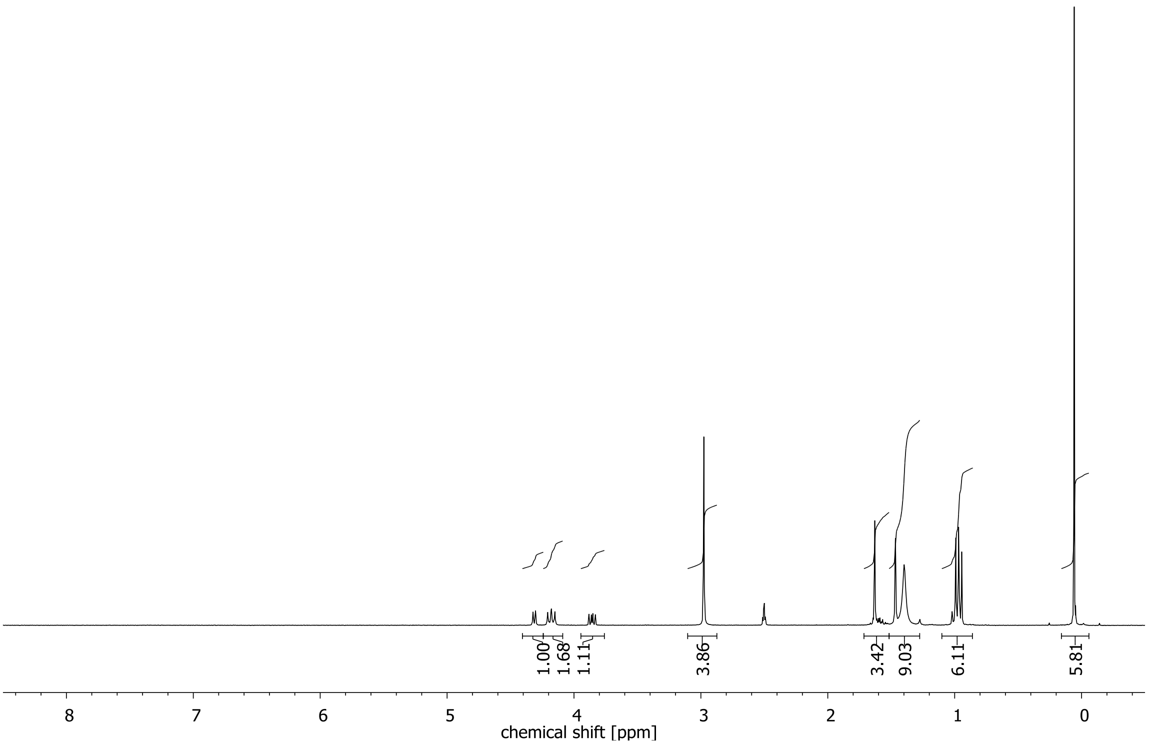

^{1}H NMR spectrum of **12a** (300 MHz, DMSO-d_6)

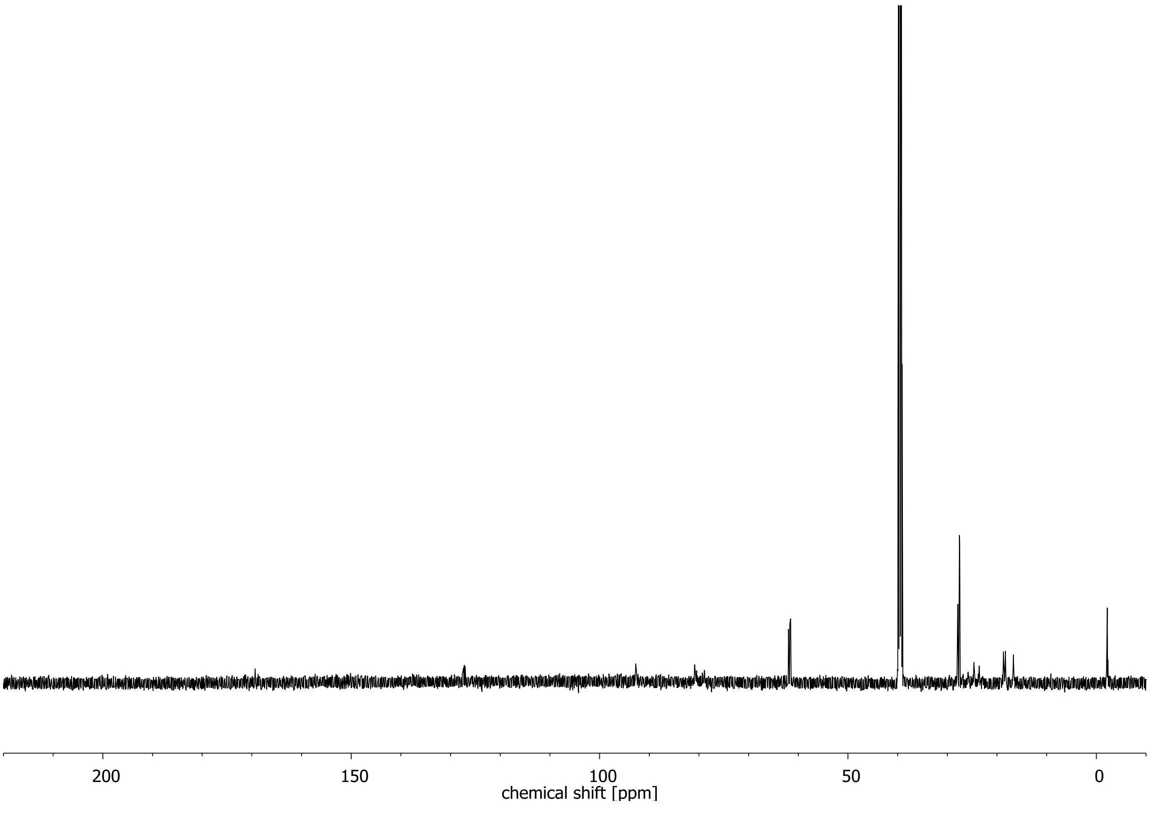

^{13}C NMR spectrum of **12a** (151 MHz, DMSO-d_6)

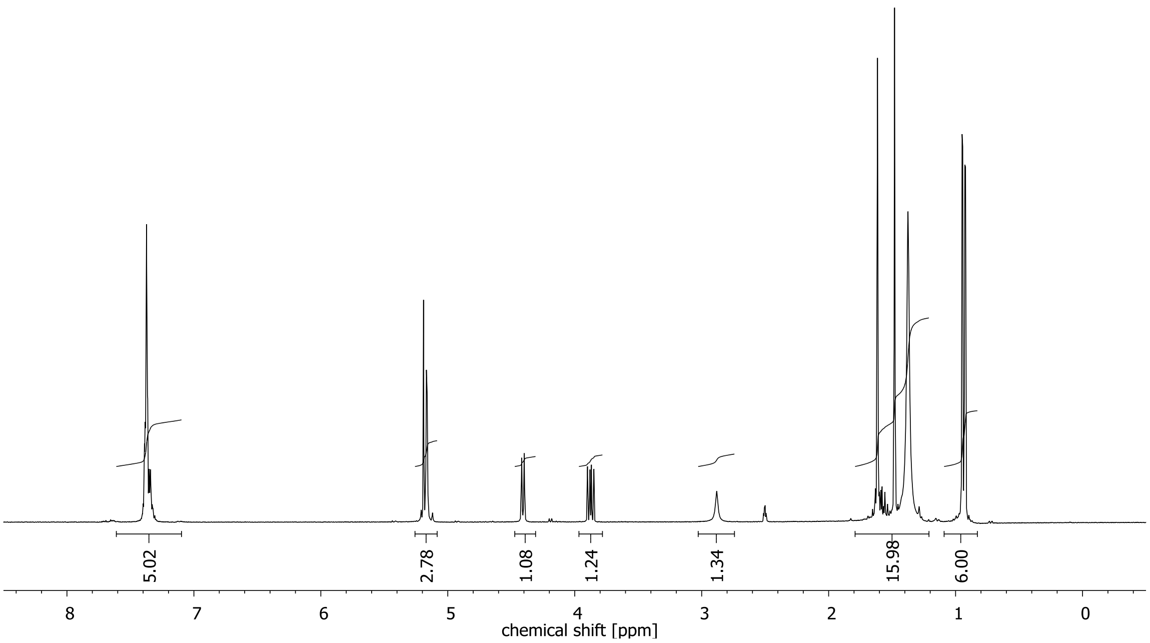

^{1}H NMR spectrum of **12b** (300 MHz, DMSO-d_6, 120 °C)

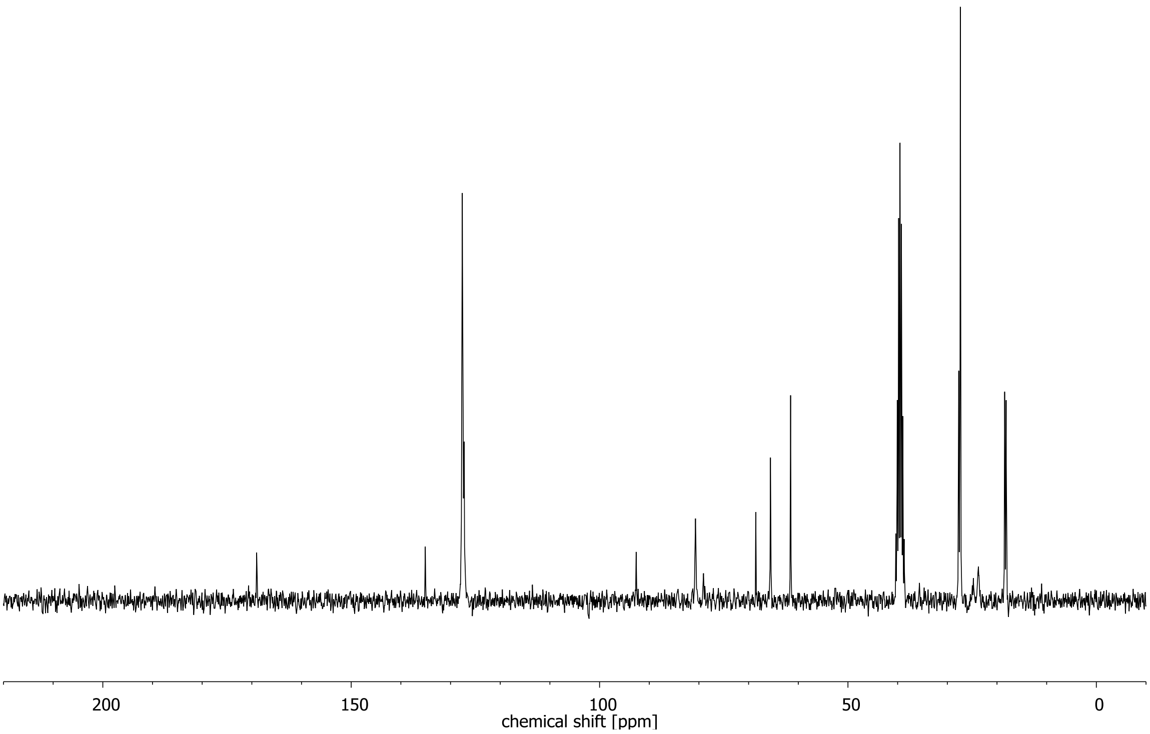

^{13}C NMR spectrum of **12b** (75 MHz, DMSO-d_6, 120 °C)

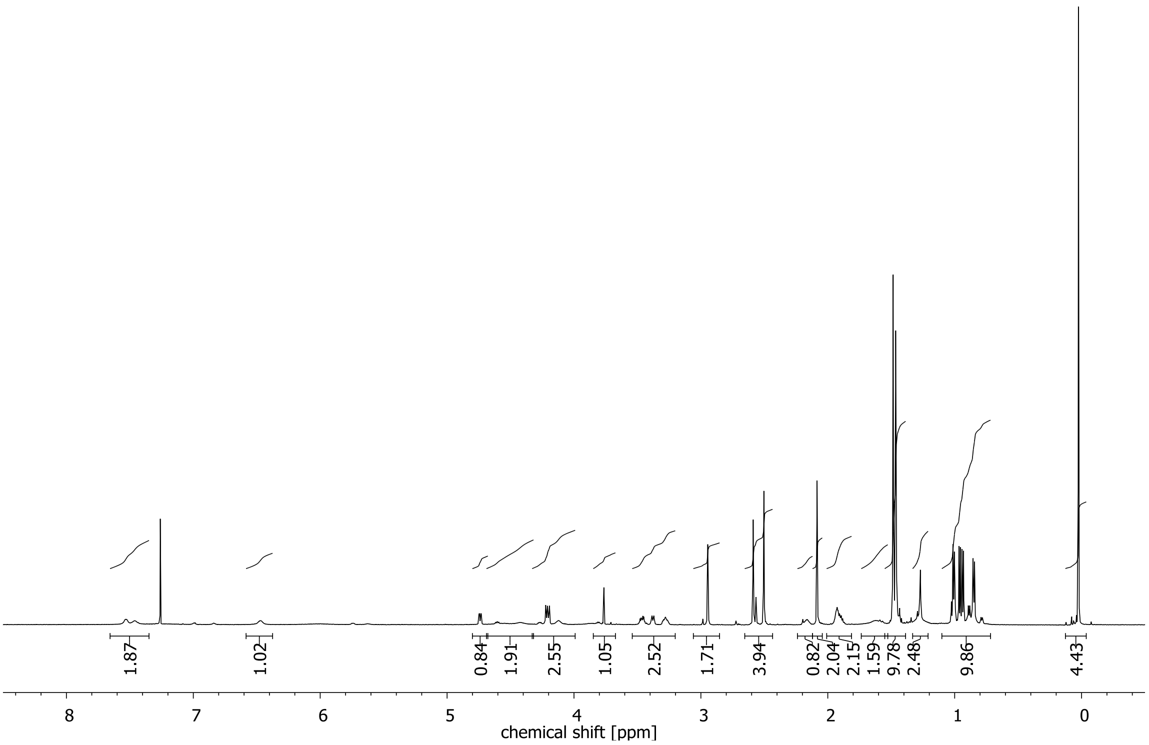

^{1}H NMR spectrum of **15a** (600 MHz, $CDCl_3$, 50 °C)

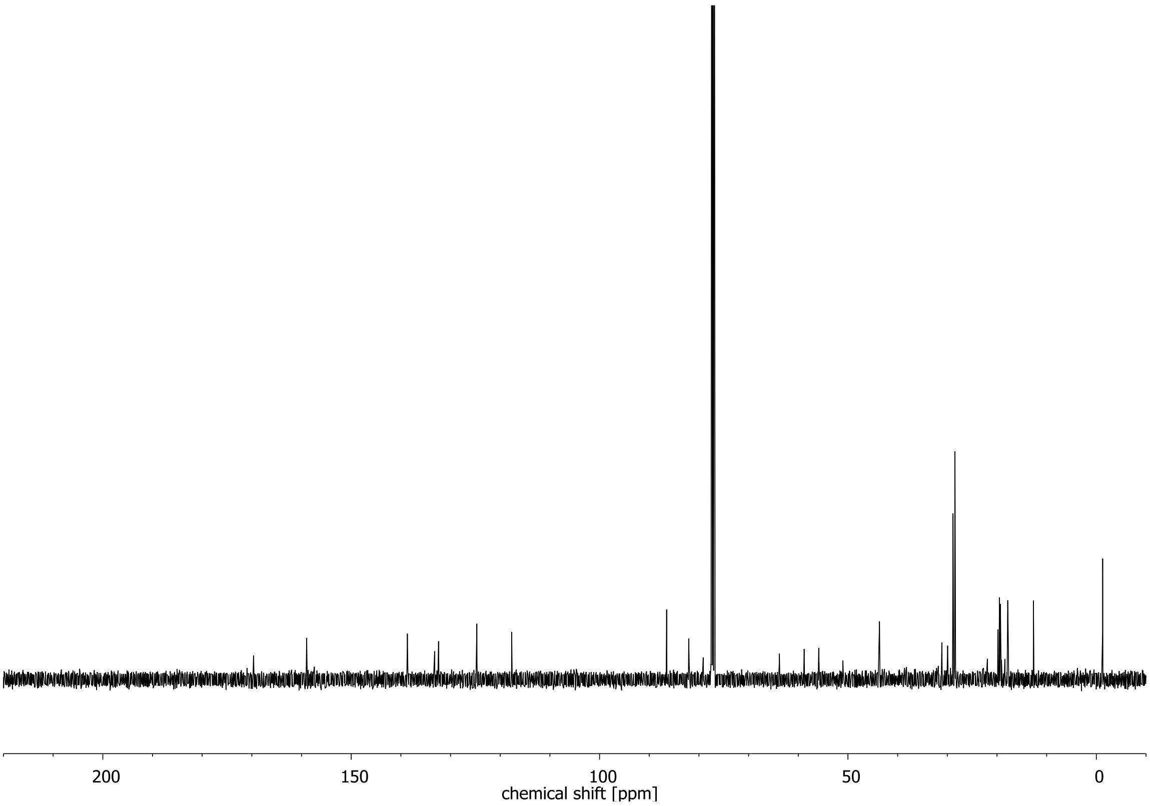

^{13}C NMR spectrum of **15a** (126 MHz, $CDCl_3$, 50 °C)

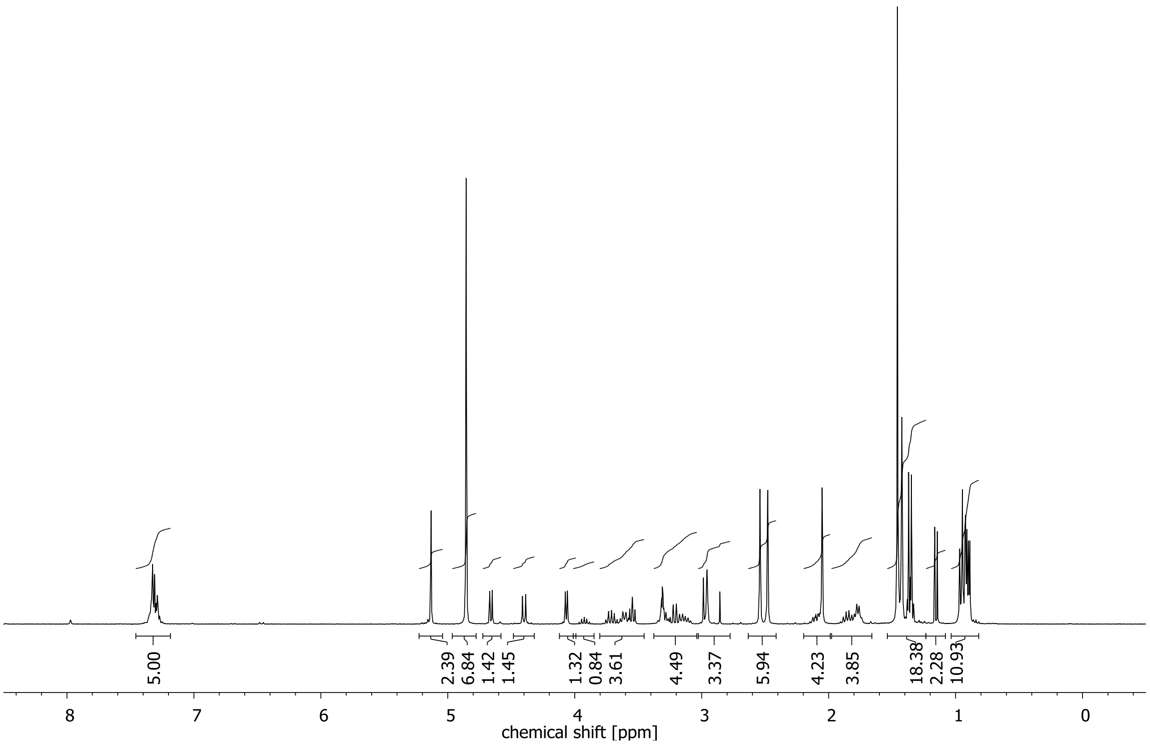

^{1}H NMR spectrum of **15b** (300 MHz, CD_3OD)

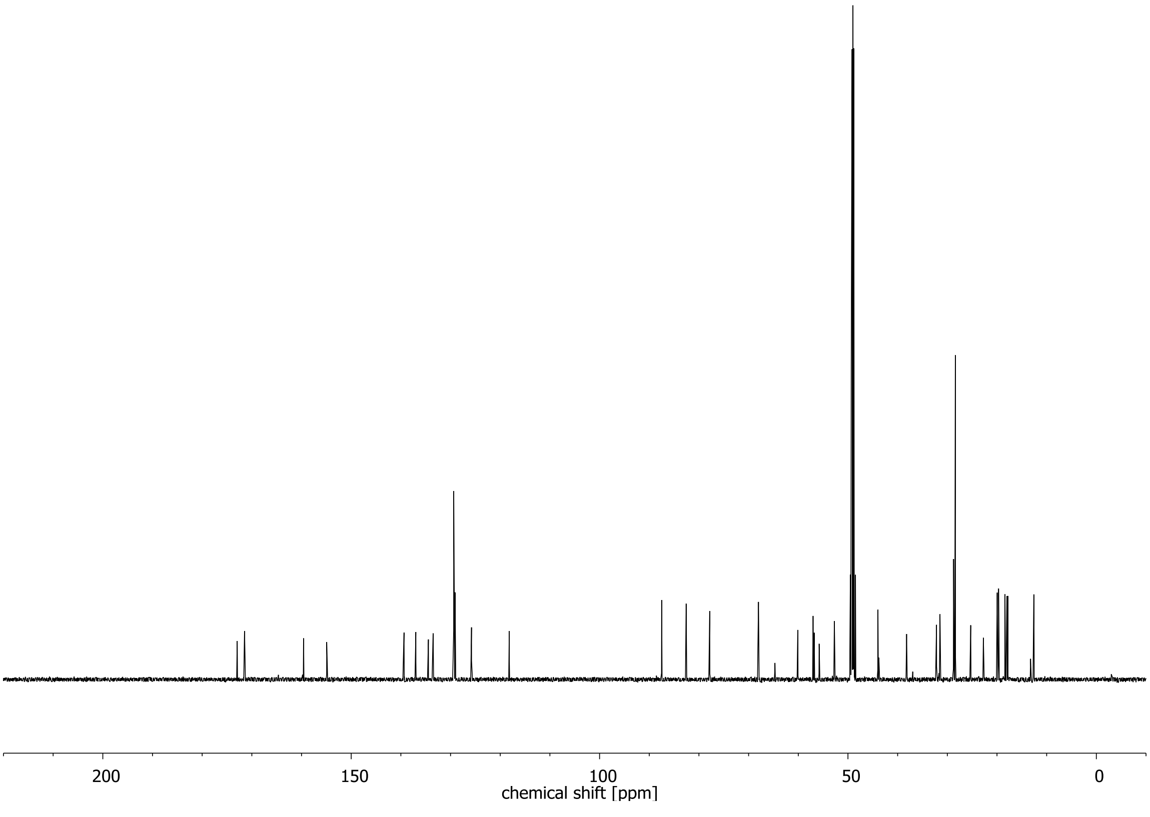

^{13}C NMR spectrum of **15b** (126 MHz, CD_3OD)

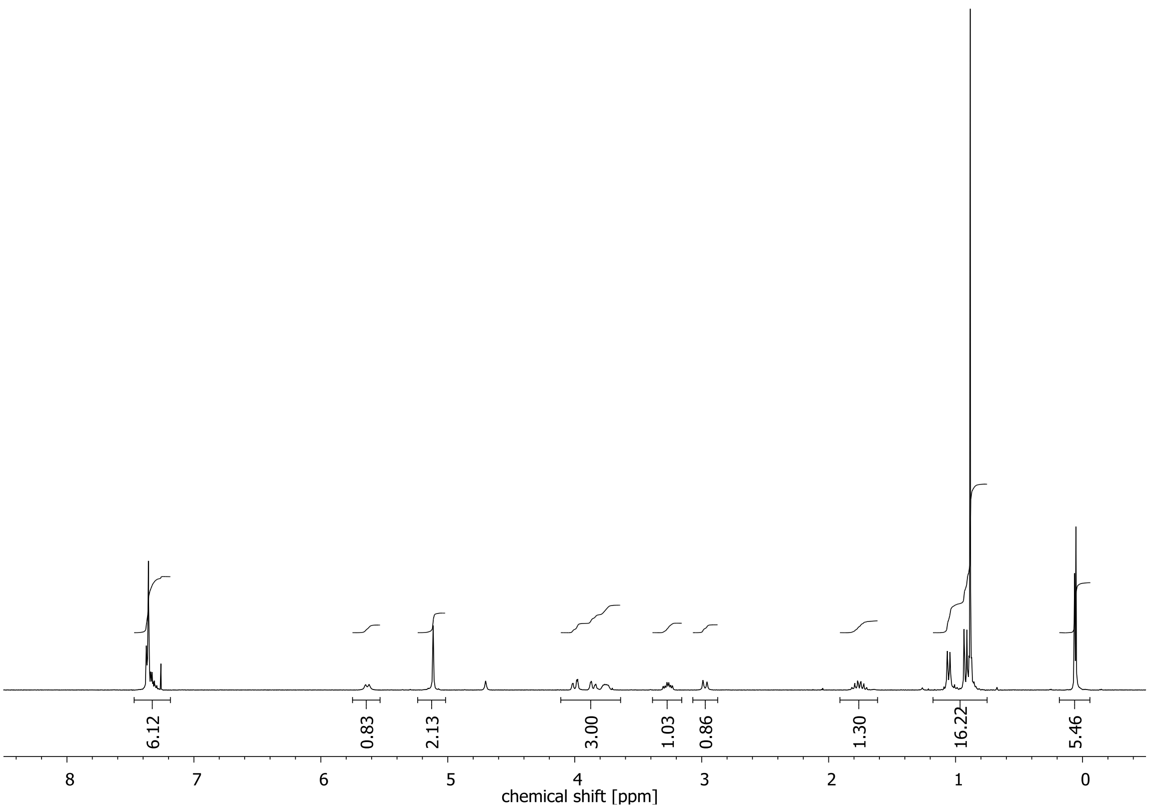

^{1}H NMR spectrum of **16** (300 MHz, $CDCl_3$)

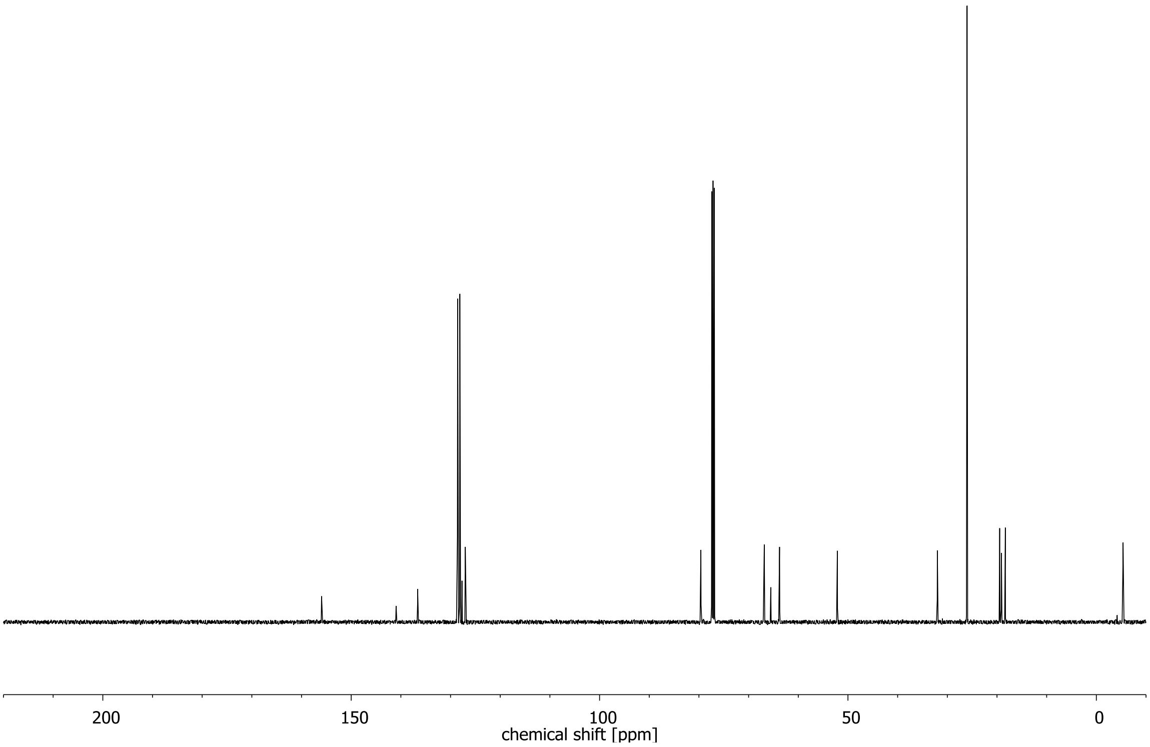

^{13}C NMR spectrum of **16** (126 MHz, $CDCl_3$)

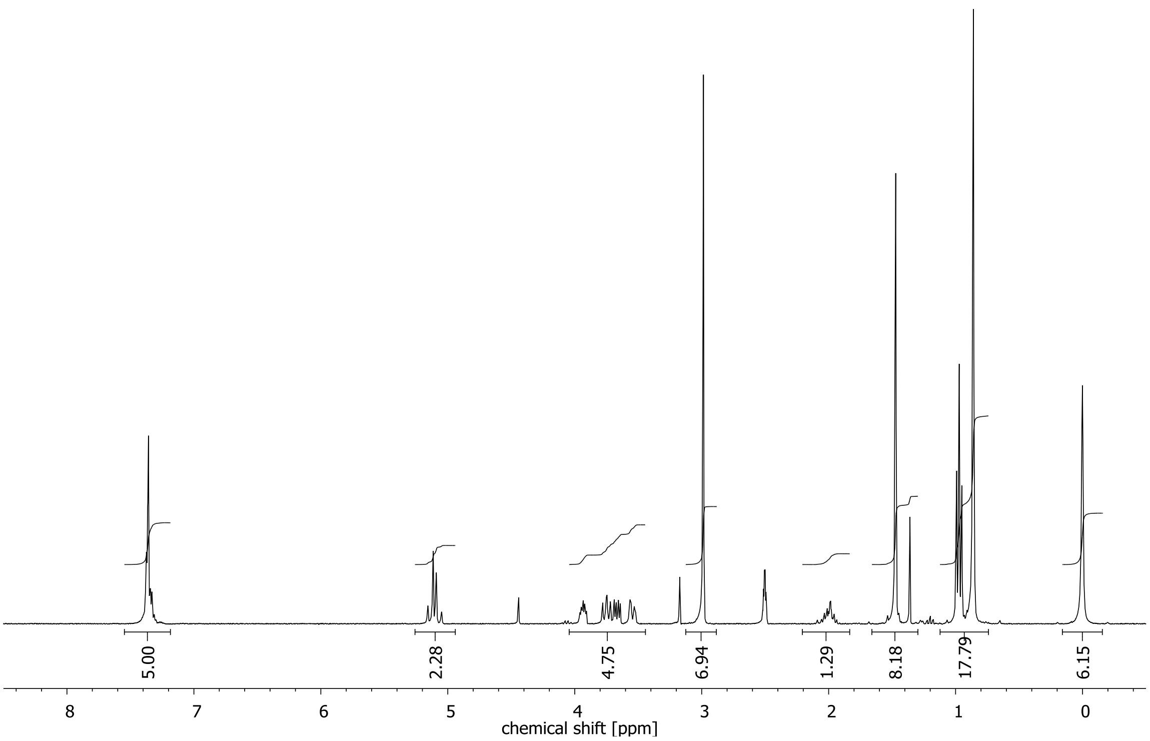

^{1}H NMR spectrum of **17** (300 MHz, DMSO-d_6, 120 °C)

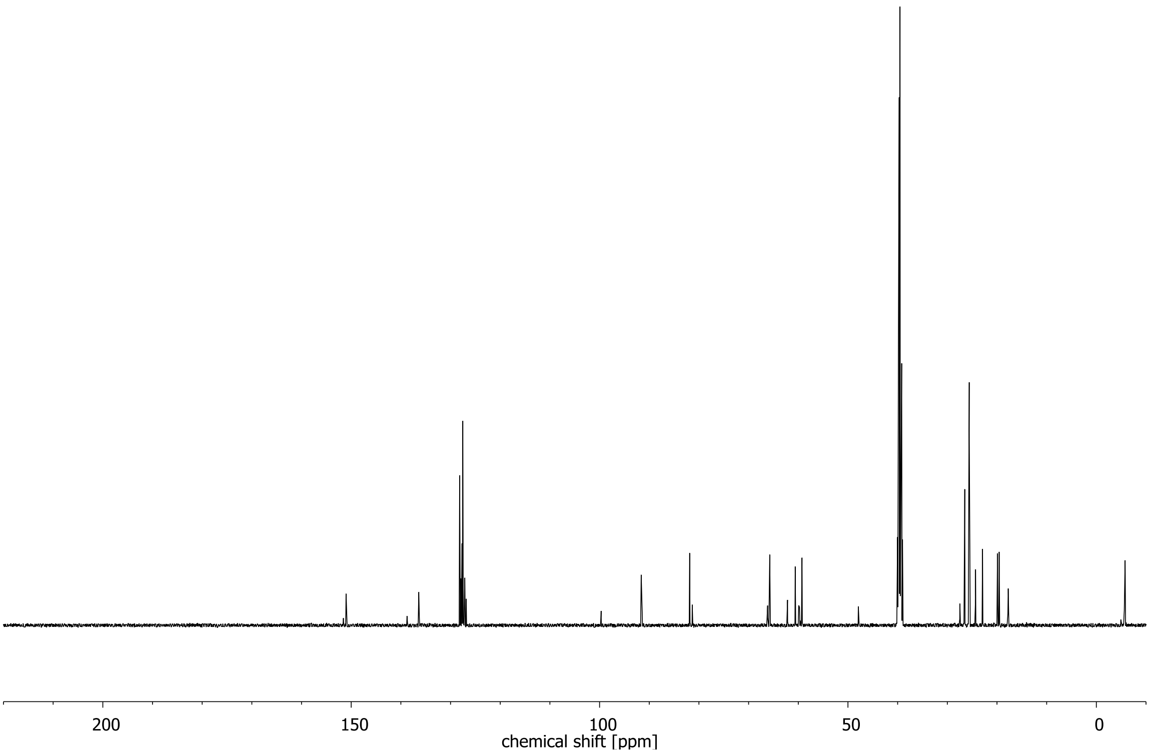

^{13}C NMR spectrum of **17** (126 MHz, DMSO-d_6)

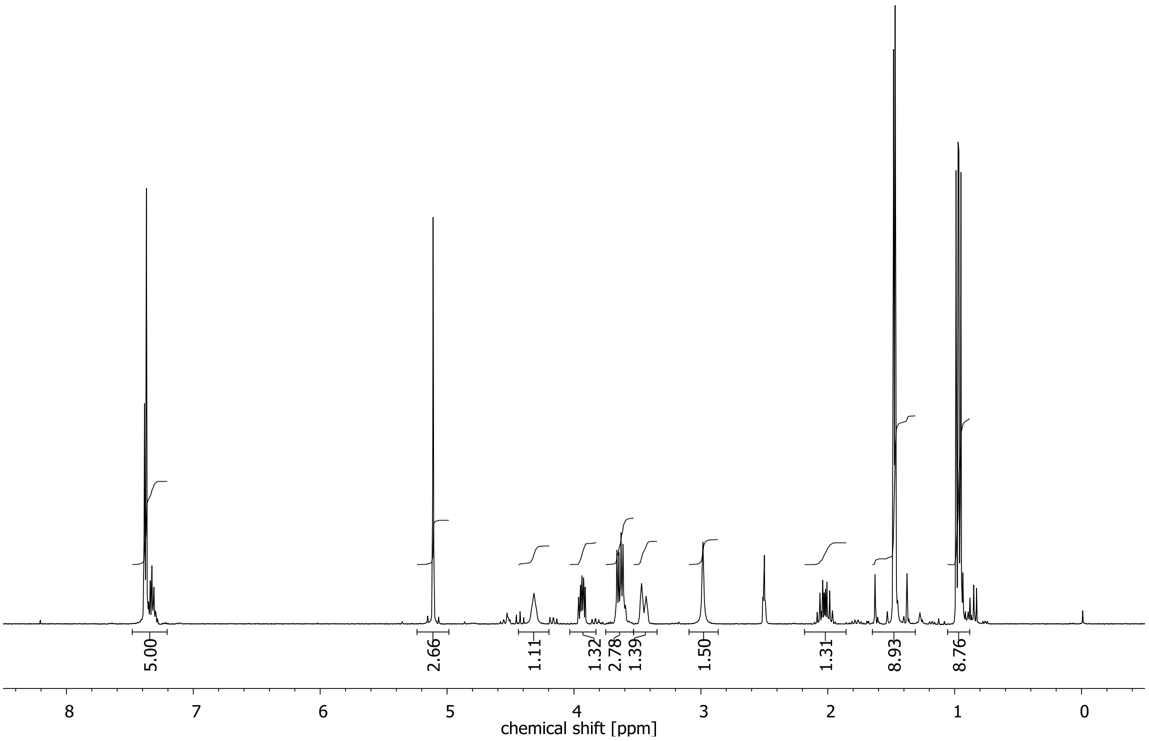

^{1}H NMR spectrum of **18** (300 MHz, DMSO-d_6, 100 °C)

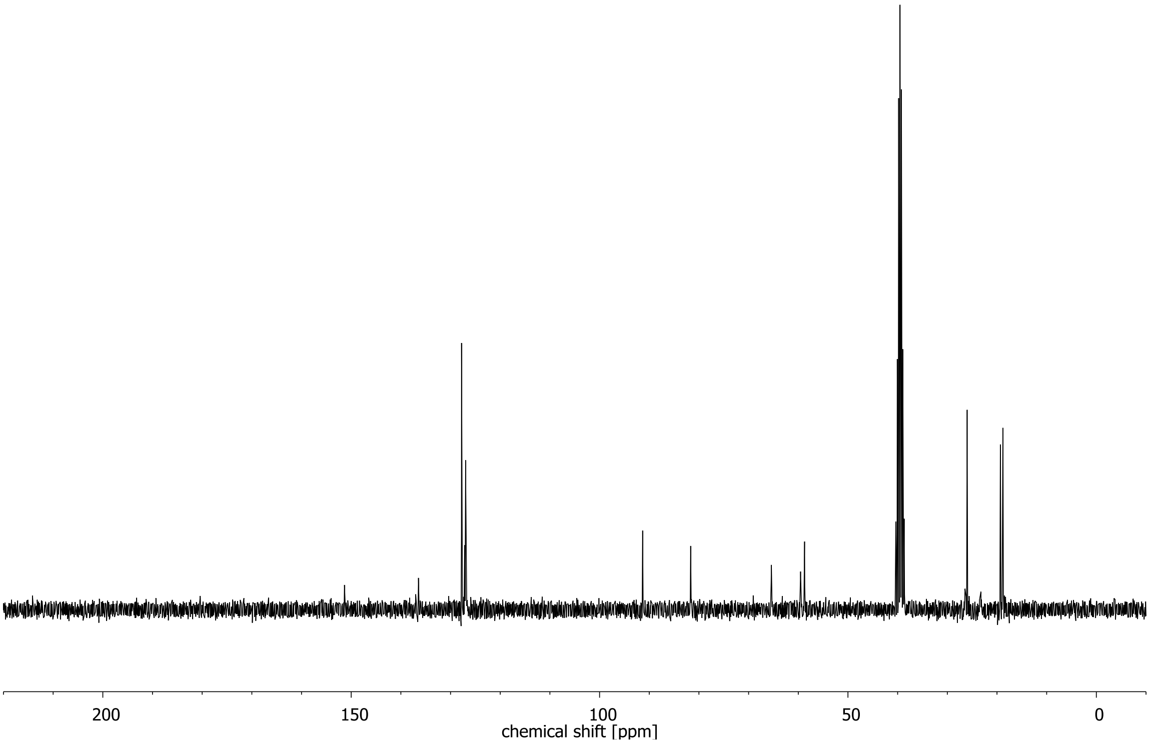

^{13}C NMR spectrum of **18** (76 MHz, DMSO-d_6, 100 °C)

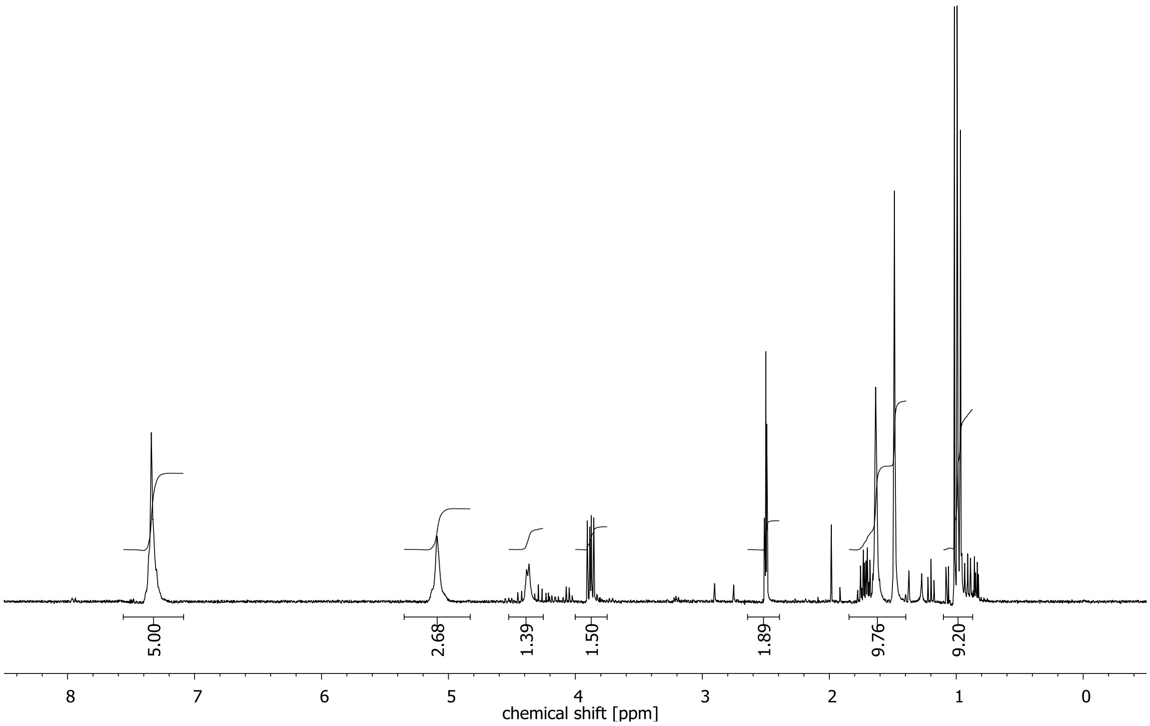

^{1}H NMR spectrum of **19** (300 MHz, DMSO-d_6, 100 °C)

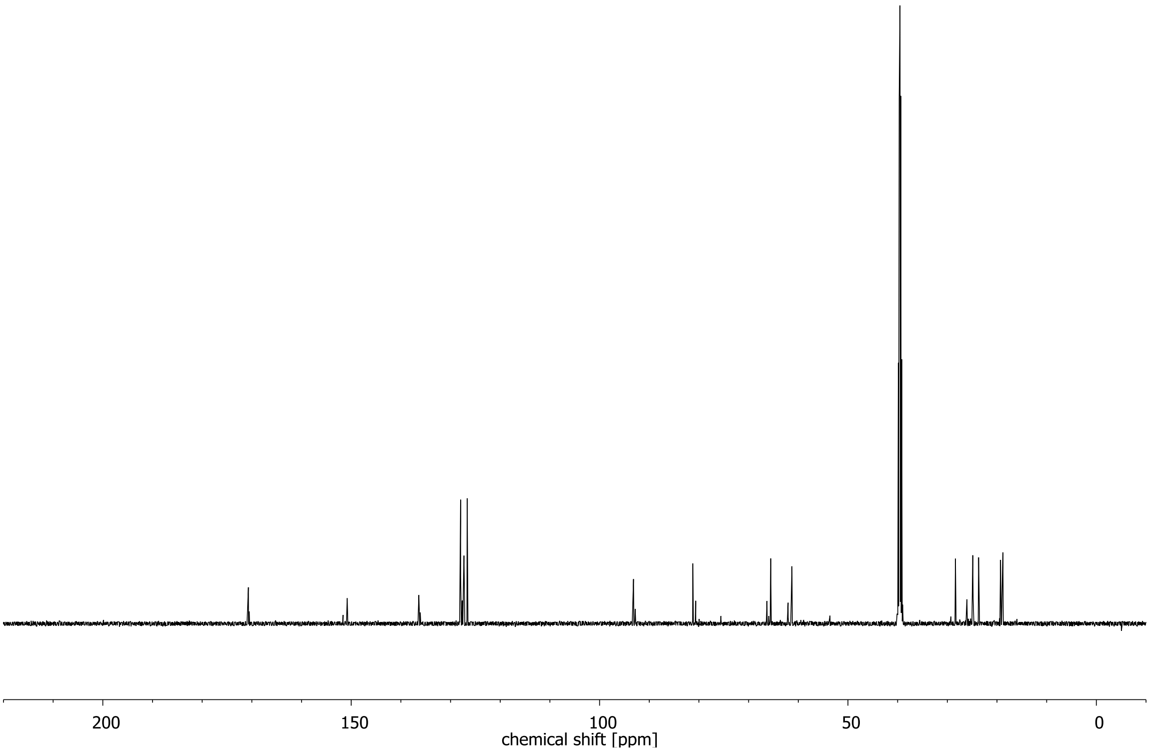

^{13}C NMR spectrum of **19** (126 MHz, DMSO-d_6, 50 °C)

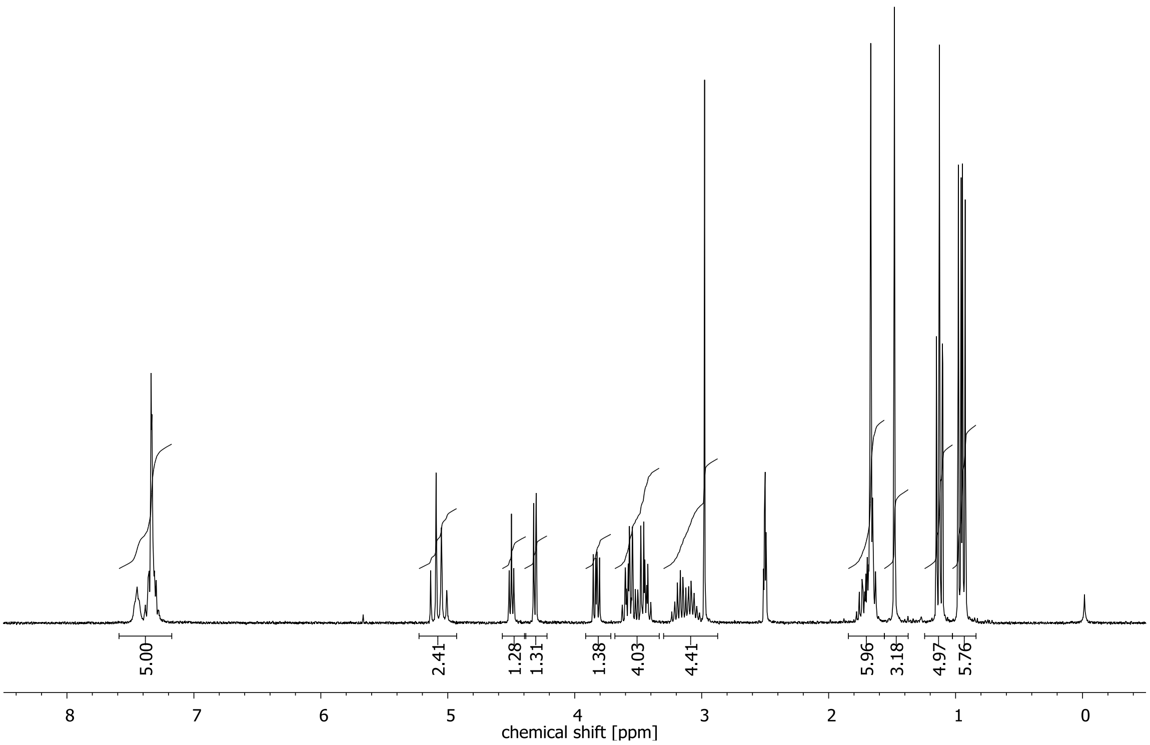

^{1}H NMR spectrum of **21** (300 MHz, DMSO-d_6, 100 °C)

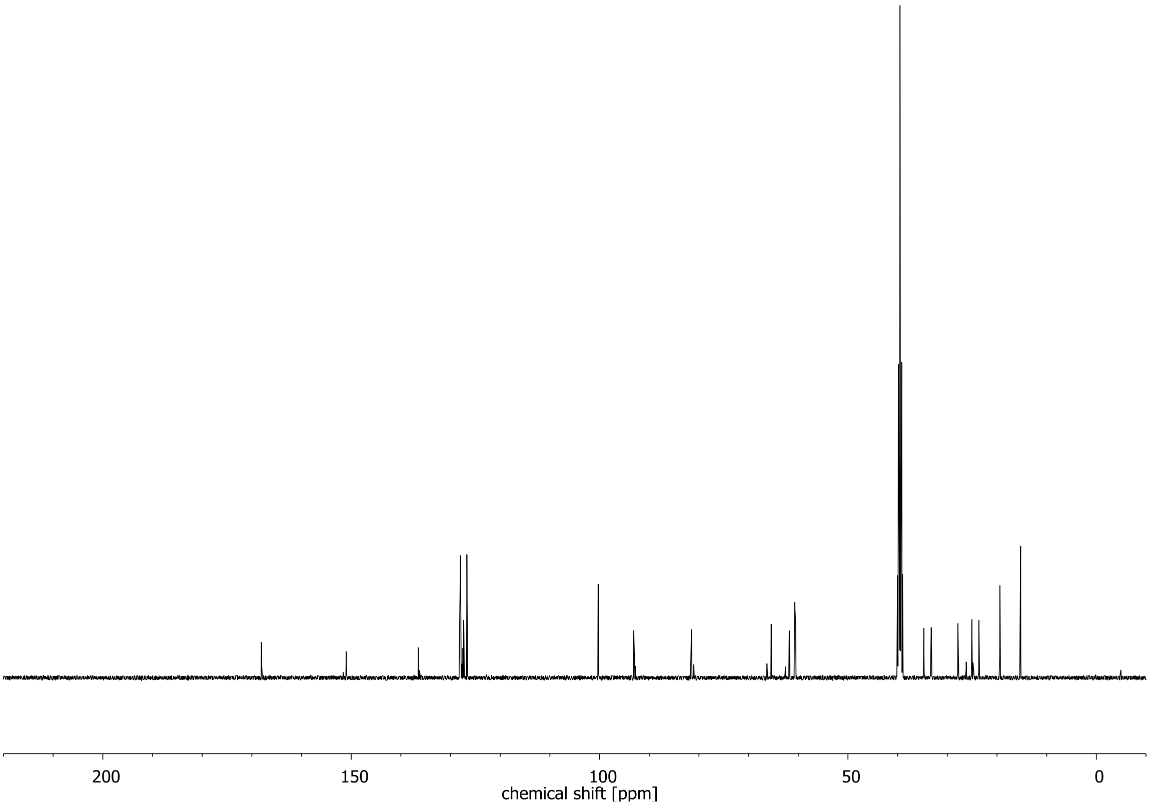

^{13}C NMR spectrum of **21** (126 MHz, DMSO-d_6)

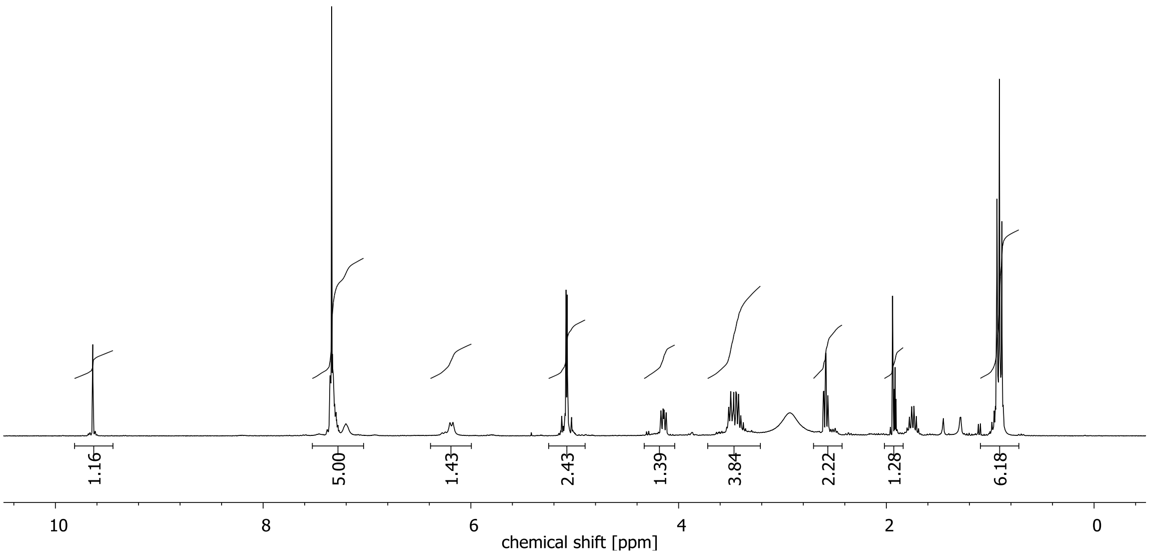

^{1}H NMR spectrum of **22** (300 MHz, CD_3CN, 70 °C)

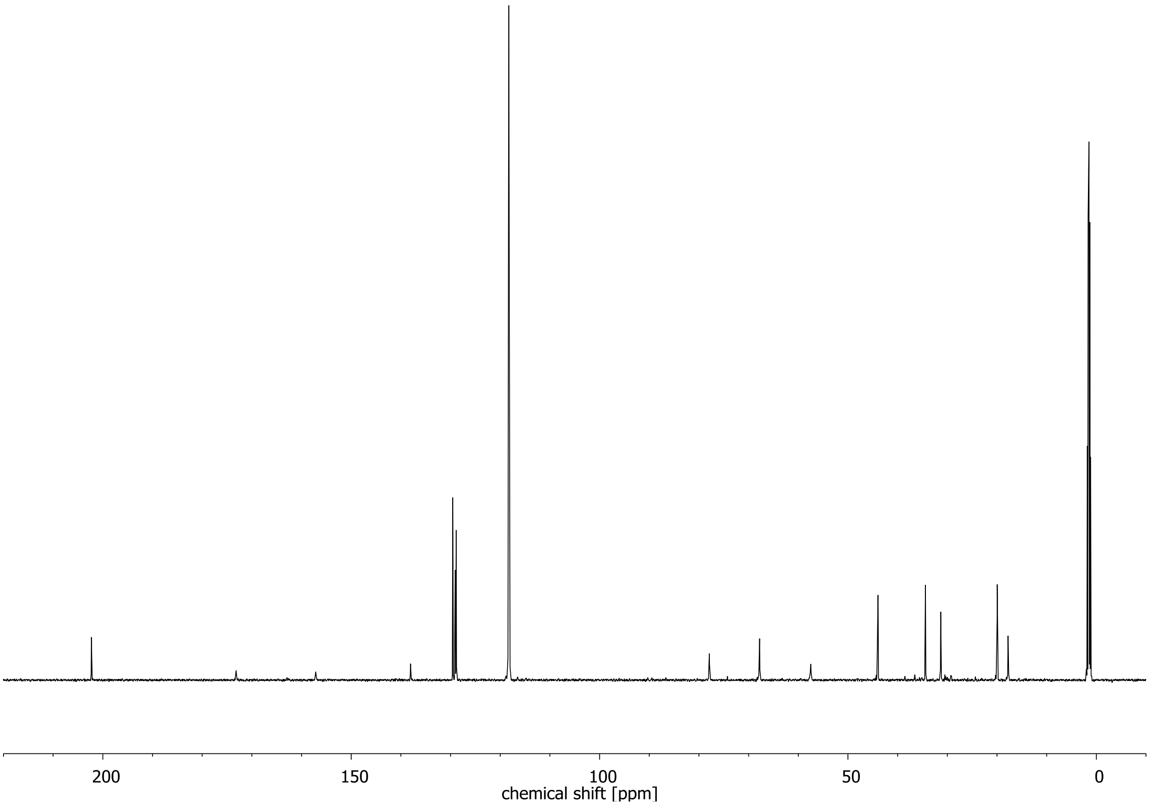

^{13}C NMR spectrum of **22** (126 MHz, CD_3CN, 50 °C)

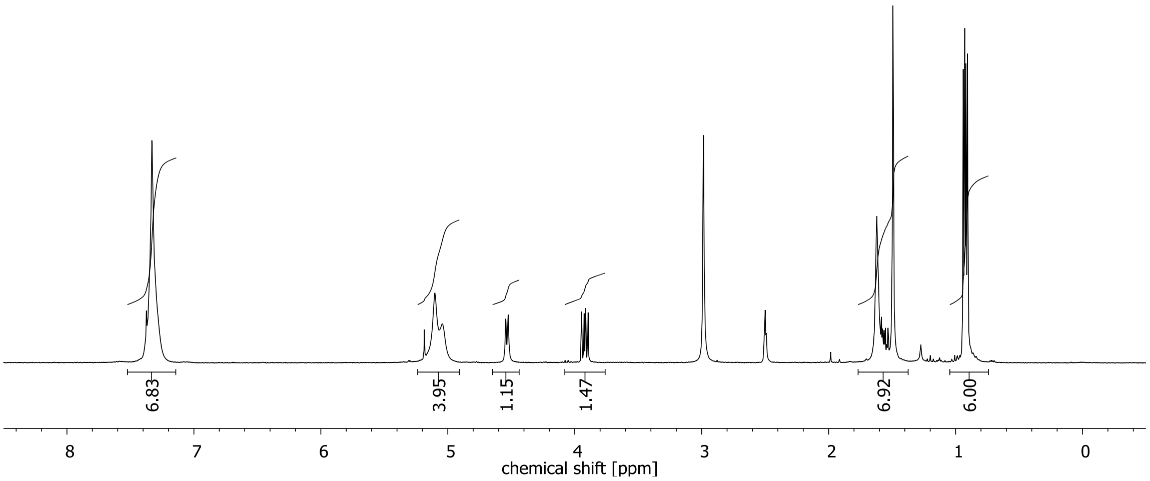

^{1}H NMR spectrum of **23** (300 MHz, DMSO-d_6, 100 °C)

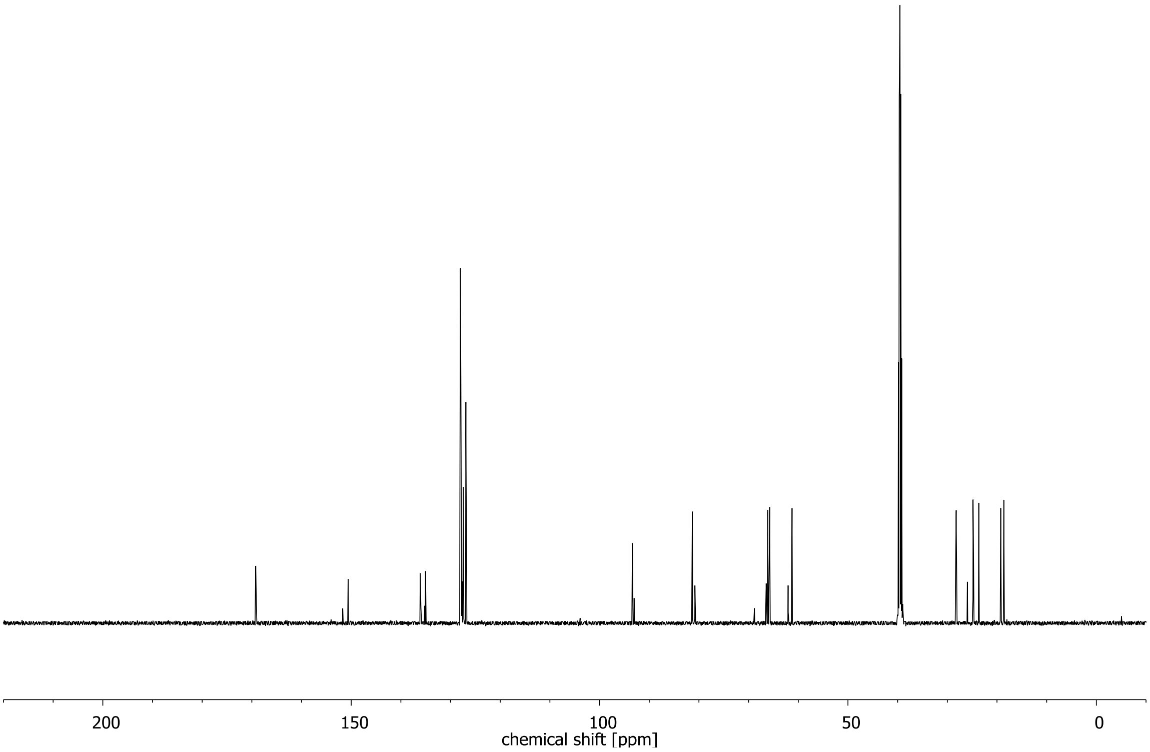

^{13}C NMR spectrum of **23** (126 MHz, DMSO-d_6, 50 °C)

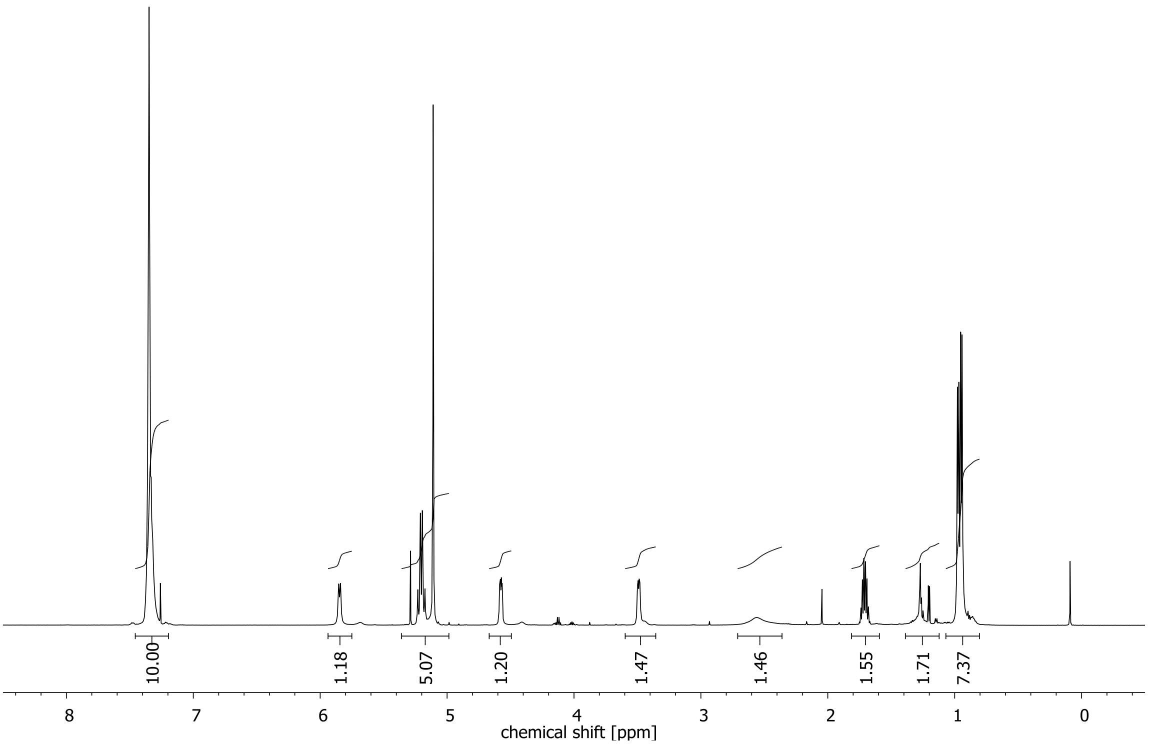

^{1}H NMR spectrum of **24** (600 MHz, $CDCl_3$)

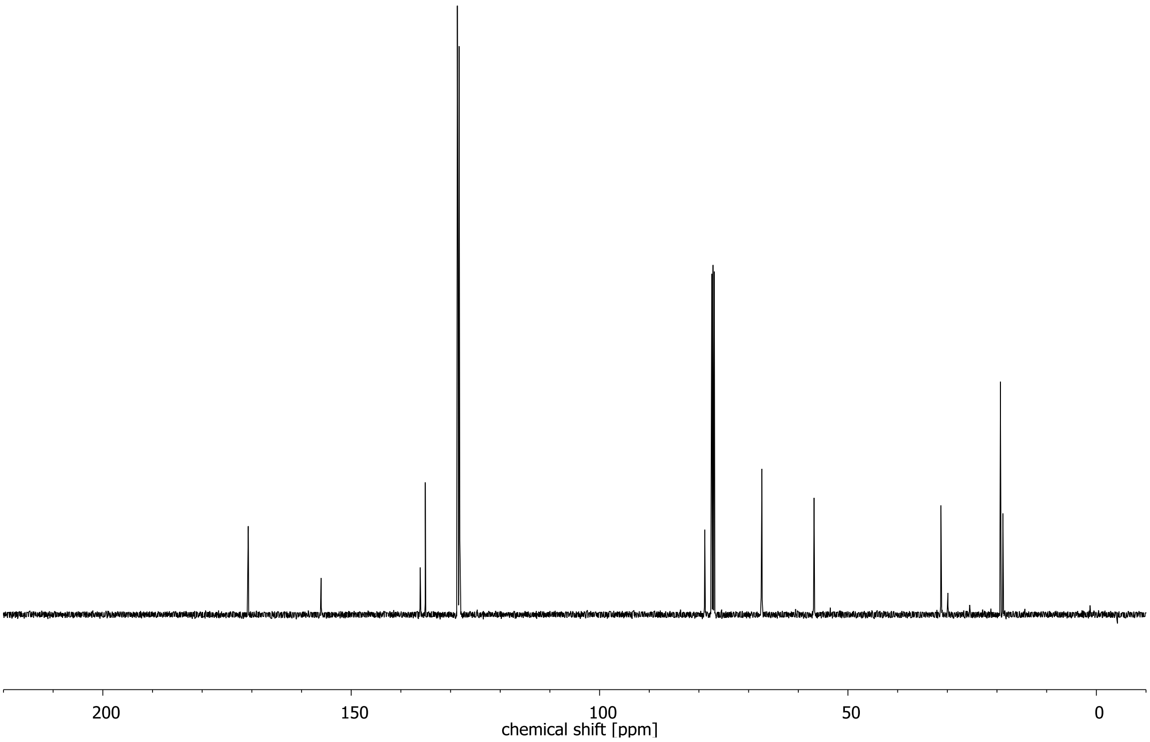

^{13}C NMR spectrum of **24** (126 MHz, $CDCl_3$)

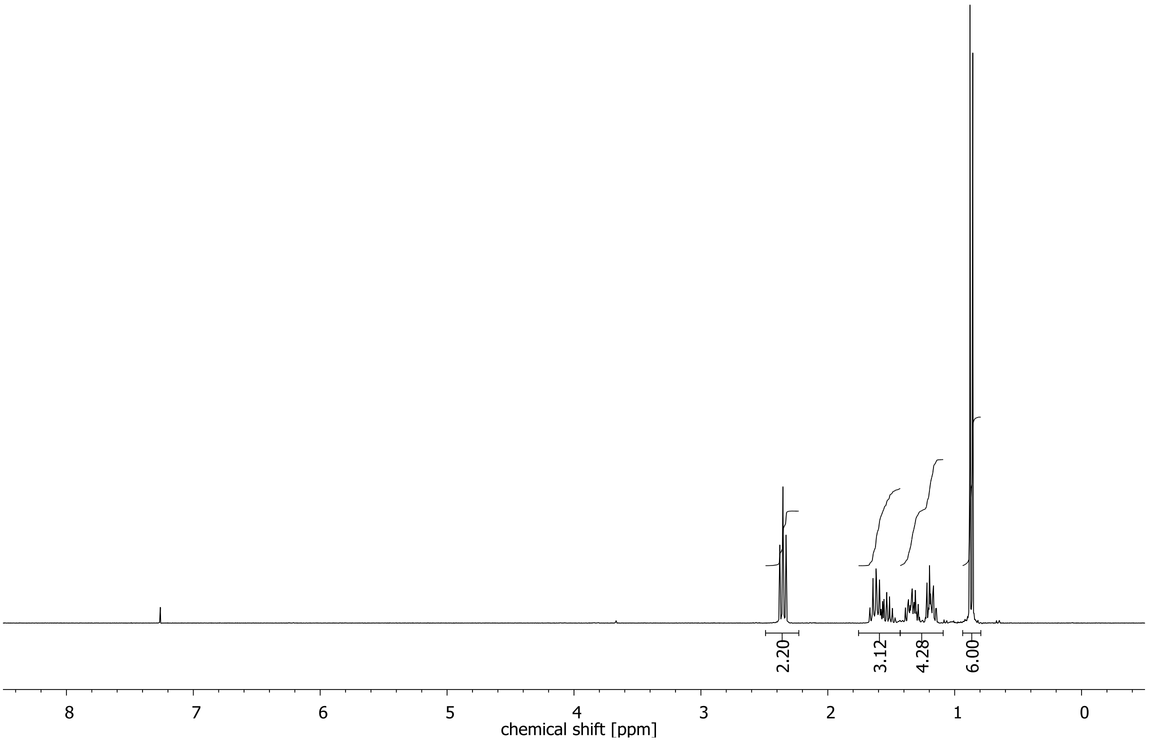

^{1}H NMR spectrum of **26** (300 MHz, $CDCl_3$)

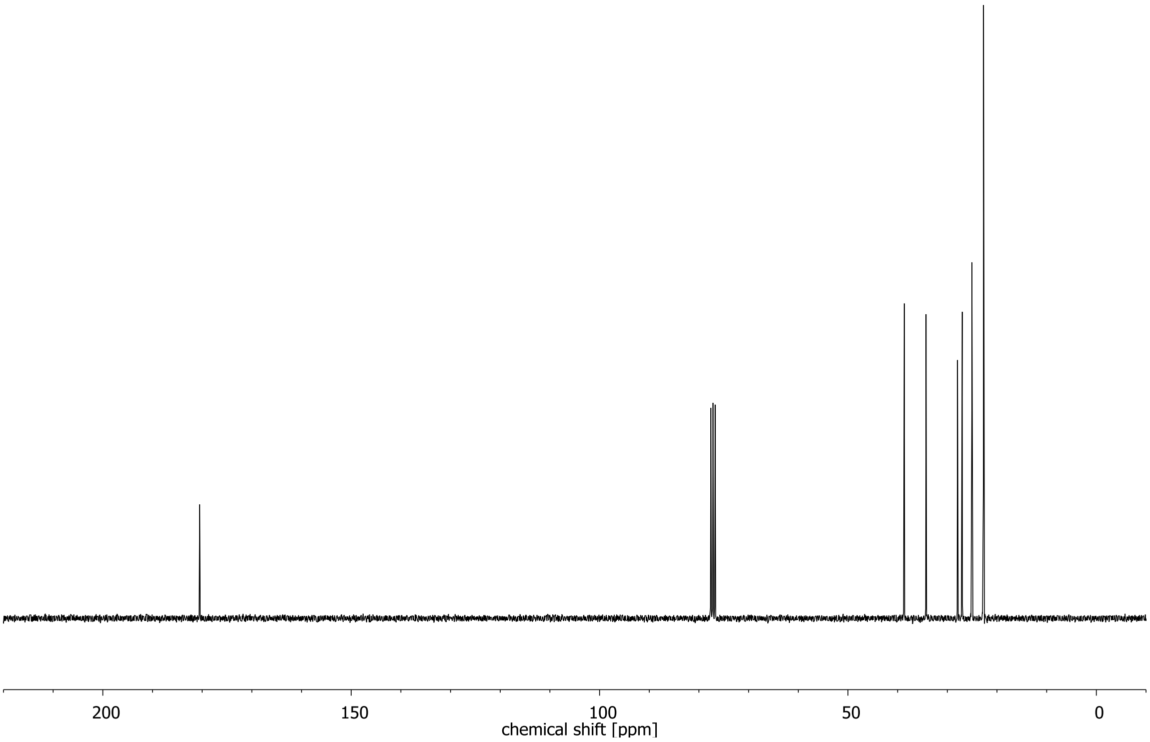

^{13}C NMR spectrum of **26** (75 MHz, $CDCl_3$)

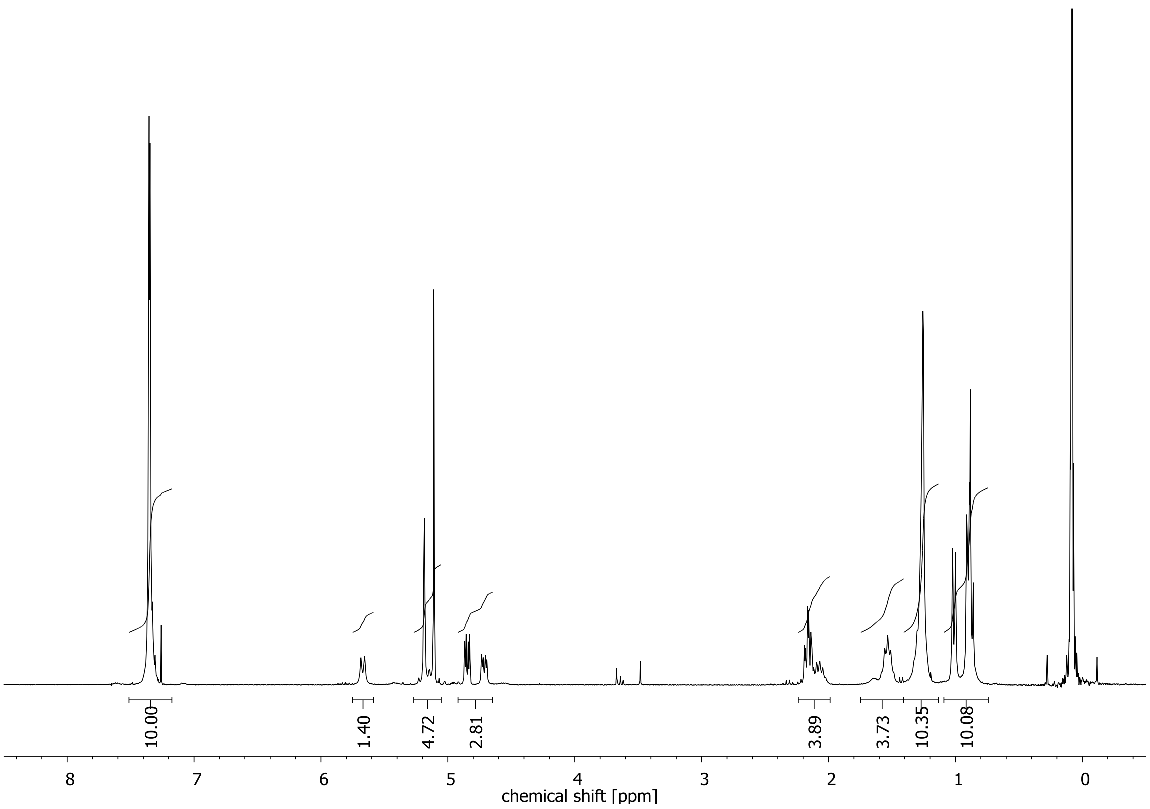

^{1}H NMR spectrum of **27** (300 MHz, $CDCl_3$)

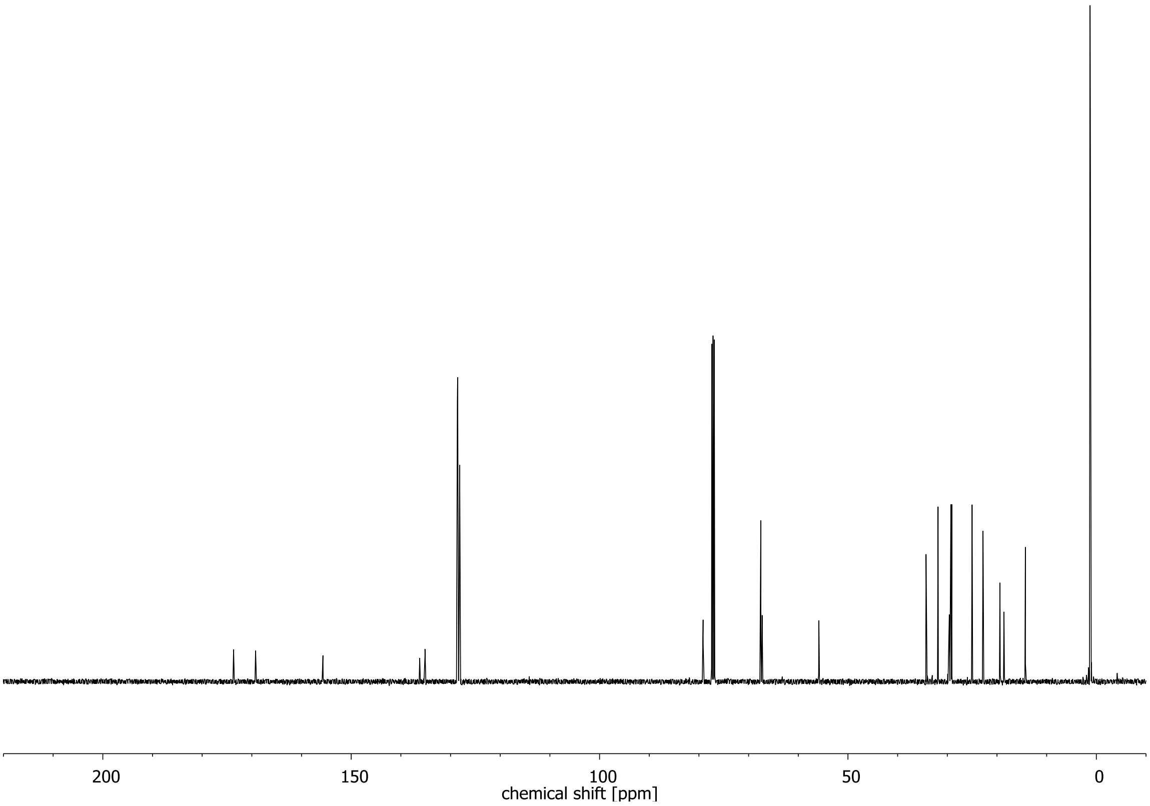

^{13}C NMR spectrum of **27** (126 MHz, $CDCl_3$)

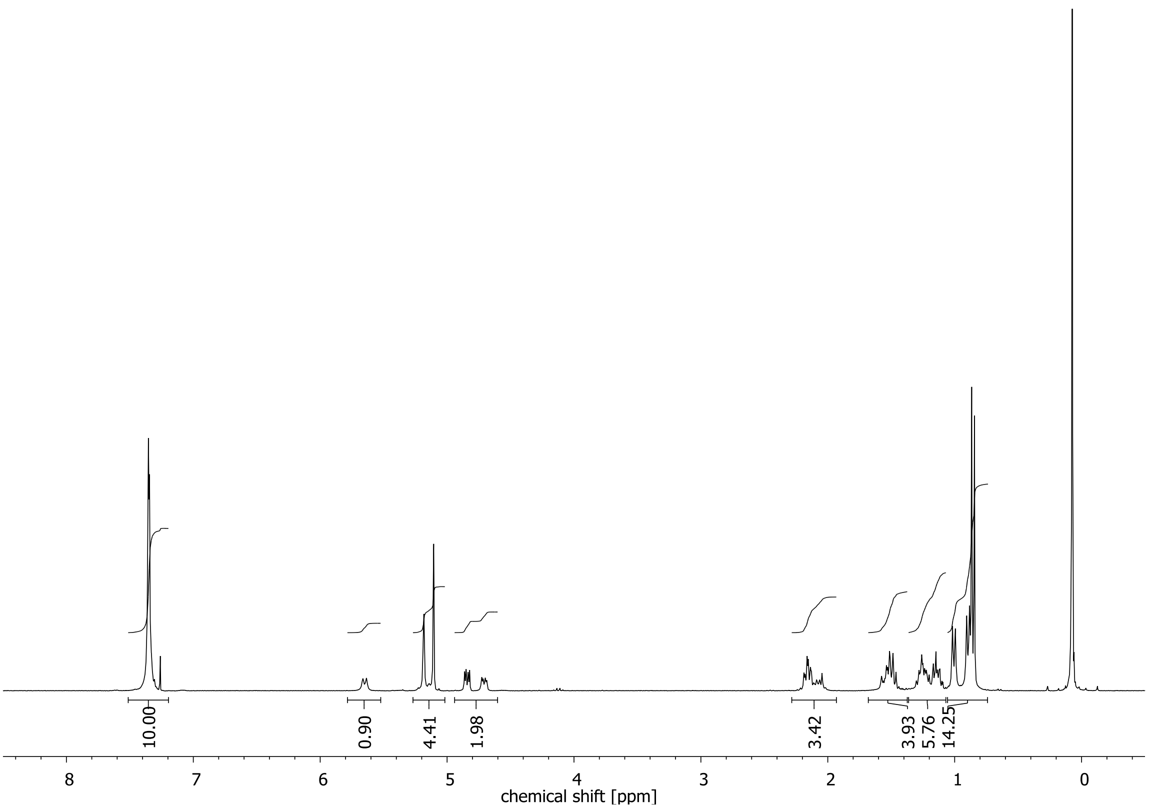

^{1}H NMR spectrum of **28** (300 MHz, $CDCl_3$)

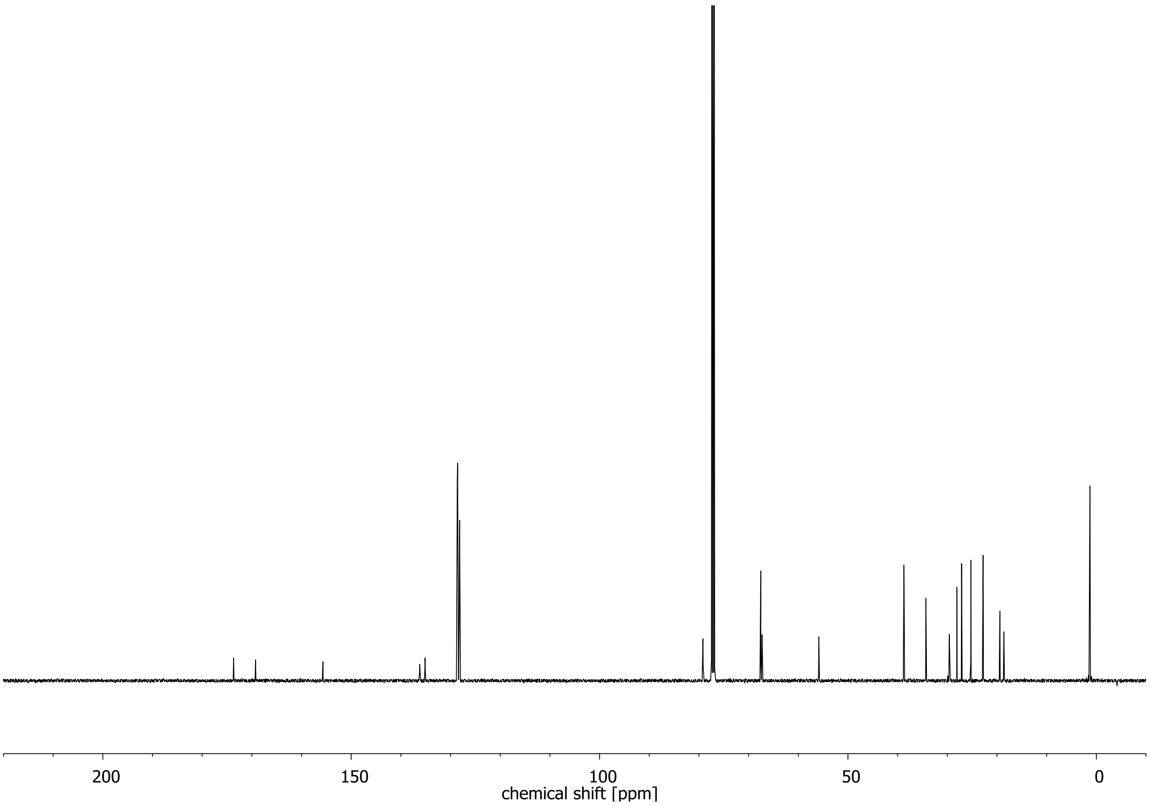

^{13}C NMR spectrum of **28** (126 MHz, $CDCl_3$)

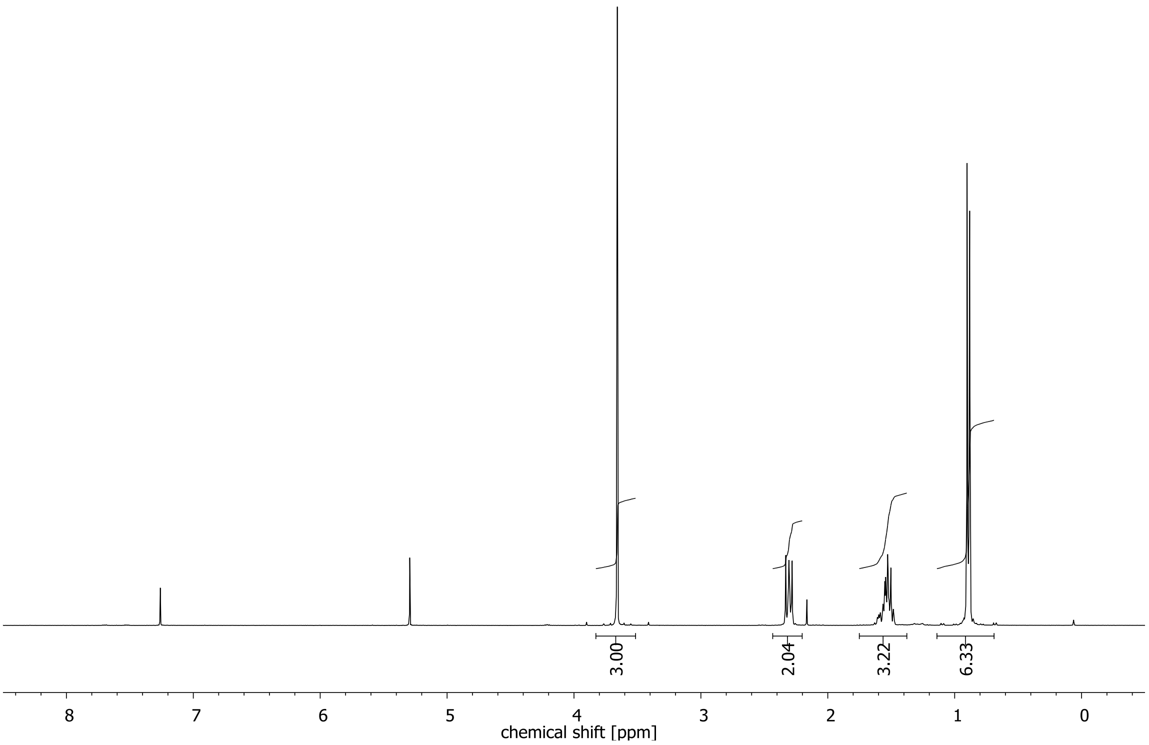

^{1}H NMR spectrum of **30** (300 MHz, $CDCl_3$)

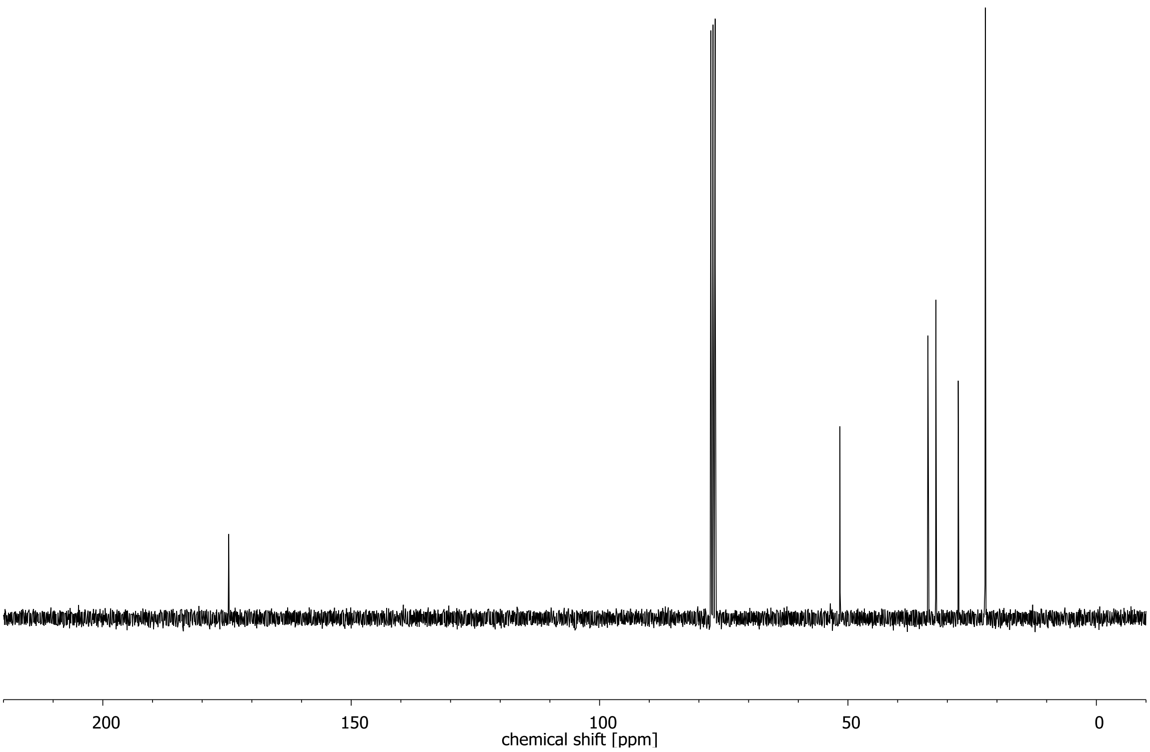

^{13}C NMR spectrum of **30** (76 MHz, $CDCl_3$)

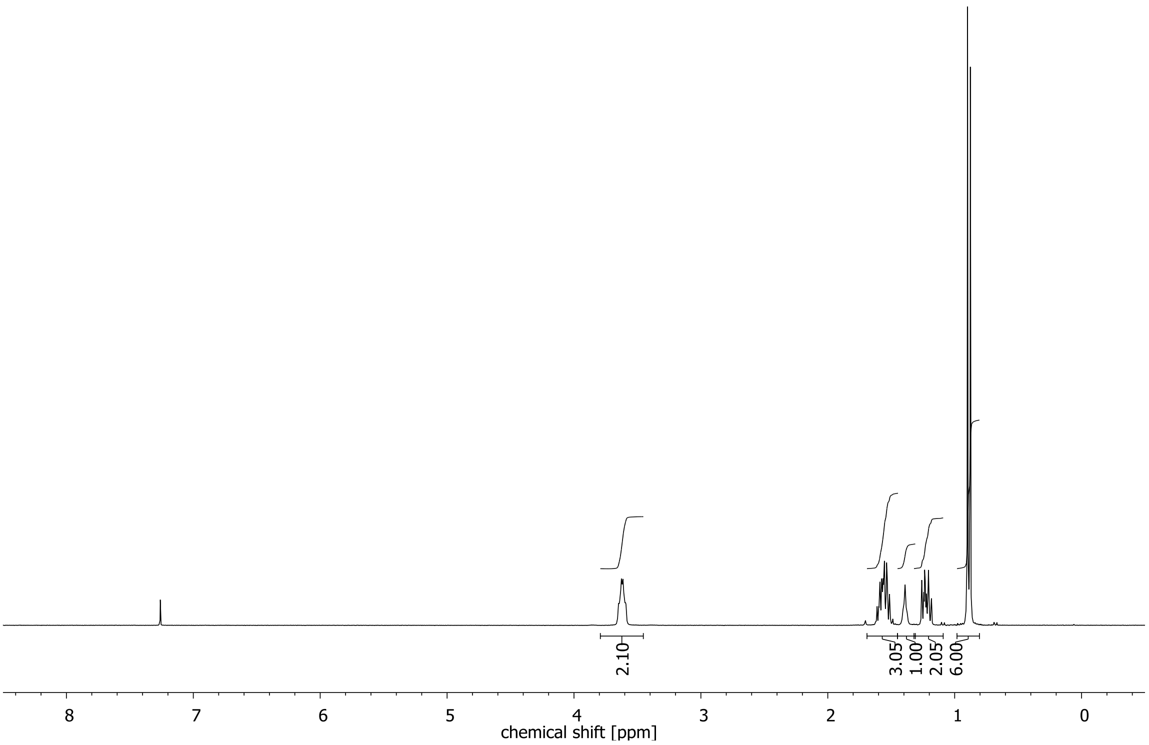

^{1}H NMR spectrum of **31** (300 MHz, $CDCl_3$)

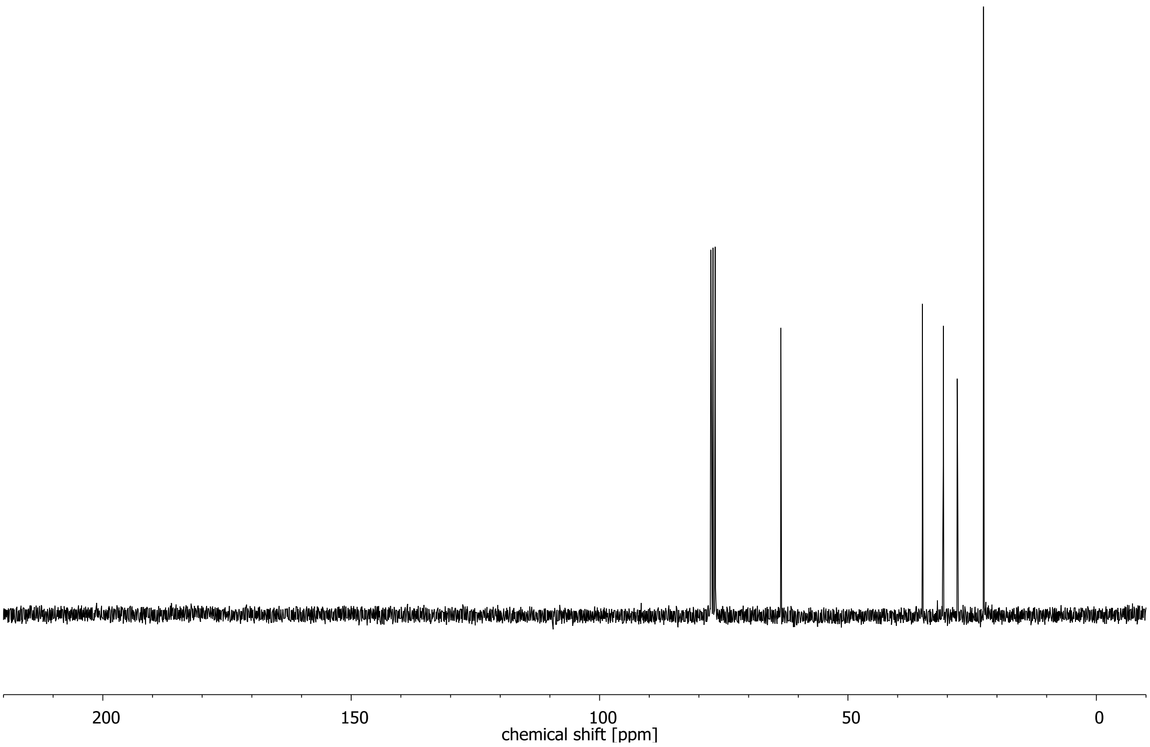

^{13}C NMR spectrum of **31** (75 MHz, $CDCl_3$)

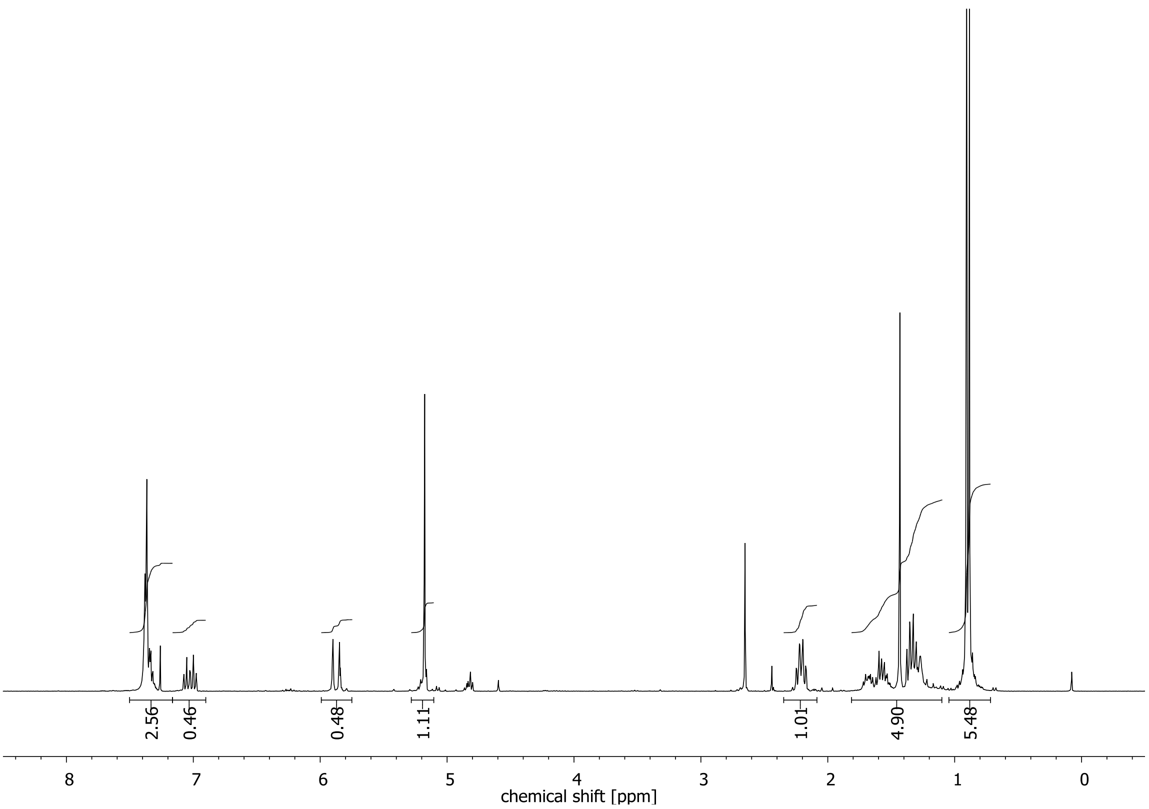

^{1}H NMR spectrum of **33** (300 MHz, $CDCl_3$)

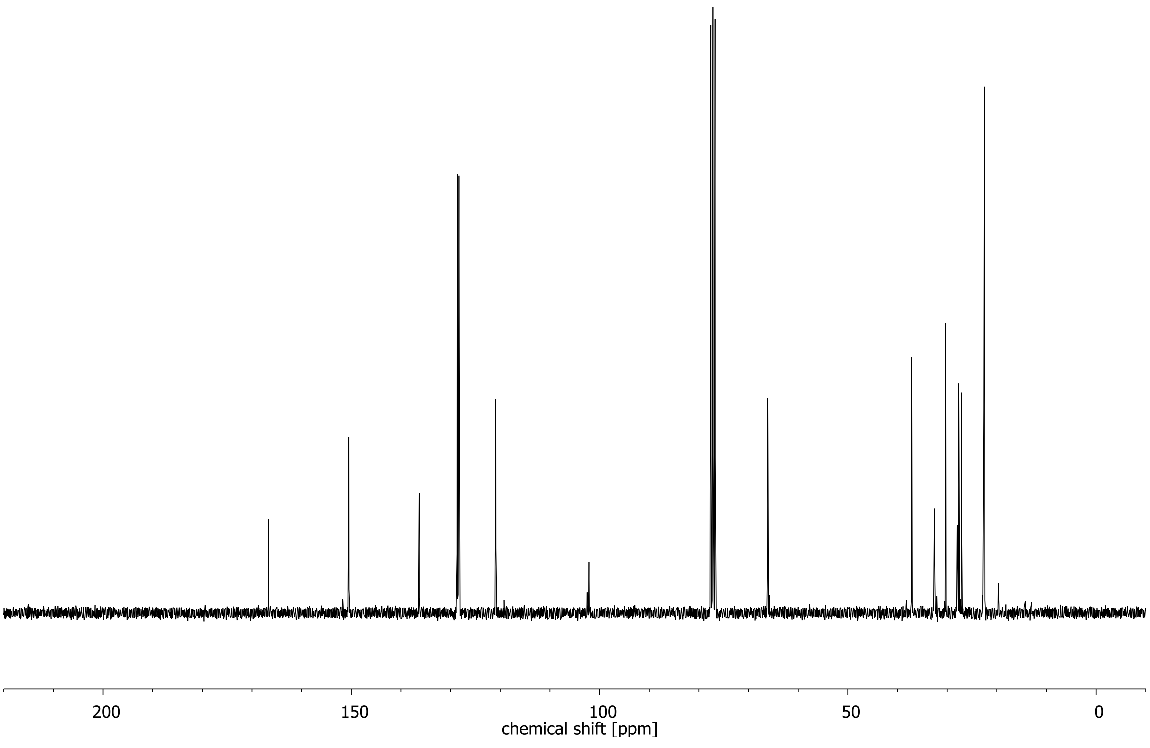

^{13}C NMR spectrum of **33** (75 MHz, $CDCl_3$)

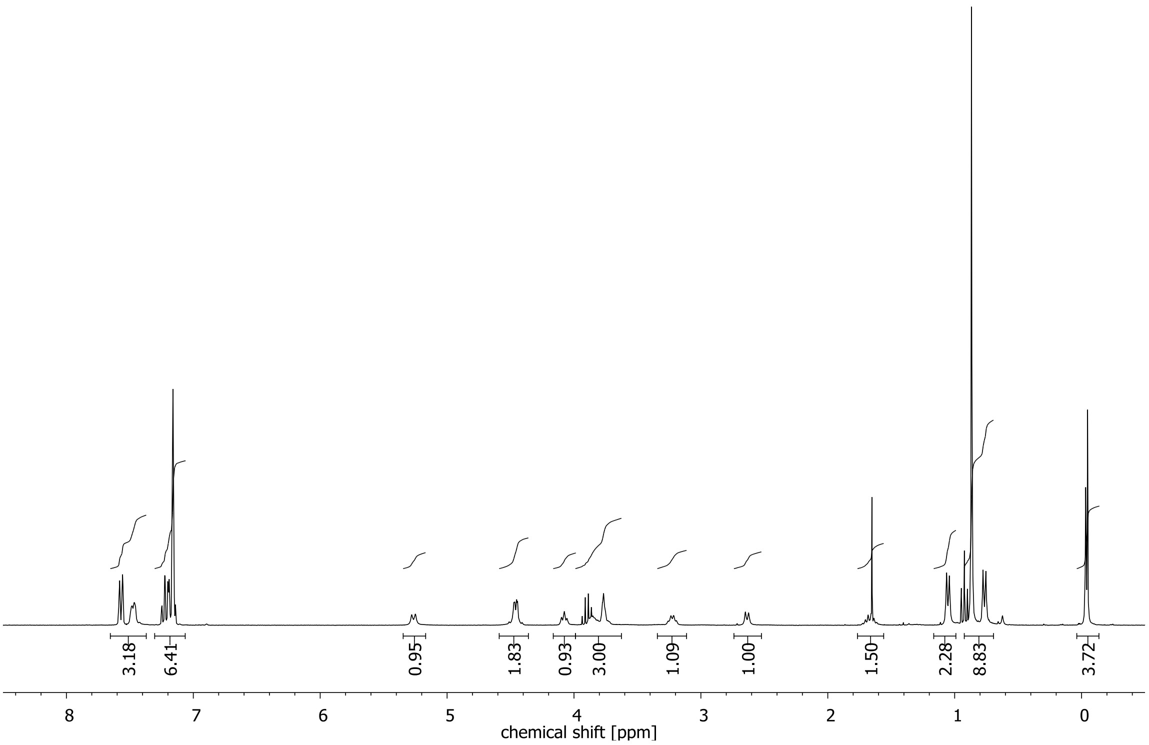

^{1}H NMR spectrum of **34** (300 MHz, C_6D_6)

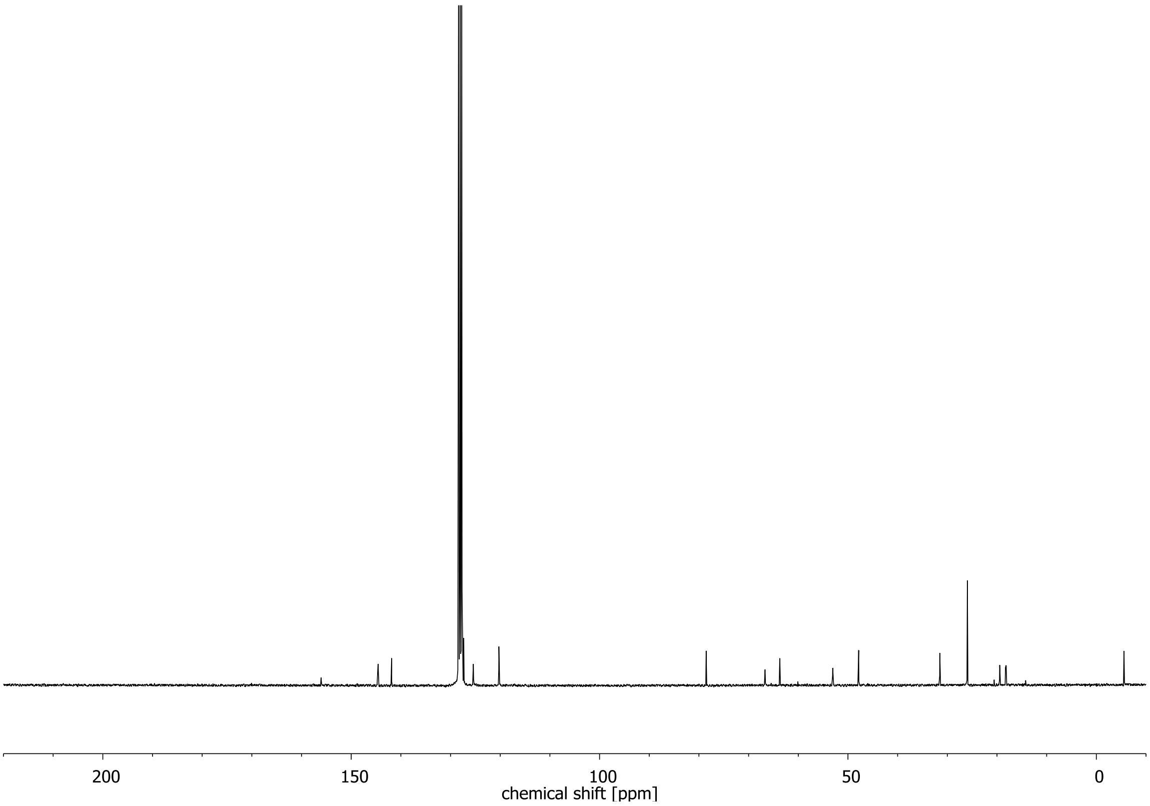

^{13}C NMR spectrum of **34** (76 MHz, C_6D_6)

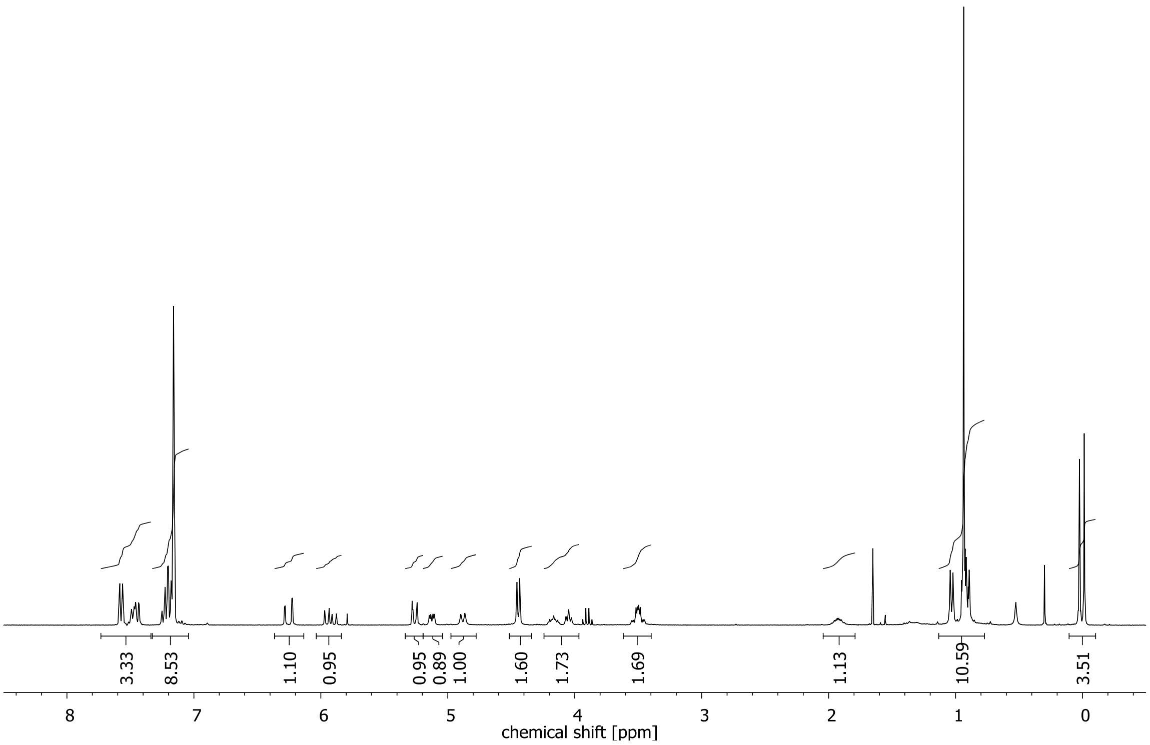

^{1}H NMR spectrum of **36** (300 MHz, C_6D_6)

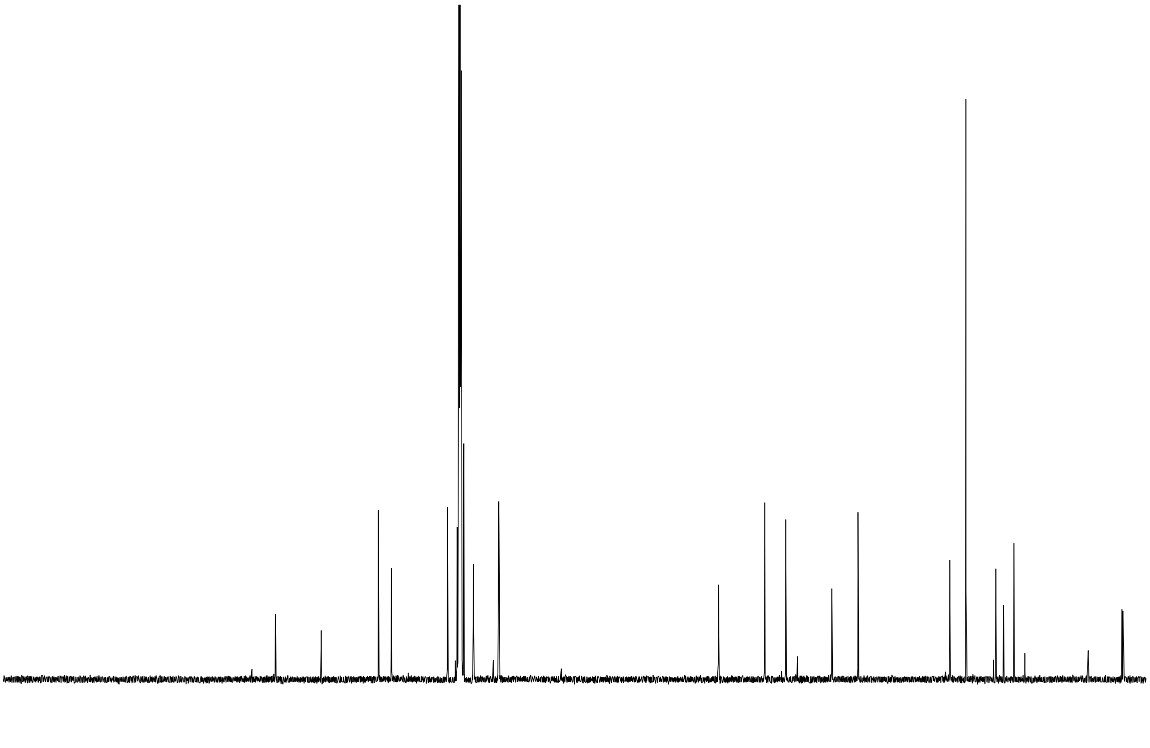

^{13}C NMR spectrum of **36** (76 MHz, C_6D_6)

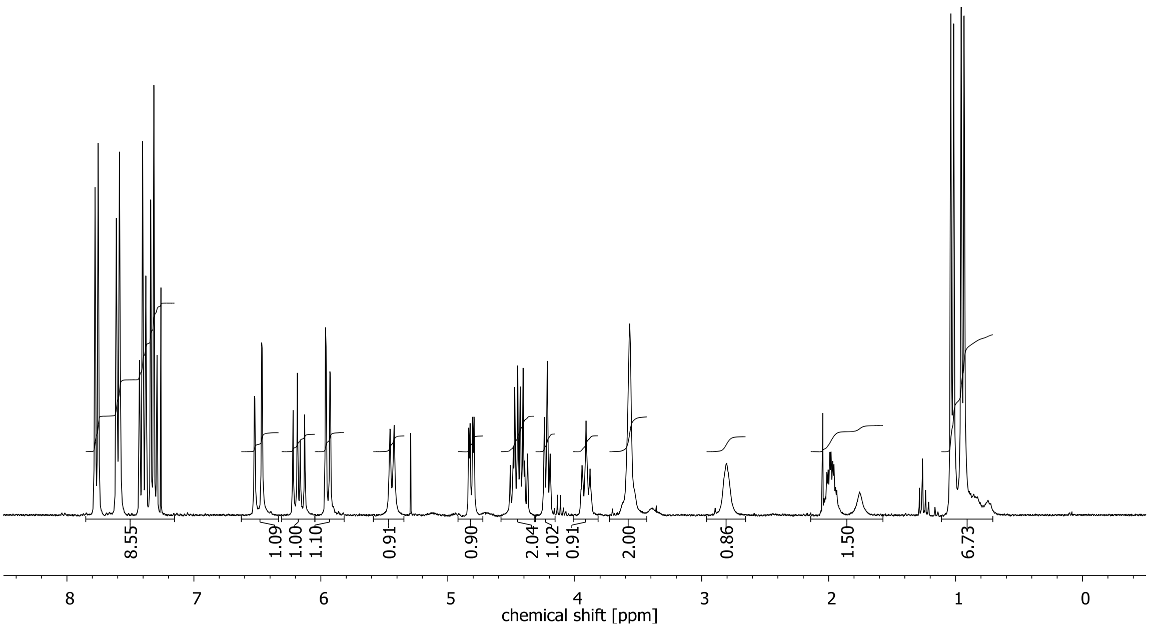

^{1}H NMR spectrum of **37** (300 MHz, $CDCl_3$)

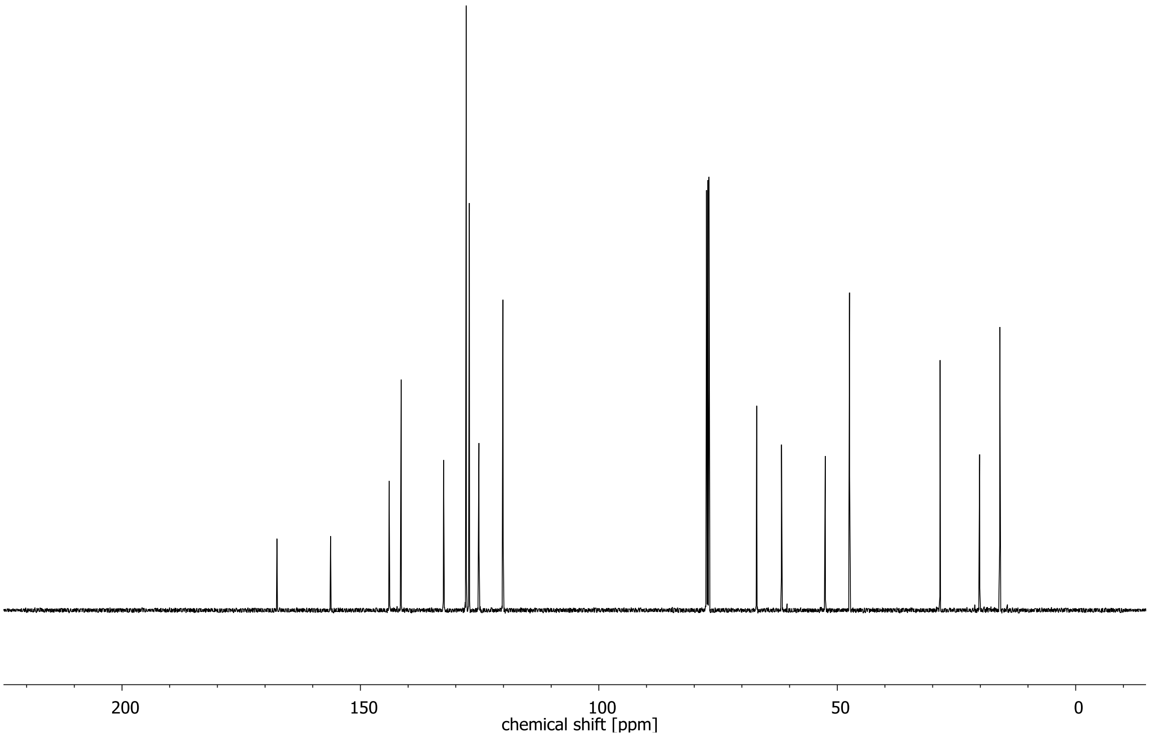

^{13}C NMR spectrum of **37** (126 MHz, $CDCl_3$)

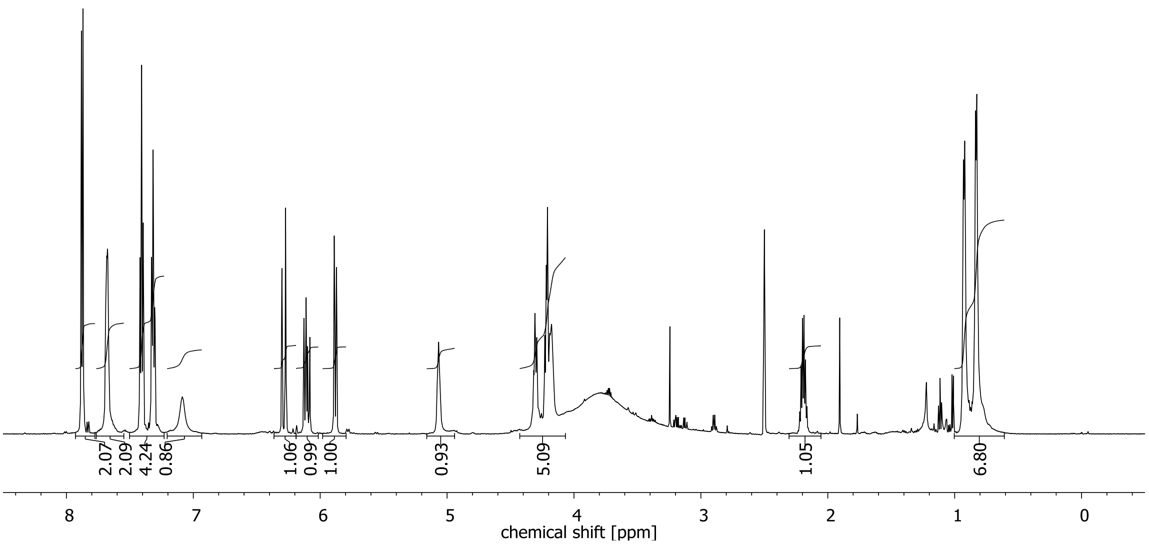

^{1}H NMR spectrum of **38** (600 MHz, DMSO-d_6)

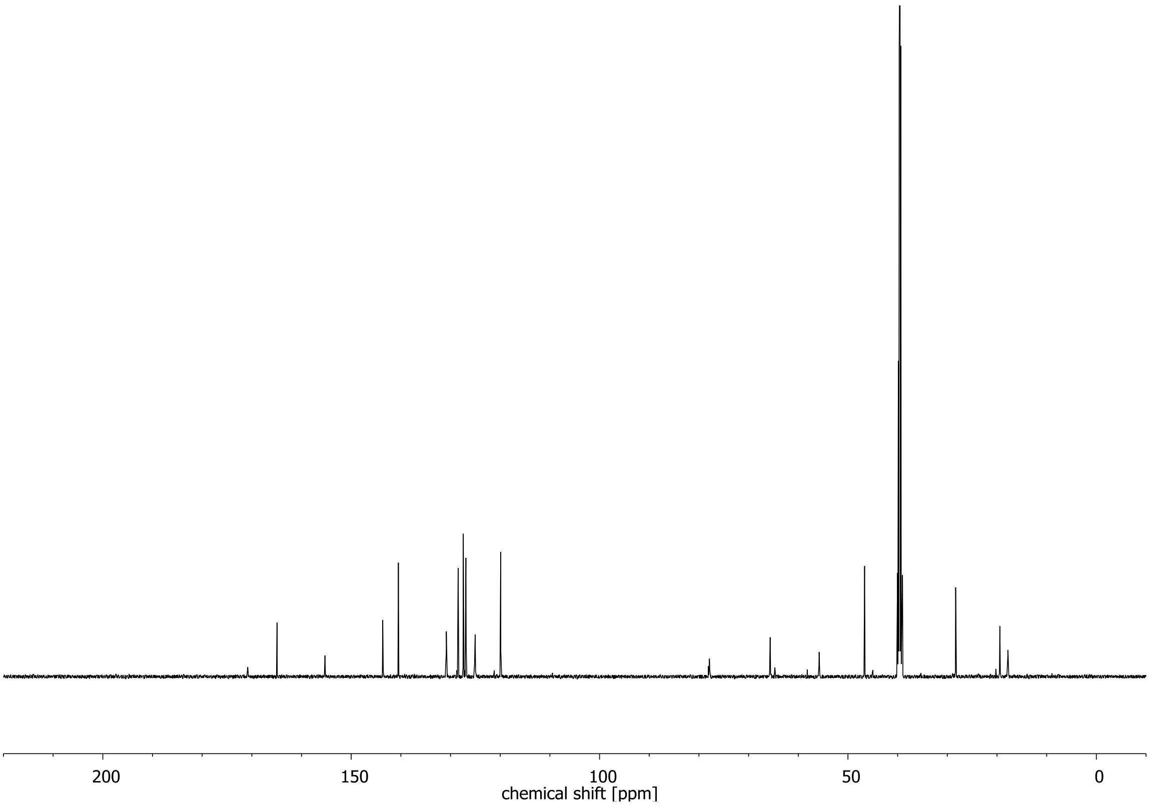

^{13}C NMR spectrum of **38** (126 MHz, DMSO-d_6)

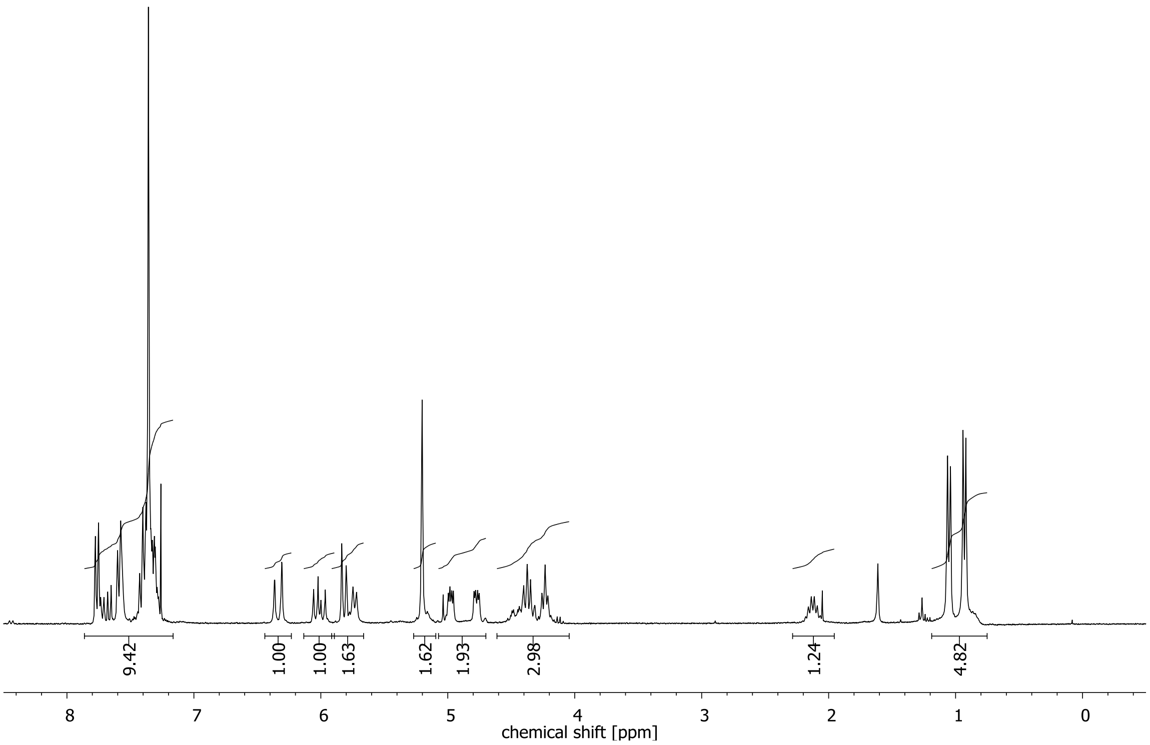

^{1}H NMR spectrum of **39** (300 MHz, $CDCl_3$)

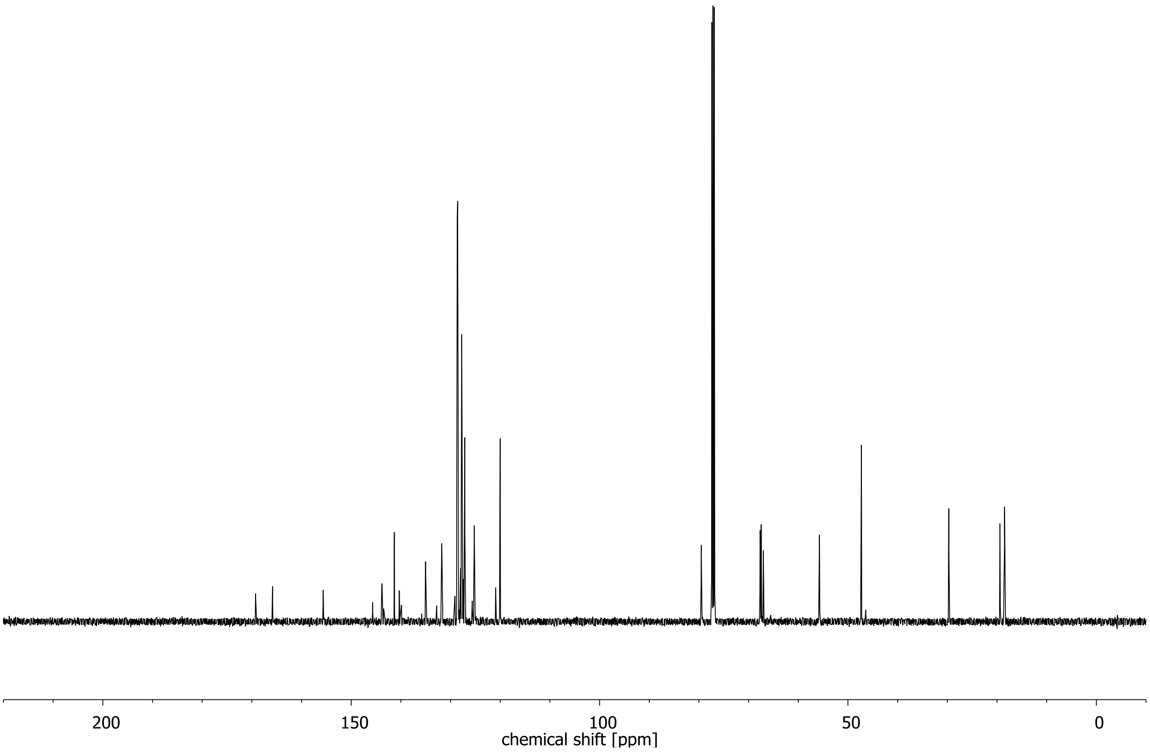

^{13}C NMR spectrum of **39** (126 MHz, $CDCl_3$)

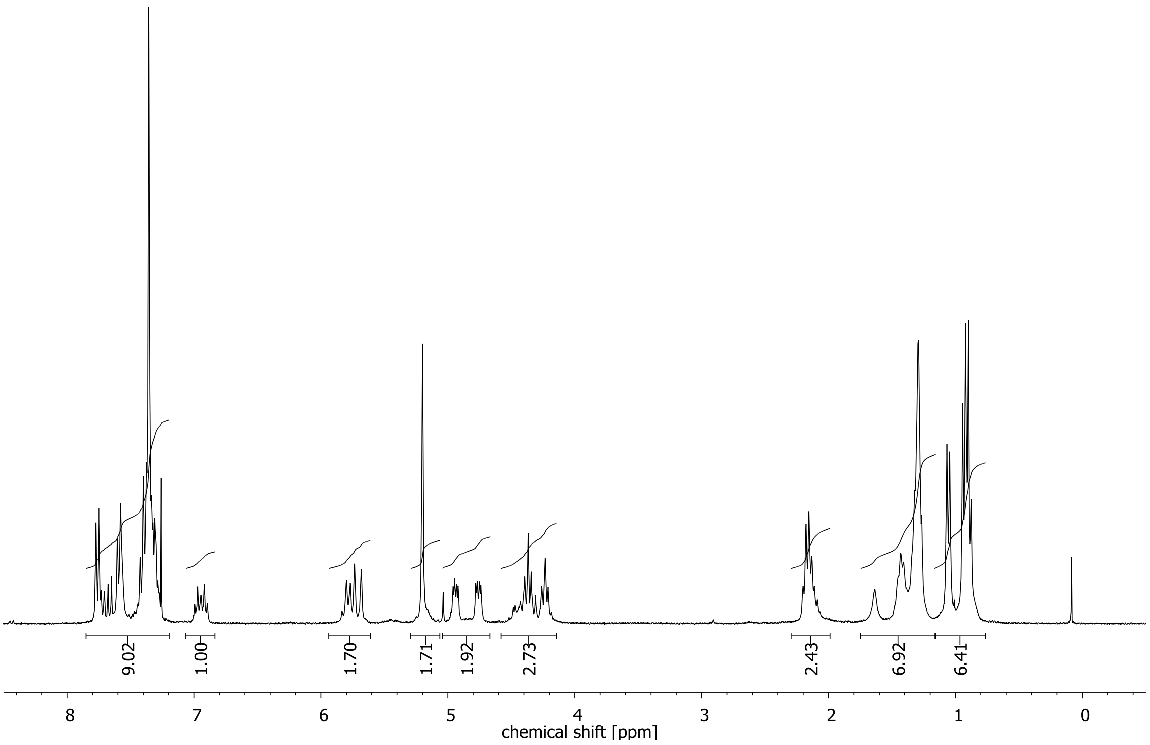

^{1}H NMR spectrum of **40** (300 MHz, $CDCl_3$)

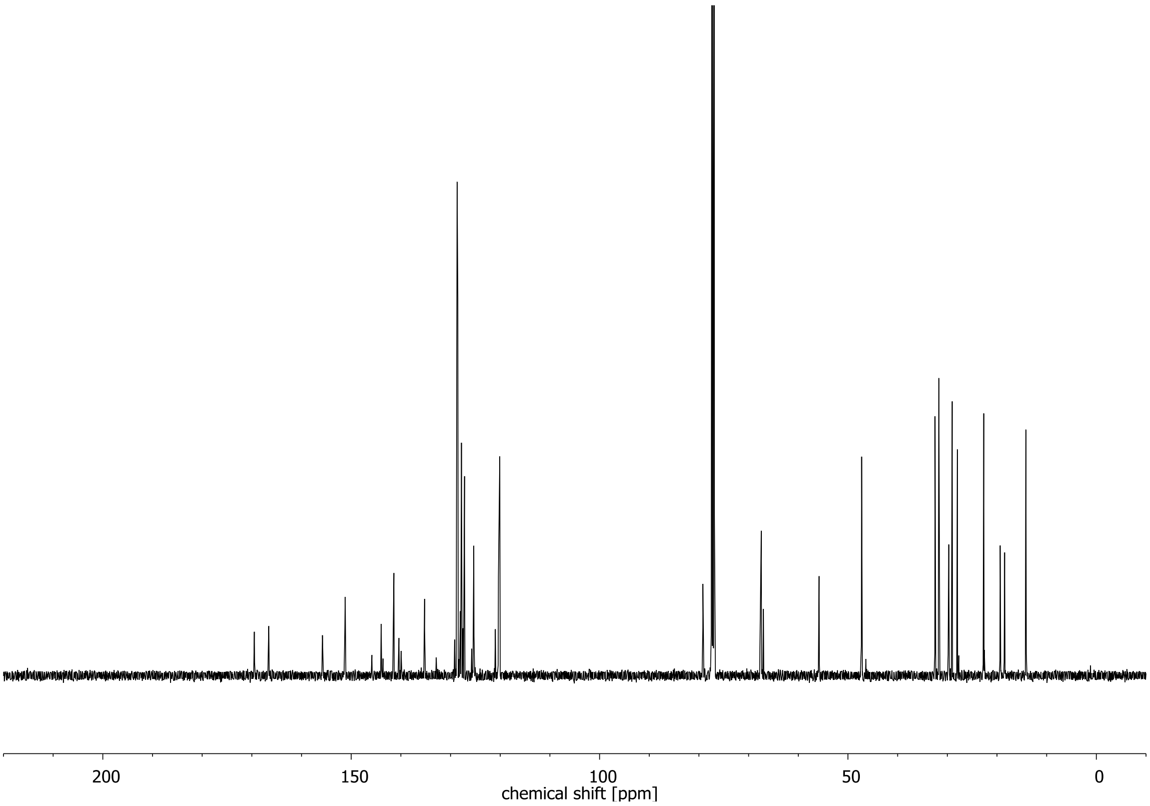

^{13}C NMR spectrum of **40** (126 MHz, $CDCl_3$)

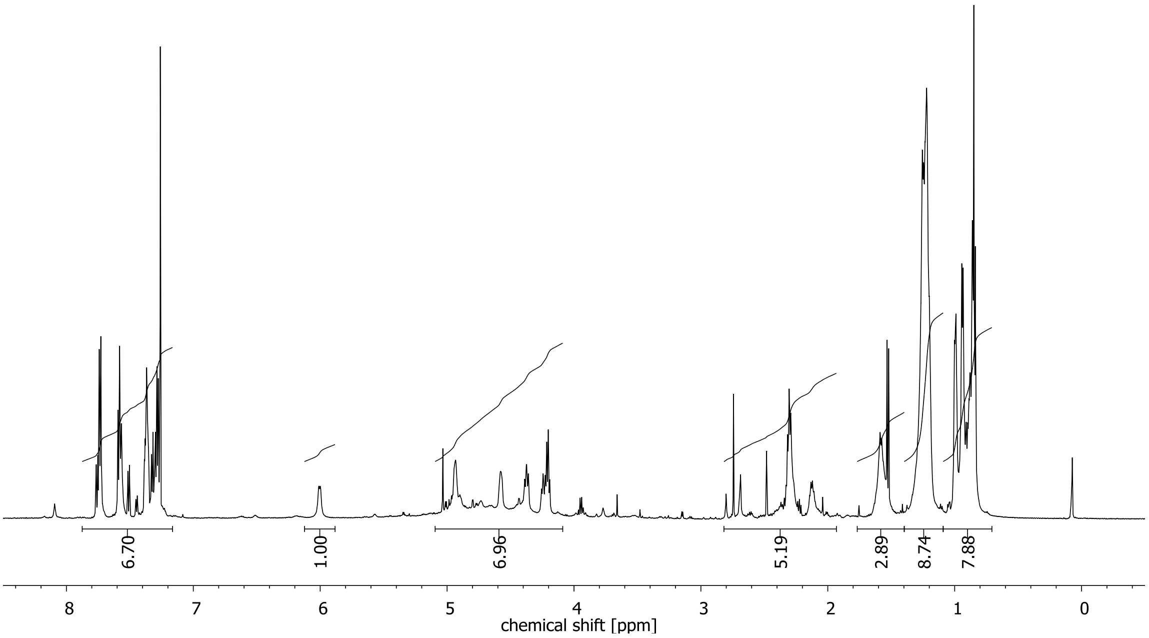

^{1}H NMR spectrum of **41** (600 MHz, $CDCl_3$)

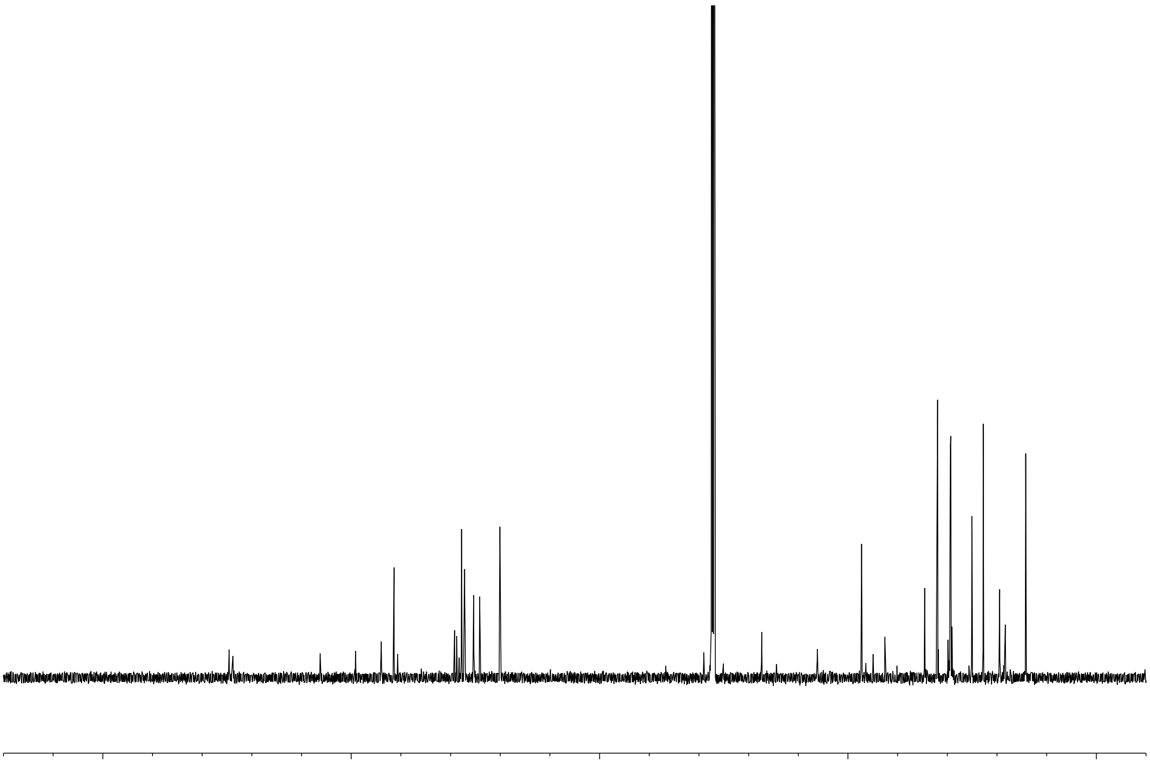

^{13}C NMR spectrum of **41** (126 MHz, $CDCl_3$)

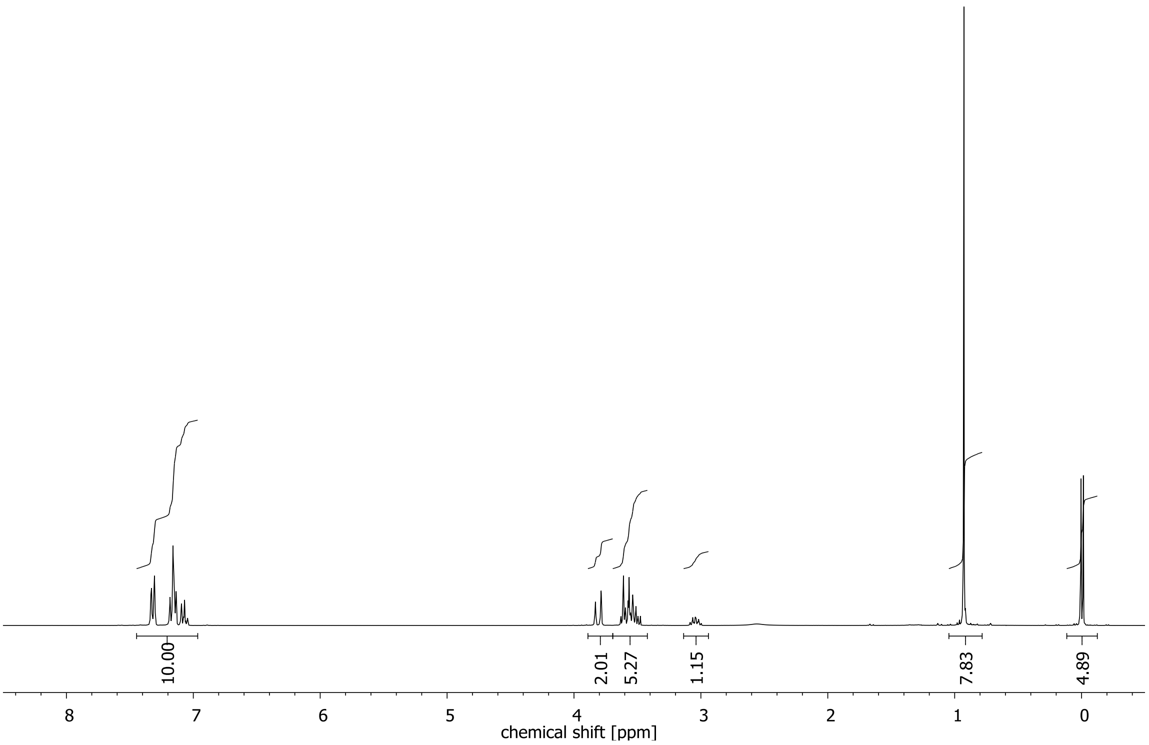

^{1}H NMR spectrum of **S1** (300 MHz, C_6D_6)

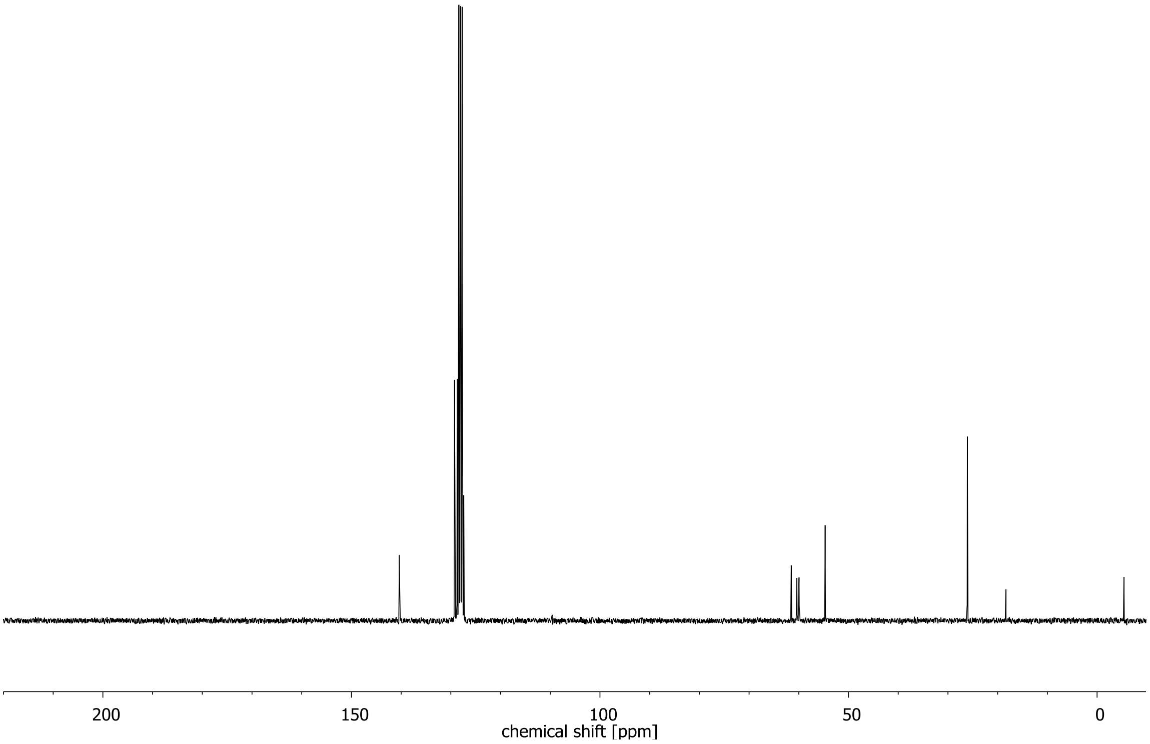

^{13}C NMR spectrum of **S1** (75 MHz, C_6D_6)

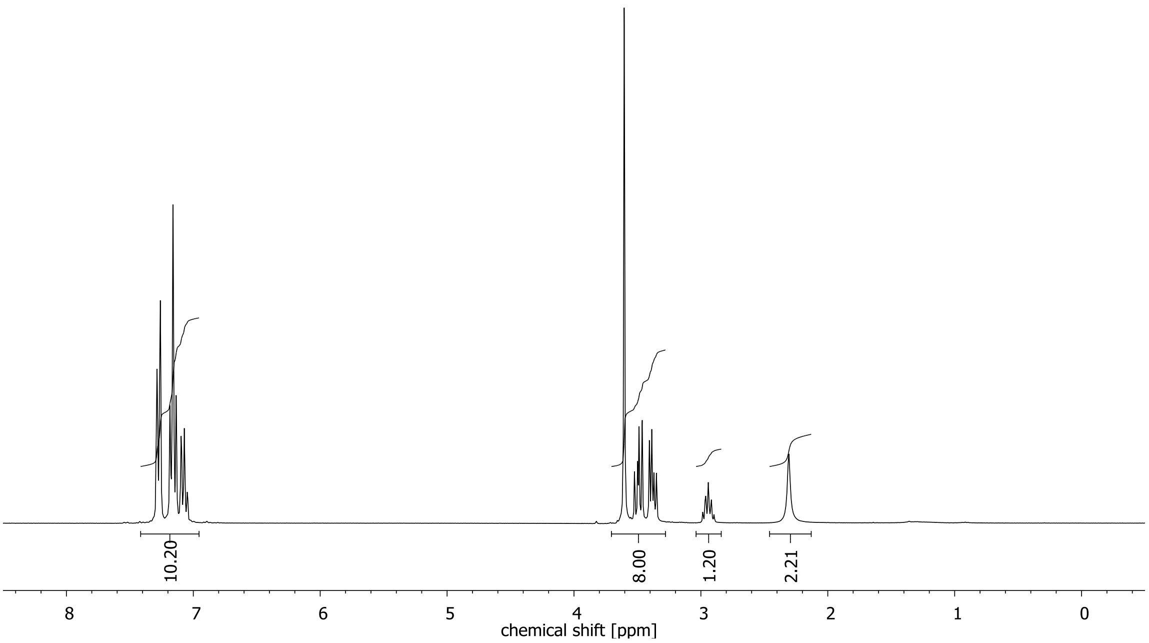

^{1}H NMR spectrum of **S3** (300 MHz, C_6D_6)

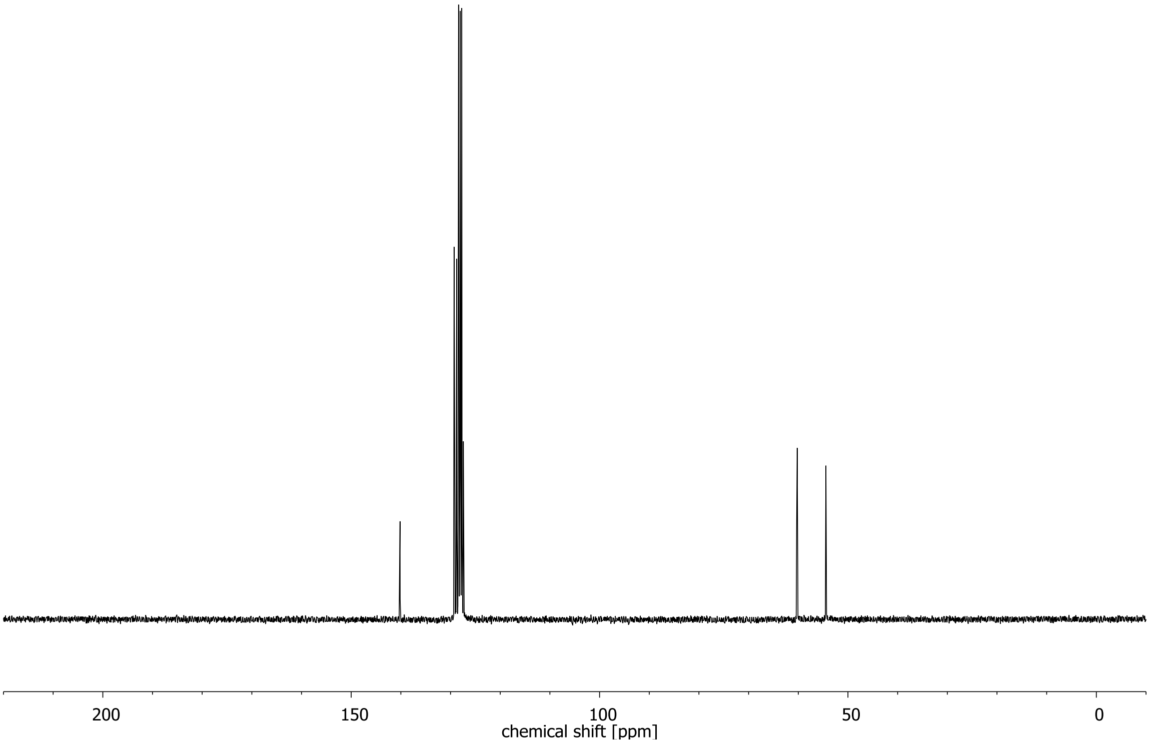

^{13}C NMR spectrum of **S3** (76 MHz, C_6D_6)

References

1. Stalke, D. *Chem. Soc. Rev.* **1998**, *27*, 171-178.

2. Kottke, T.; Stalke, D. *J. Appl. Crystallogr.* **1993**, *26*, 615-619.

3. Kottke, T.; Stalke, D. *J. Appl. Crystallogr.* **1996**, *29*, 465-468.

4. *SAINT*, v. 7.68A, Bruker APEX, rel. 2009/11; Bruker AXS, Madison/WI, **2009**.

5. Sheldrick, G. M. *SADABS*, rel. 2008/3, Georg-August-University Göttingen, Germany, **2008**.

6. Sheldrick, G. M. *Acta Crystallogr., Sect. A* **1990**, *46*, 467-473.

7. Sheldrick, G. M. *Acta Crystallogr., Sect. A* **2008**, *64*, 112-122.

8. Müller, P.; Herbst-Irmer, R.; Spek, A. L.; Schneider, T. R.; Sawaya, M. R. in: *Crystal Structure Refinement – A Crystallographer's Guide to SHELXL, IUCr Texts on Crystallography*, vol. 8 (Ed.: P. Müller), Oxford University Press, Oxford, UK, **2006**.
